# Activated immune cells present a lung interstitial‐to‐airway/alveolar cross‐compartment sharing pattern in severe pneumonia

**DOI:** 10.1002/ctm2.70696

**Published:** 2026-05-21

**Authors:** Yuean Zhao, Linjing Gong, He Yu, Sifan Zhang, Guanglei Yang, Chaoyang Wang, Weiya Wang, Xuyu Cai, Ye Wang

**Affiliations:** ^1^ Department of Pulmonary and Critical Care Medicine West China Hospital Sichuan University Chengdu Sichuan China; ^2^ State Key Laboratory of Respiratory Health and Multimorbidity West China Hospital, Sichuan University Chengdu Sichuan China; ^3^ Institute of Respiratory Health, Precision Medicine Center, Precision Medicine Key Laboratory of Sichuan Province West China Hospital, Sichuan University Chengdu Sichuan China; ^4^ Department of Neurosurgery West China Hospital, Sichuan University Chengdu Sichuan China; ^5^ Department of Pathology West China Hospital, Sichuan University Chengdu Sichuan China

**Keywords:** immune cells, immunology, severe pneumonia, single‐cell sequencing

## Abstract

**Background:**

Immune cells play a pivotal role in the pathogenesis of severe pneumonia. However, the global atlas of immune cells under this condition is not fully understood.

**Methods:**

We conducted single‐cell analyses of 275 411 cells isolated from matched lung tissue, bronchoalveolar lavage fluid, and peripheral blood from 12 patients with severe pneumonia and 5 donors, in order to identify the cross‐compartment sharing pattern of activated immune cells and the roles they play in severe pneumonia.

**Results:**

Our observations revealed that activated immune cells distributed across lung interstitia and airways (alveoli; Pattern 1a), including activated tissue‐resident memory‐like CD8^+^ T cells (T8_rms), plasma cells and plasmablasts, and the pro‐inflammatory macrophages. The activated T8_rms were derived from circulating CX3CR1^+^ CD8^+^ T cells, which upregulated ITGAE and CXCR3 during lung infiltration. Similarly, plasma cells and plasmablasts expressing ITGAE and CXCR3 also indicated the potential lung interstitial‐to‐airway/alveolar sharing pattern. The pro‐inflammatory macrophages interacted with T8_rms and plasma cells via the CXCL10‐CXCR3 axis.

**Conclusions:**

The lung interstitial‐to‐airway/alveolar cross‐compartment sharing pattern of activated immune cells (Pattern 1a) provide a robust working hypothesis for clinical and translational research for severe pneumonia.

## Graphical abstract

The interstitial‐to‐airway cross‐compartment sharing pattern of activated immune cells provides new insights for severe pneumonia. Macrophages could amplify local immune response by recruiting specific activated immune cells via CXCL10–CXCR3 signalling into airway, while other immune cells reside with limited inflammatory activity.

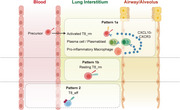

## 1 BACKGROUND

Pneumonia remains a predominant cause of morbidity and mortality globally, posing a significant public health challenge.^1^ It can be instigated by various pathogens, including bacteria, viruses and fungi, which may cause a wide spectrum of pulmonary and systemic effects and potentially lead to multiple organ dysfunction in severe cases.^2^, ^3^ Respiratory viruses, notably influenza and the recent SARS‐CoV‐2, are of particular concern due to their rapid transmission and substantial mortality associated with pandemic outbreaks.^4^, ^5^, ^6^, ^7^ Beyond the direct effects of pathogen invasion and toxin release, an excessively activated immune response can contribute to tissue damage and systemic inflammation.^1^, ^2^ Additionally, the infiltration of immune cells and inflammatory exudates into the lung interstitium and airway lumen significantly disrupts gas exchange, resulting in persistent and refractory hypoxemia.^8^, ^9^ Consequently, elucidating the distribution and interactions of immune cells during severe pneumonia is crucial for developing novel therapeutic strategies and improving patient outcomes.

Immune cells are typically present in the airways and alveoli, serving as primary sites for pathogen colonization and replication.^10^, ^11^ During pneumonia, their numbers increase markedly, reflecting either local proliferation or migration from the lung interstitium into the airways and alveoli.^12^, ^13^, ^14^ Notably, tissue‐resident memory (TRM)‐like CD8^+^ T cells (T8_rms) and pro‐inflammatory monocyte‐derived macrophages (MDMs) have been identified in the bronchoalveolar lavage fluid (BALF) of COVID‐19 patients,^15^, ^16^, ^17^, ^18^, ^19^ closely mirroring the immune cell profiles observed in autopsy lung specimens.^20^, ^21^, ^22^ Moreover, studies suggest that these activated immune cells may originate from the bloodstream and undergo phenotypic changes during their migration into the lung.^17^, ^19^, ^23^ In murine models, specific subsets of T and B cells infiltrate infection sites within the lung, with their migration potentially regulated by chemokine receptors such as CXCR6 and CXCR3.^3^, ^24^, ^25^, ^26^, ^27^

However, direct evidence detailing the precise migratory routes and phenotypic transformations of activated immune cells in human severe pneumonia is limited, particularly using matched peripheral blood, lung tissue and BALF samples. This knowledge gap hinders a comprehensive understanding of immune cell dynamics in the pathogenesis of severe pneumonia.

Emerging single‐cell multiomics technologies enable high‐resolution tracking of immune cell lineages in complex human diseases. Leveraging these approaches, we hypothesize that a subset of immune cells might migrate from the peripheral blood into the lung interstitium, where they become activated, proliferate, and undergo phenotypic transformations before subsequently transiting into the airways and alveoli during severe pneumonia.

To address this, we employed single‐cell transcriptomics and immune repertoire analyses on matched lung tissue, BALF and peripheral blood samples from patients with severe pneumonia. Our findings reveal a distinct cross‐compartment sharing pattern of activated immune cells, notably T8_rms and IgA^+^ plasma cells, recruited by pro‐inflammatory macrophages through mechanisms likely involving the CXCL10‐CXCR3 axis. These insights provide a vital foundation for further translational research aiming to develop novel therapeutic strategies for severe pneumonia.

## 2 METHODS

### 2.1 Study design

A total of 46 samples were included in this study. Matched lung specimens, BALFs, and peripheral blood mononuclear cells (PBMCs) were collected from 12 patients with severe pneumonia. Distal normal lung tissues and paired PBMCs were obtained from 5 control donors. No BALF was obtained from donors due to ethical considerations, for in clinical practice patients undergoing lung nodule wedge resection do not typically undergo bronchoscopy. Combined single‐cell RNA sequencing (scRNA‐seq), T cell receptor sequencing (TCR‐seq), and B cell receptor sequencing (BCR‐seq) were performed on most samples. Multiplex immunohistochemistry (mIHC) was conducted to determine the spatial distribution of inflammatory cells in the lung tissues. Flow cytometry was conducted for validation experiments.

### 2.2 Study approval

All procedures relating patient samples were in line with the ethical standards of the IRB and the Helsinki Declaration. Prior to sample collection, written informed consents were obtained from every participant in accordance with standard procedures. Ethical approval was obtained from the Institutional Review Board of West China Hospital of Sichuan University (approval number: 2021‐1250, 2021‐1650, 2022‐1395 and 2022‐1401).

### 2.3 Study subjects

All patients and donors were enrolled at West China Hospital of Sichuan University from December 2021 to May 2022. Severe pneumonia was defined according to the consensus guidelines of Infectious Diseases Society of America/American Thoracic Society.^28^ Patients admitted to the medical intensive care unit (MICU) were under mechanical ventilation, and their conditions were thoroughly evaluated by at least two respiratory physicians. Lung biopsy specimens were obtained following the BUS–PTNB workflow, which was published in our previous study.^29^ Donors were recruited among patients with suspected lung cancer and distal normal lung tissues were obtained during surgery. None of them underwent neoadjuvant therapy before surgery.

### 2.4 Methods and data analysis

Experimental procedure involved sample processing, scRNA‐seq sequencing, flow cytometry, multiplex fluorescence immunohistochemistry and metagenomic Next‐Generation Sequencing (mNGS). CellRanger (version 7.0.0, 10x Genomics) was used to quantify transcript. Downstream data analysis was conducted with R and Python. Low quality cells were defined as mitochondrial gene counts ratio over 10% or number of genes less than 200. The reciprocal PCA (RPCA) workflow was used for the integration of all individual datasets in order remove batch effects. Percentage of mitochondrial gene counts, percentage of erythrocytes gene counts and number of genes were regressed out during data scaling. Statistics were chosen based on data type. Statistical significance was defined as *p* values or adjusted p values less than .05. Details for experimental methods, data analysis and statistics are described in Supporting Information.

### 2.5 Statistics

Statistics were run in accordance with the legends of the figures. To calculate relationships between cell subsets, Pearson correlation was used. According to data's distribution, the Kruskal–Wallis test, Wilcoxon test and Student *T* test were used to compare means between groups. For adjusted *p* value calculation in finding DEGs, Benjamini–Hochberg technique was used to determine statistical significance. Statistical significance was defined as *p* values or adjusted p values less than .05.

## 3 Results

### 3.1 Experimental pipeline, clinical information and global analyses of cell composition

**FIGURE 1:**
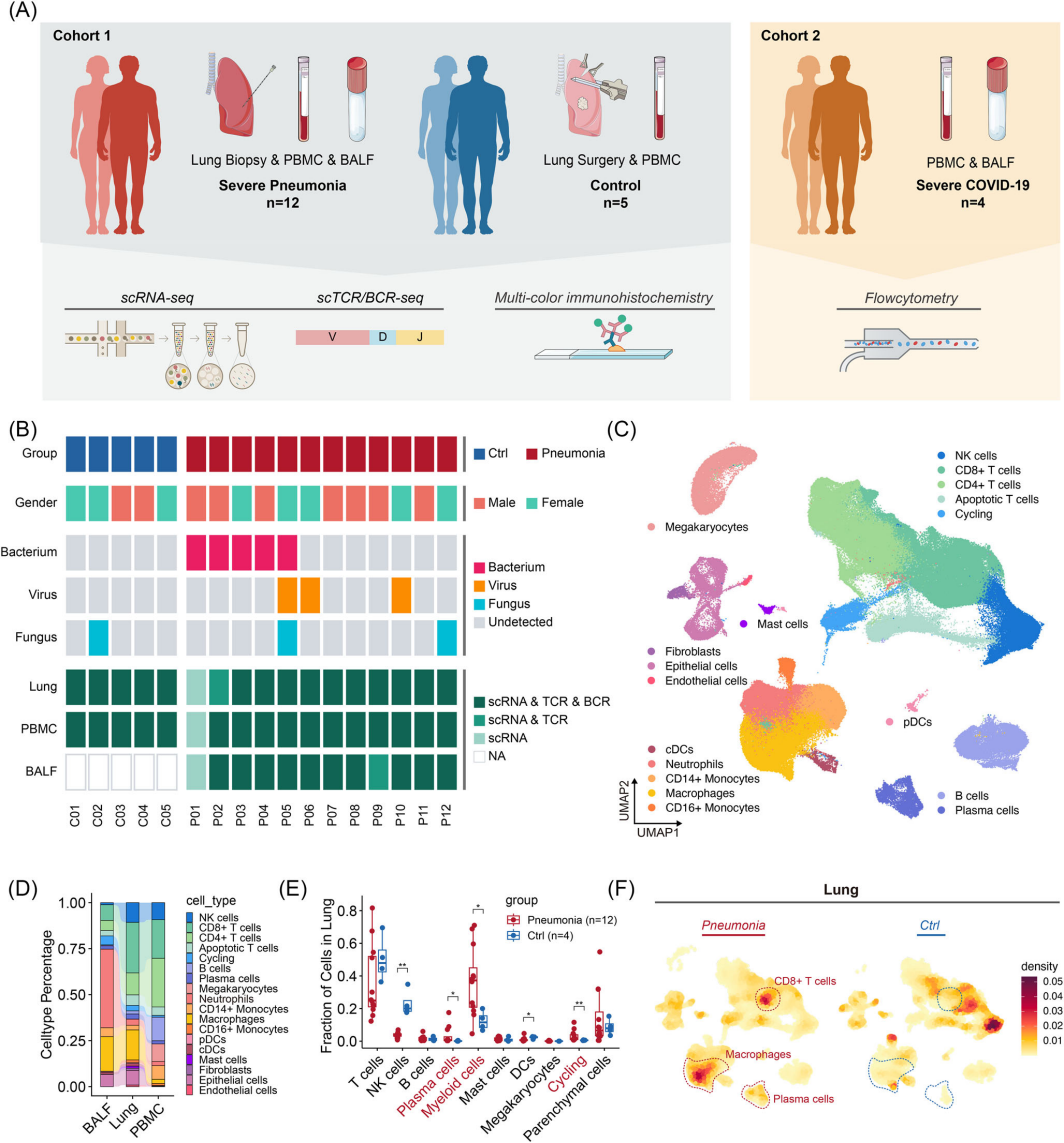
Experimental pipeline, clinical information and global analyses of cell composition. (A) Study design overview. (B) Clinical characteristics and sequencing details of patients and donors in Cohort 1. Infection type, background disease, pathological finding, and sequencing types are indicated. (C) UMAP embedding of 275 411 cells of 46 sample from 12 patients and 5 donors, assigned in 18 major cell type clusters. (D) Relative proportion of each cell type in Lung, BALF and PBMC. (e) 10 major cell type fractions in lungs of pneumonia group (*n* = 12) versus control group (*n* = 4). *p < 0.05; **p < 0.01. Wilcoxon rank‐sum test. *n* refers to the number of subjects. (F) Kernal density heatmaps showing the immune cell density in lungs of pneumonia and control groups. CD8^+^ T cells, macrophages and plasma cells are marked with dashed line due to distinct density between groups. Regions with high density are shown as dark red.

We enrolled a total of 12 patients with severe pneumonia and 5 control donors for the discovery cohort. Among the 12 patients, pathogens were identified in 8 cases: viruses were detected in 3 patients, bacteria in 5 patients and fungi in 2 patients. (Figure 1A,B; Table S1).

ScRNA‐seq was performed on cells from lung tissue, PBMCs and BALF samples, generating comprehensive atlases of cell populations (Figure 1C). A total of 275 411 cells passed quality control, comprising 174 456 cells from pneumonia patients and 100 955 cells from control donors. Uniform Manifold Approximation and Projection (UMAP) analysis of these filtered and normalized transcript counts revealed 18 distinct cell clusters (Figures 1C and S1C,D; Table S2). Due to the detection of *Pneumocystis jirovecii* in the mNGS analysis of lung tissues, one control donor (Donor 2) was excluded from further analyses of the control group.

The analysis of cellular composition across different sample types highlighted several key findings. Patients with bacterial infections exhibited an increase in neutrophils and macrophages in both lung tissue and BALF (Figures 1D and S1E). In contrast, a reduction in NK and T cell clusters was observed in the peripheral blood of these patients (Figure S1F). Notably, the lungs of pneumonia patients showed a marked increase in plasma cells, macrophages, and a subset of CD8^+^ T cells (Figure 1E,F). These findings suggest that plasma cells, macrophages and CD8^+^ T cells are crucial functional immune cells involved in the response to severe pneumonia.

### 3.2 T8_rms are highly activated and proliferated in both lung interstitia and airways (alveoli) in severe pneumonia

To elucidate the roles of CD8^+^ T cell subsets in severe pneumonia, we compared their composition and activation states between the pneumonia and control groups. Within the T/NK cell compartment (Figure S2A,B), we identified CD8^+^ T cells, CD4^+^ T cells, cycling T cells, unconventional T cells and NK cells.

**FIGURE 2:**
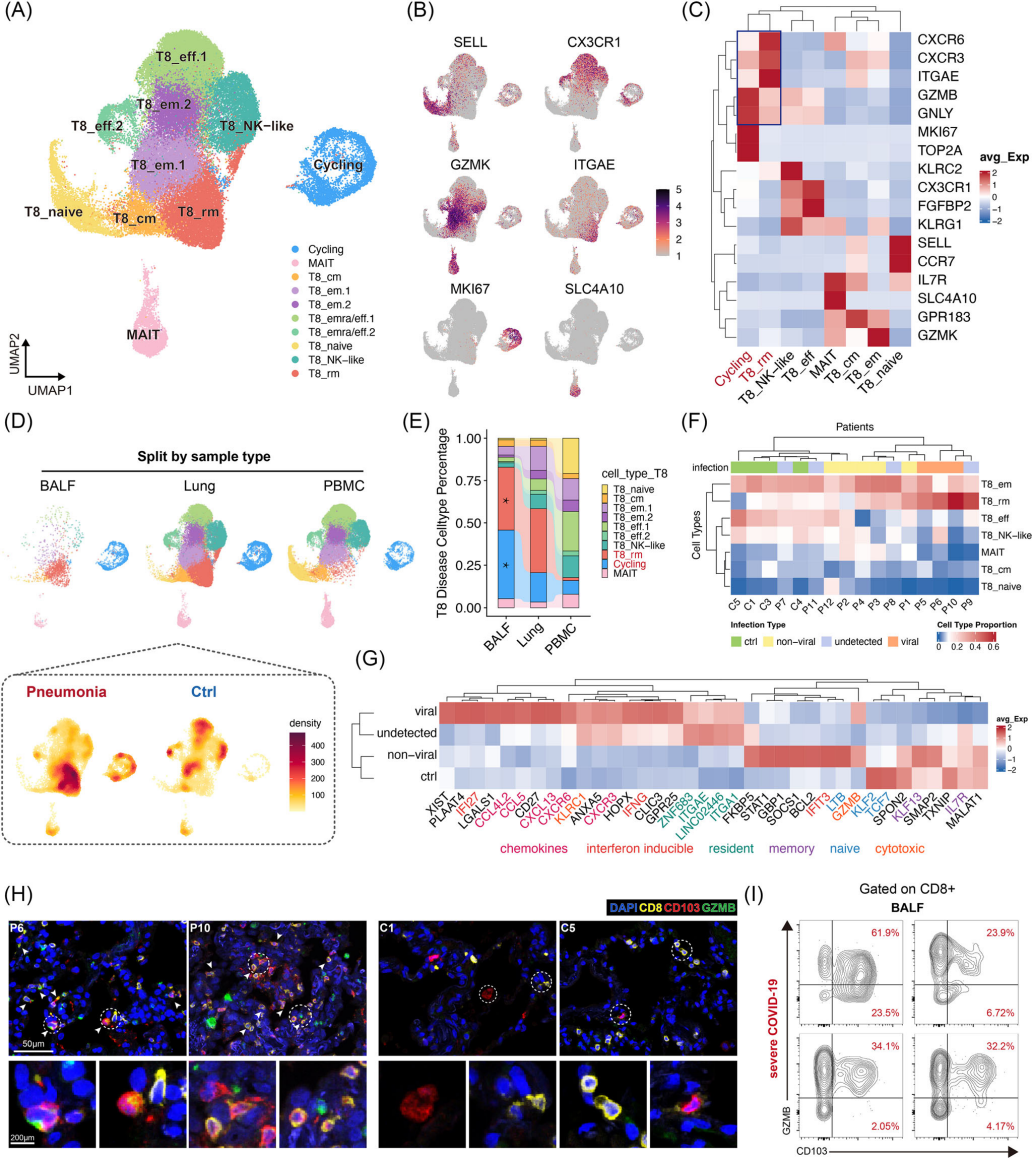
T8_rms are highly activated and proliferate in both lung interstitium and airways (alveoli) in severe pneumonia. (A) UMAP of 56 279 CD8^+^ T cells and cell type annotation of major clusters. (B) Normalized expression of marker genes for CD8^+^ T cells projected on the UMAP embedding. (C) Canonical gene expression among indicated CD8^+^ T cells subclusters. Columns and rows are both clustered. (D) Cell distributions across different sample types. The UMAP of lung was further split into pneumonia and ctrl groups, shown in cell density heatmap. (E) Relative proportion of each CD8^+^ T cell cluster in pneumonia group among lung, BALF and PBMC. (F) Heatmap showing cell type proportion in each patient with subgroups of viral infection annotated. (G) Average expression of indicated genes in patients among different subgroups of viral infection. (H) Higher lung infiltration of GZMB^+^ T8_rms in patients compared to controls. (I) Percentage of T cell subsets based on CD103 and GZMB expression in BALF of patients from Cohort 2.

We further subclustered T cells expressing CD8A, identifying 10 distinct clusters based on the expression of canonical T cell markers (Figure 2A–C,E). Among these, a cluster characterized by high expression of ITGAE and CXCR6 was identified as T8_rms, while two subsets marked by high expression of FGFBP2 and CX3CR1 were classified as T8_eff cell clusters. Notably, the T8_rms highly expressed cytotoxic markers such as GZMB and GNLY instead of memory marker (IL7R), which were named TRM‐like cells in some studies.^17^, ^19^, ^21^ Similar to previous findings,^30^, ^31^ T8_naïve and T8_eff were prevalent in PBMCs, while cycling T cells and T8_rms were predominant in BALF. Both cycling T cells and T8_rms constituted the principal cell clusters in pneumonia lungs and exhibited significant increases compared to control lungs (Figures 2D,E and S2C). Correlation analyses indicated that the proportion of T8_rms in pneumonia lungs was positively correlated with that of cycling T cells (Figure S2D). And these cycling cells expressing resident markers (Figure 2C) suggests that T8_rms were proliferating during pneumonia. The cell composition heatmap showed higher proportion of T8_rms in viral infected patients (Figure 2F), indicating its significant role against viral infection. T cells in lung samples with viral infection also have higher expression of cytotoxic, chemokines and interferon inducible markers, as compared to those in non‐viral infection and controls (Figure 2G and Table S3). Gene ontology (GO) analyses revealed extensive activation of multiple immune pathways in T8_rms from pneumonia patients (Table S4). Collectively, these findings highlight the high activation and proliferation of T8_rms during severe viral pneumonia, with widespread distribution across the lung interstitium and airway (alveolar) lumen. Furthermore, the gene signatures of T8_rms in patients without detectable pathogens resembled those in patients infected by viruses, albeit with higher IL7R and lower GZMB expression, suggesting potential recent viral infections (Figure 2G).

The mIHC of lung tissue revealed a significantly elevated proportion of GZMB^+^ CD103^+^ CD8^+^ T cells (activated T8_rms) in pneumonia group, with the majority of T8_rms localized in the airways (alveoli) (Figure 2H). Flow cytometry (FCM) analysis also demonstrated high proportions of activated T8_rms (Figures 2I and S2F) and an abundance of Ki67^+^ CD103^+^ CD8^+^ T cells in the BALF of COVID‐19 patients, while KLRG1^+^ CX3CR1^+^ CD8^+^ T cells in peripheral blood were significantly reduced compared to those from control donors (Figure S2G). These results align well with our single‐cell findings.

In addition to CD8^+^ T cells, CD4^+^ T cells also exhibited sample‐specific differences (Figure S2H,I and Table S2). Specifically, the proportions of T4_Th1 and T4_Treg were elevated in BALF and lung tissues, whereas T4_Tn and T4_CTL were more abundant in PBMCs (Figure S2J).

### 3.3 Activated T8_rms exhibit a lung interstitial‐to‐airway (alveolar) cross‐compartment sharing pattern

**FIGURE 3:**
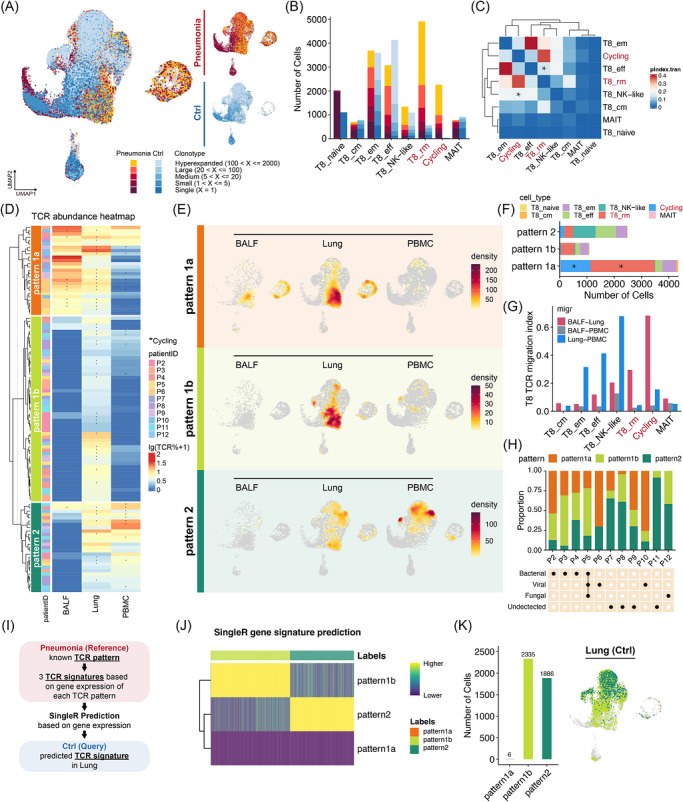
Activated T8_rms exhibit a lung interstitium‐to‐airway (alveolus) sharing pattern. (A) UMAP embedding of CD8^+^ T cells, coloured by TCR clone types. (B) The number of cells with TCR expansion in each CD8^+^ T subcluster among pneumonia and control groups. (C) The transition index among different CD8^+^ T cell subclusters. (D) Clustered TCR abundance among 3 sample types, revealing 3 TCR distribution patterns. Rows, TCR ID; left annotations, defined TCR patterns and patient origin; asterisk, TCR with cycling subcluster. (E) Projection of TCR pattern defined in (D) on the UMAP in the form of cell density. (F) Bar plots showing total cell number of each TCR pattern. (G) CD8^+^ T cell migration index among 3 sample types in each subcluster. (H) Proportion of TCR patterns in each patient, with their infection type annotated. (I) Schematic summary of singleR prediction process. (J) Predicted singleR scores for selected control lung cells. Column, each control lung cell to be predicted; row, defined TCR pattern of reference pneumonia TCR ID as in (d); top annotation, predicted TCR patterns (after fine‐tuning) for each cell. (K) Sum of each predicted TCR pattern and projection of predicted control lung TCR pattern on the UMAP. Both plots are coloured by TCR pattern.

To investigate the expansion and migratory behaviour of T cells, we analysed the lineage of clonal T cells using our scTCR‐seq data. We observed a larger clonal expansion within CD8^+^ T cell clusters compared to CD4^+^ T cells, leading us to focus our subsequent analyses on CD8^+^ T cells. Notably, TCR clones were significantly expanded in both T8_rms and cycling T cell clusters in the pneumonia group (Figure 3A,B), with a substantial overlap in TCR repertoire between these two clusters (Figure 3C), indicating a highly proliferative population of T8_rms.

Using the TCR sequencing data, we further explored the migratory characteristics of highly expanded CD8^+^ T cells across sample types (Figure 3D–F). We identified three principal TCR distribution patterns as shown in Table 1.

**TABLE 1:** Tissue and cell type distribution of TCR patterns.

TCR pattern	Tissue distribution			Cell type distribution
	Lung	PBMC	BALF	
Pattern 1a	√	×	√	T8_rms; Cycling
Pattern 1b	√	×	×	T8_rms
Pattern 2	√	√	×	T8_eff; T8_NK‐like

Pairwise STARTRAC‐migration analysis showed a high degree of TCR sharing between lung and BALF for T8_rms and cycling T cells, whereas TCRs from T8_em, T8_eff, and T8_NK‐like clusters exhibited a blood‐interstitium cross‐compartment sharing pattern (Figure 3G). CD8^+^ T cells in pattern 1a expressed higher levels of cytotoxic and proliferative genes compared to those in pattern 1b, suggesting that pattern 1a cells were activated and cytotoxic, while pattern 1b cells might represent a fraction of resting TRMs (Figure S3C,D and Table S3). Differentially expressed gene (DEG) analyses revealed increased tissue‐resident gene expression and decreased circulating effector gene levels in CD8^+^ T cells in patterns 1a and 1b compared to pattern 2 (Figure S3E,F and Table S3). Analysis of TCR pattern prevalence across patients revealed that Pattern 1a was most prominent in virally infected patients (P6 and P10), yet was also detectable in some patients with undetected pathogens (P9) and, to a lesser extent, in bacterially infected cases (P2, P3, and P4) (Figure 3H).

To infer the functional states of single cells in the control lungs, we used SingleR annotation scoring with transcriptomes of each TCR sharing pattern from the patients as reference datasets (Figure 3I). We found that most cells in the control lungs resembled the resting functional states similar to patterns 1b and 2 in patients, with almost none of pattern 1a representation (Figure 3J,K), indicating that the functional features of pattern 1a were specific to activated CD8^+^ cells during severe pneumonia.

Among CD4^+^ T cell clusters, T4_Th1 and cycling T cells exhibited significant clonal expansion in the patient group (Figure S3G,H). Cycling T cells were developmentally linked to T4_Th1, T4_Tfh, T4_Treg, and T4_CTL in the pneumonia group (Figure S3I). Additionally, cycling T cells, T4_Th1, T4_Tfh, and T4_Treg showed a lung‐to‐BALF TCR sharing pattern, whereas T4_CTL demonstrated substantial TCR sharing between PBMC and lung (Figure S3J), suggesting that CD4^+^ T cells may exhibit migratory patterns similar to those of CD8+ T cells.

These findings delineate a distinct profile of highly expanded T8_rms with an interstitial‐to‐airway (alveolar) cross‐compartment sharing pattern (pattern 1a) primarily identified in patients with severe pneumonia, while expanded circulating effector T cells were observable in the lung interstitium and peripheral blood (pattern 2) in both patients and controls.

### 3.4 Functional and transitional features of activated CD8+ T cells

**FIGURE 4:**
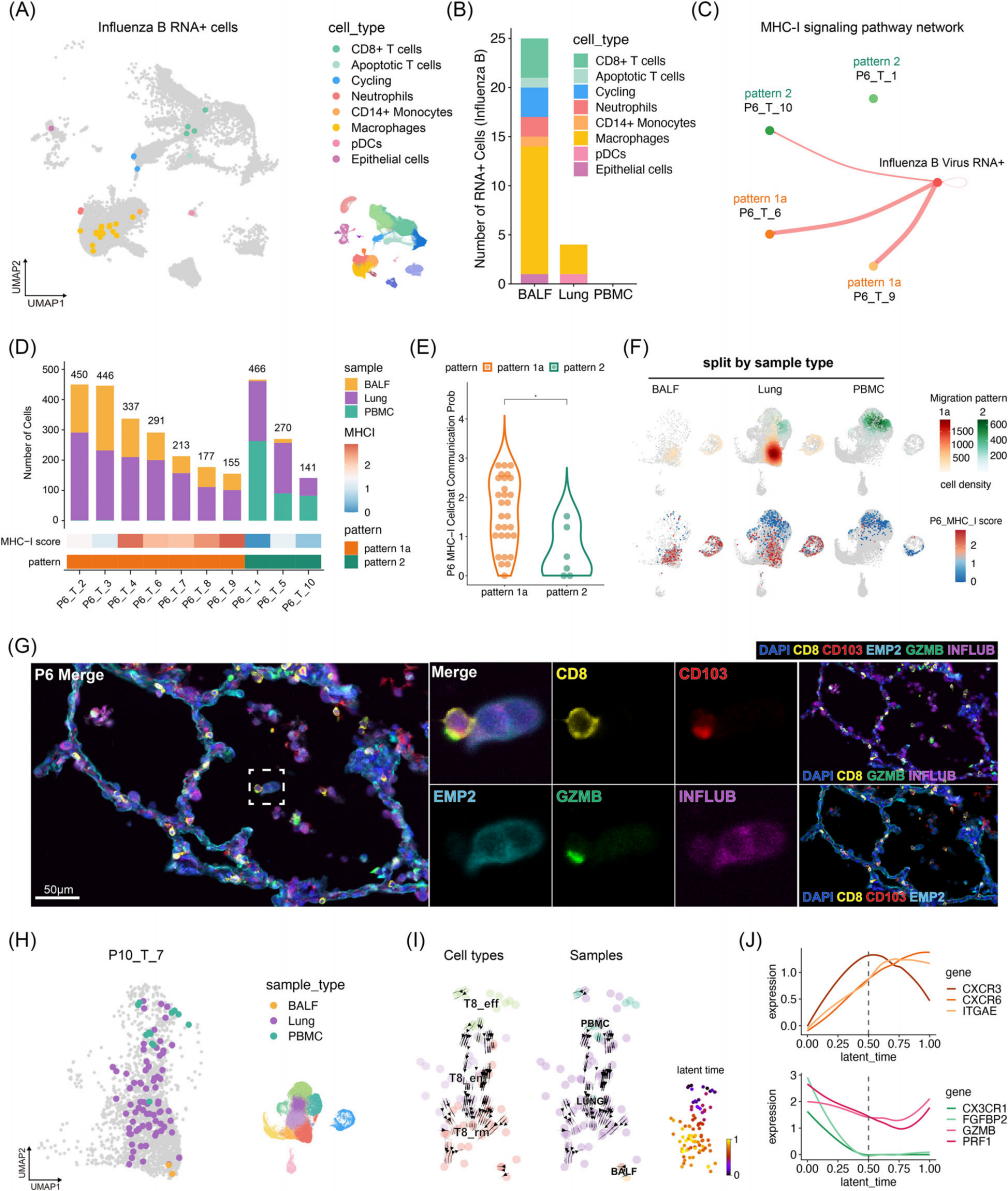
Functional and transitional features of activated CD8^+^ T cells. (A) Projection of cells with IBV RNA detected into UMAP embedding of P6 subset. (B) Sum of IBV RNA^+^ cells in Lung, BALF and PBMC separately. (C) Inferred cell‐cell interactions of IBV RNA^+^ cells with 4 typical TCR ID of pattern 1a and pattern 2. The thickness of lines indicates strength of interactions. (D) Sum of cell number and sample type distribution of P6's top 10 TCR ID, labelled with MHC‐I score and TCR pattern. (E) MHC‐I score between pattern 1a and pattern 2 of P6 selected TCR. *p < 0.05. Wilcoxon rank‐sum test. (F) Top, schematic TCR pattern distribution of each sample type on the UMAP, illustrated and coloured by cell density. Bottom, UMAP projection of selected P6 TCR with each cell coloured on the basis of MHC‐I score. (G) Representative mIHC images illustrating the interaction between activated T8_rms and IBV^+^ epithelial cells in the alveolar lumen of P6. (h) Distribution and sample type composition of P10_T_7. (I) Velocities derived from the dynamical model for TCR P10_T_7 are projected into a UMAP‐based embedding. Cells were coloured by samples and cell types, respectively. (J) Loess smooth curves showing 2 types of gene expression by latent time in (F).

Given the crucial role of CD8^+^ T cells in viral eradication, we focussed on the interplay between CD8^+^ T cell functionalities and their migratory patterns in a patient (P6) with influenza B virus (IBV) infection. Initial observations revealed a predominance of macrophages among IBV RNA^+^ cells, similar to findings in COVID‐19 patients, with a primary distribution in BALF (Figures 4A,B and S4A). Upregulated genes in IBV RNA^+^ cells were associated with cytokine and chemokine signalling, responses to interleukins, interferons, and intracellular pathogens (Figure S4A–C; and Tables S3, and S4).

We performed cell‐cell communication analysis between IBV RNA^+^ cells and representative CD8^+^ T cells categorized into patterns 1a (P6_T_6 and P6_T_9) and 2 (P6_T_1 and P6_T_10) (Figures 4C and S4D). CellChat analysis identified two distinct communication profiles between IBV RNA^+^ cells and T cells in different TCR patterns, highlighting 28 significant ligand‐receptor (L‐R) pairs. Most L‐R pairs, particularly those involving MHC‐I signalling, were more prominent in pattern 1a cells (Figure S4F). These findings indicate that T cells in pattern 1a, likely IBV‐specific, received robust TCR‐dependent signals. Enhanced MHC‐I signalling strength was observed in CD8^+^ T cells in pattern 1a, predominantly TRMs and cycling T cells, which exhibited an interstitial‐to‐airway cross‐compartment sharing pattern (Figure 4D–F). This suggests that activated T8_rms and cycling T cells, potentially virus‐specific, might migrate to the airway lumen and interact with IBV RNA^+^ cells via MHC‐I signalling. Co‐staining of lung specimens from patient P6 with antibodies against influenza B virions confirmed that activated T8_rms were in direct contact with IBV^+^ epithelial cells within the alveoli (Figure 4G), suggesting potential cytotoxic engagement of IBV^+^ cells by T8_rms.

We also investigated the phenotypic transformation of TRM precursors into T8_rms during infiltration. We identified a transitioning TCR clone from patient P10 (Figure 4H; P10_T_7), which was present in the lung, BALF, and PBMC. RNA velocity analysis of this TCR clone predicted a typical conversion trajectory from CX3CR1^+^ T8 cells in PBMCs to T8_rms in the lung and BALF (Figure 4I). During this transition, we observed a notable upregulation of the chemokine receptor gene CXCR3 during the initial phase, followed by a rapid downregulation of CX3CR1 and FGFBP2. The second phase was characterized by downregulation of CXCR3, consistent with previous studies. The expression levels of tissue‐resident genes ITGAE and CXCR6 gradually increased and peaked at the end of the transition period (Figure 4J). These findings suggest that the shift in CX3CR1 and CXCR3 expression could serve as a hallmark for the early transition from CX3CR1^+^ T8 cells to T8_rms, with modulation of CXCR3 playing a crucial role in enabling TRM precursors to migrate into the lung interstitium and airway lumen.

### 3.5 Plasmablasts and IgA+ plasma cells are enriched in the airway (alveolar) lumen

**FIGURE 5:**
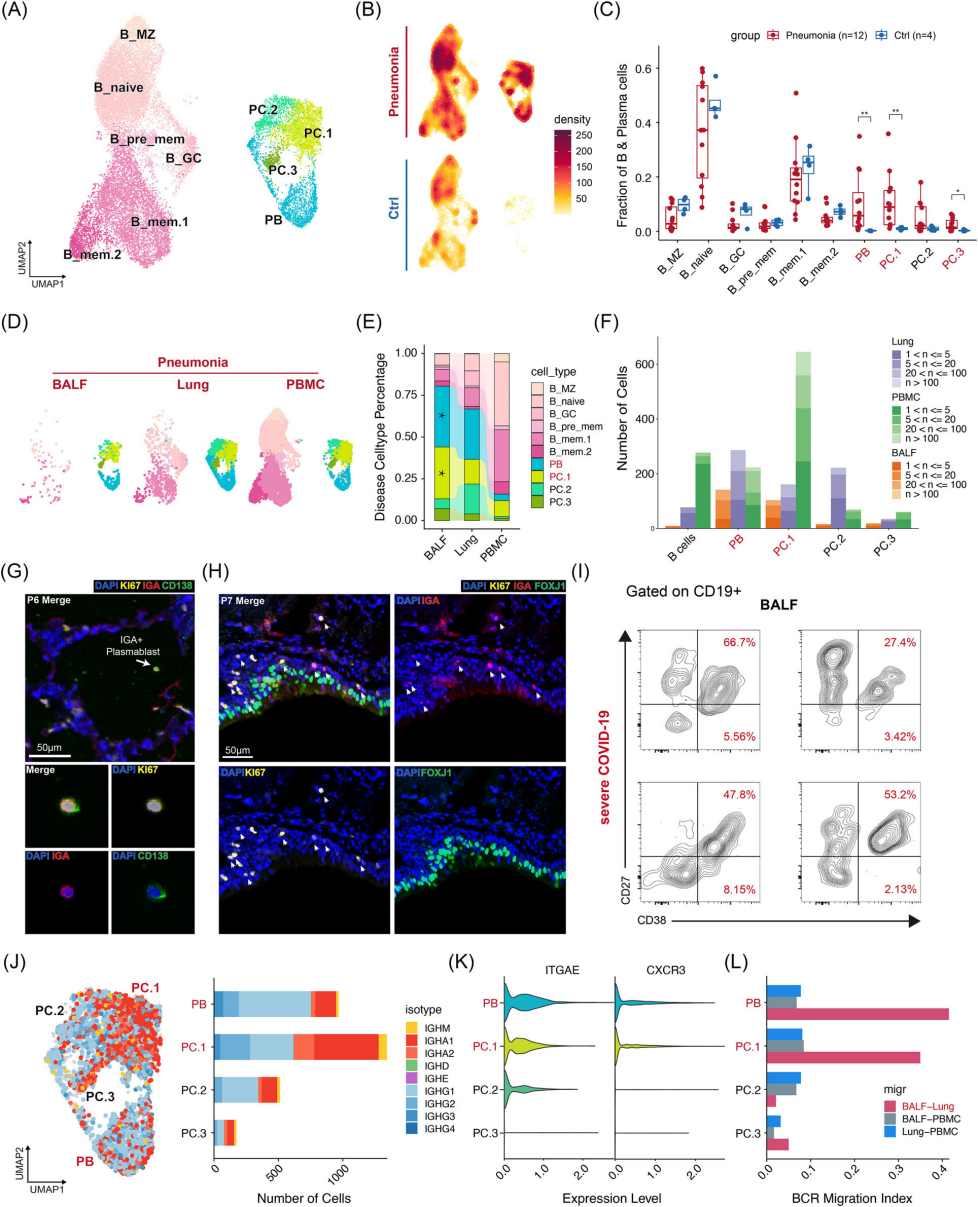
Plasmablasts and IgA^+^ plasma cells are enriched in the airway (alveolar) lumen. (A) UMAP of 20 817 B and plasma cells. (B) Density distribution of B and plasma cells. (C) B and plasma subcluster fractions in the pneumonia versus control lungs. *p < 0.05; **p < 0.01. Wilcoxon rank‐sum test. n refers to the number of subjects. (D) B and plasma subcluster distributions across different sample types. (E) Relative proportion of each B‐lineage cell subcluster in pneumonia group among sample types. (F) The number of cells with BCR expansion in pneumonia group. All B cell subtypes are integrated together. (G) The presence of Ki67^+^ IgA^+^ CD138^+^ plasma cells in the alveolar lumen of P6. (H) IgA^+^ Ki67^+^ cells and diffused IgA antibodies distributing in the mucosal region of P7. (I) Percentage of plasma cell subsets based on CD27 and CD38 expression in BALF of patients from cohort 2. (J) BCR isotype distribution. Left, UMAP of plasma cells as in (A). Right, total cell number of each BCR isotype. Both coloured by BCR isotype. (K) ITGAE and CXCR3 log‐normalized mRNA expression of PB and plasma cells in pneumonia and control lungs, respectively. (L) Plasma cell migration index among 3 sample types in each subcluster.

In addition to T cells, we observed a significant increase in plasma cells in patients with severe pneumonia (Figure 1E,F). From the scRNA‐seq data, 20 817 B‐lineage cells passed quality control for subsequent analyses. We identified ten cell clusters, including six clusters of B cells, three clusters of plasma cells (PC.1, PC.2, PC.3), and one cluster of plasmablasts (PB) (Figures 5A and S5A,B). The proportions of PB and plasma cells were notably higher in the patient group (Figure 5B,C). DEG analysis revealed upregulation of genes involved in B cell receptor signalling, B cell activation, and defence response in the pneumonia cohort (Figure S5C and Table S3).

Further analysis showed a marked prevalence and expansion of PB and PC.1 in the lung and BALF (Figures 5D–F and S5D,E). mIHC assays detected IgA^+^ Ki67^+^ plasma cells and diffuse IgA antibodies in the mucosal region and alveolar lumen (Figure 5G,H). Flow cytometry analysis also revealed high proportions of CD38^+^ CD27^+^ cells among CD19^+^ cells in the BALF of COVID‐19 patients (Figures 5I and S5F).

Examining the PB and plasma cell clusters, we found that PB cells were predominantly of the IgG isotype, whereas PC.1 was enriched for IgA isotypes (Figure 5J). PB and PC.1, which are developmentally linked, exhibited overlap in BCR repertoire (Figure S5G) and high expression of the chemokine receptor gene *CXCR3* and the resident marker ITGAE (Figure 5K). Pairwise STARTRAC‐migration analysis indicated substantial BCR sharing between the lung and BALF for PB and PC.1 (Figure 5L). These findings suggest that plasmablasts and IgA^+^ plasma cells, particularly those with high CXCR3 expression, are activated and capable of migrating to the airways (alveoli) to enhance mucosal immunity.

### 3.6 The interaction of pro‐inflammatory monocyte‐derived macrophages and other activated immune cells

**FIGURE 6:**
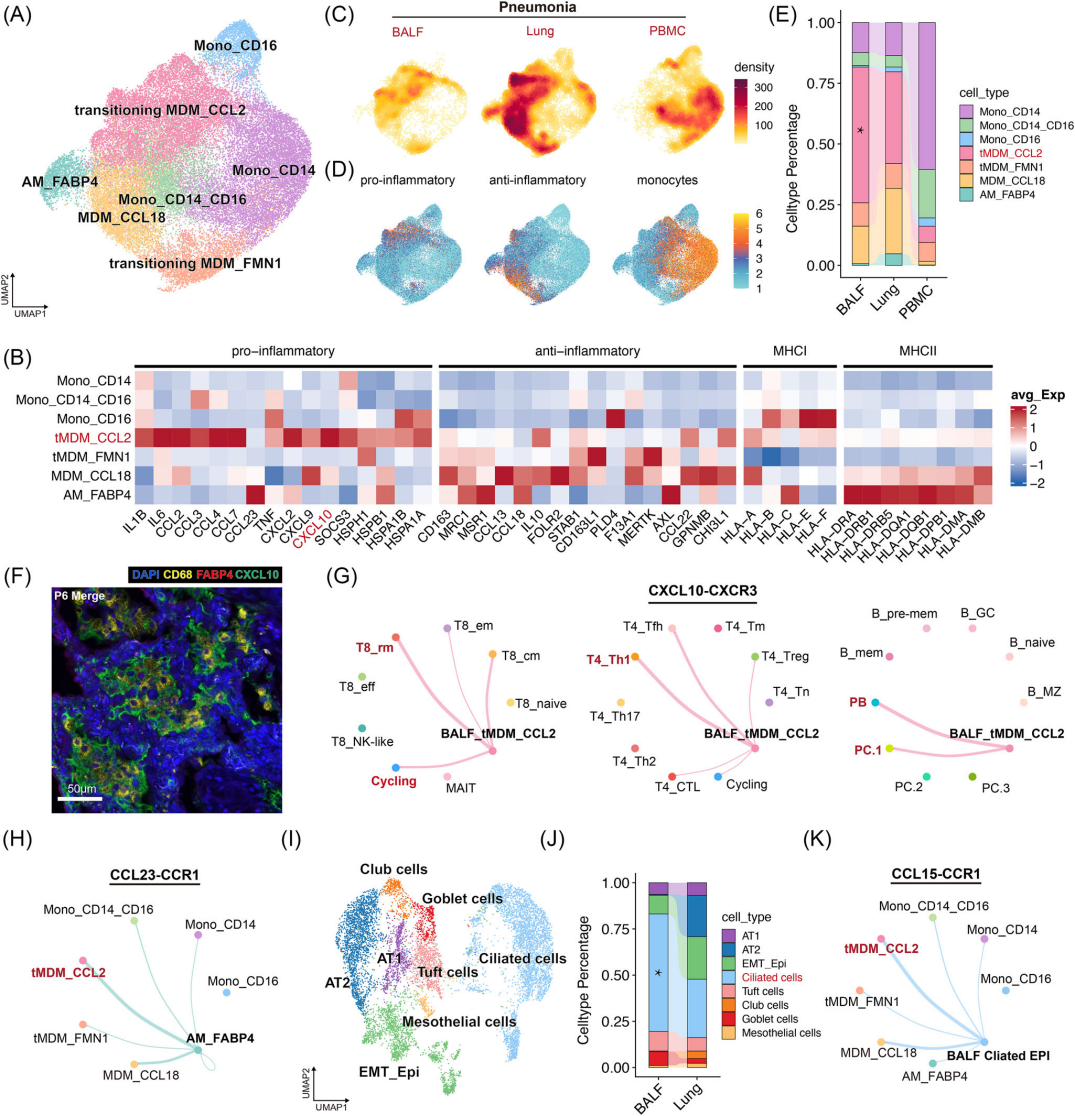
Pro‐inflammatory MDMs recruit activated immune cell into airway (alveolar) lumen. UMAP of 37 928 monocytes and macrophages. (B) Gene expression among indicated monocyte and macrophage subclusters. Genes are divided into 4 groups of pro‐inflammatory, anti‐inflammatory, MHC‐I and MHC‐II. (C) Density distribution of monocytes and macrophages in pneumonia group. (D) Projecting of set of gene signatures and marker genes on the UMAP embedding of (A), identifying different cell states. Gene sets of pro‐inflammatory and anti‐inflammatory are identical with those in (B). Monocytes marker gene include LYZ, S100A8, CD14, VCAN, FCN1 and S100A9. Each cell is coloured based on the average normalized expression of gene sets. (E) Relative proportion of each monocyte and macrophage subclusters in pneumonia group among sample types. (F) Abundant CXCL10^+^ CD68^+^ FABP4^−^ macrophages accumulated in the alveolar lumen of P6 were detected by mIHC. (G) Inferred cell‐cell interactions in CXCL signalling pathway of tMDM_CCL2 in BALF with subclusters of CD8^+^ T cells, CD4^+^ T cells, and B and plasma cells. (H) Inferred CCL23‐CCR1 signalling interaction strength of AM_FABP4 with other subclusters of monocytes and macrophages. (I) UMAP of epithelial cells, and cell type annotation of major subclusters. (J) Epithelial subcluster distributions in lung and BALF of pneumonia group. (K) Inferred CCL15‐CCR1 signalling interaction strength of ciliated cells in BALF with subclusters of monocytes and macrophages.

We next focussed on mono‐macrophages, which were significantly increased in the lungs of patients with severe pneumonia. We analysed a total of 37 928 myeloid cells and identified seven distinct phenotypes (Figures 6A and S6A; Table S2), including three clusters of monocytes, three clusters of MDMs (MAFB, MAF, CSF1R and SPP1), and one cluster of alveolar macrophages (FABP4). Among these, a specific MDM cluster, termed transitioning MDM_CCL2 (tMDM_CCL2), was notably expanded in the pneumonia group compared to controls (Figure S6B). This cluster expressed a broad range of chemokines, including CCL2 and CXCL10, and various heat shock proteins (Figure 6B).

Given the high levels of CXCL10 expressed by tMDM_CCL2, along with the expression of its receptor CXCR3 in other activated immune cells, we hypothesized that these cells may interact with other activated immune cells via CXCL10‐CXCR3 axis (Figure 6C–E).

The mIHC images of P6 revealed numerous CD68^+^ macrophages in the alveolar lumen, surrounded by a high concentration of CXCL10 (Figure 6F). CellChat analysis revealed significant interactions between tMDM_CCL2 and other immune cell clusters, such as T8_rms, T4_Th1, PC.1 and PBs, via the CXCL10‐CXCR3 axis (Figures 6G and S6C–E). This suggests that pro‐inflammatory MDMs in the airway and alveolar lumen may recruit and activate CXCR3‐expressing immune cells.

We next analysed the interaction of tMDM_CCL2 cells and other myeloid clusters (Figure S6F). We found that the alveolar macrophages (AM_FABP4) interacted with tMDM_CCL2 via the CCL23‐CCR1 axis (Figure 6H). This might suggest that alveolar macrophages might trigger the early immune response by recruiting tMDM_CCL2 to infection sites.

We also identified a group of ciliated cells on the airway's inner surface, potentially serving as a physical barrier against pathogens (Figures 6I and S6G;Table S2). These cells were predominantly found in BALF and expressed high levels of CCL15 (Figure 6J). CellChat analysis suggested a CCL15‐CCR1 axis between ciliated cells and tMDM_CCL2 (Figures 6K and S6H), indicating that ciliated cells could interact with tMDM_CCL2 to enhance the immune response during pathogen challenge. Additionally, interactions between ciliated cells and T/B lineage cells showed stronger promigratory signalling (CCL28/CCR10, CCL15/CCR1, CXCL16/CXCR6) in T8_rms, cycling, and plasma cells (Figure S6I–L).

In summary, our findings suggest that pro‐inflammatory MDMs play a crucial role during acute pulmonary inflammation. They can be recruited by resident cell subsets, such as ciliated cells and alveolar macrophages, and also interact and activate other immune cells within lung interstitia the airways (alveoli).

## 4 DISCUSSION

This study elucidates the cross‐compartment sharing patterns of activated immune cells—specifically T8_rms, plasma cells, and pro‐inflammatory MDMs—between the lung interstitium and the airways in severe pneumonia. Our findings reveal the interaction of pro‐inflammatory MDMs with other activated immune cells via the CXCL10‐CXCR3 axis, offering novel insights into lung immune cell distribution with significant implications for clinical practice and translational research.

The accumulation of highly active immune cells in the lungs during severe pneumonia mirrors findings from studies on COVID‐19 and other pathogen‐induced pneumonias.^2^, ^9^, ^15^, ^16^, ^32^ Due to the challenges in obtaining lung tissue samples from severely ill patients, some studies on pulmonary pathogens and immune responses have relied on autopsy data. As an alternative, previous research has used BALF and PBMCs to assess lung inflammation.^4^, ^8^, ^9^, ^15^, ^18^, ^19^ However, while BALF and peripheral blood contain distinct immune cell populations, it remains unclear which sample better reflects the immune cell populations in the lungs. Additionally, although early findings suggest that immune cells from the blood can migrate to the lungs and even the airways,^19^, ^23^, ^33^ their precise phenotypic transformations and sharing patterns have not been thoroughly investigated.

By employing a novel biopsy technique for critically ill patients, we can now simultaneously obtain lung tissue, BALF, and peripheral blood from those with severe pneumonia.^29^ Leveraging TCR data from CD8^+^ T cells in patients, especially with viral infections, we categorize these patterns into three distinct groups, providing new insights into their behaviours. Notably, other immune cell subtypes exhibit similar patterns.

Since we did not obtain the BALF samples from the control group, it was difficult to directly assess the distribution characteristics of these three cell‐sharing patterns in the control group. However, using the single R package, we indirectly drew the following conclusions. The first group (Pattern 1a) includes highly active TRMs, plasma cells and proinflammatory macrophages, distributed across the lung interstitium and airways. These cells, associated with ongoing infections, were also observed in BALF from COVID‐19 patients.^16^, ^17^, ^19^, ^21^, ^24^ The second group (Pattern 1b) consists of quiescent immune cells within the lung parenchyma, such as resting T8_rms and alveolar macrophages, likely non‐proliferative and unrelated to the current infection.^10^, ^16^, ^17^, ^18^, ^24^, ^25^, ^26^, ^34^, ^35^ Previous studies have reported the presence of T8_rms, tissue‐resident memory B cells, and resident alveolar macrophages in the lung interstitium, which can rapidly reactivate upon infection. The third group (Pattern 2) comprises cells that migrate from the bloodstream to the lungs, characterized by proliferation and expansion in the blood but with limited involvement in pulmonary infection. While these cells are present in lung tissue, they rarely appear in the airways.

Through the analysis of a representative T‐cell clone, we discovered that some active TRMs likely originate from some antigen‐responded CX3CR1^+^ T_effs in the bloodstream, upregulating CXCR3 and ITGAE during their transition to lung tissue.^23^, ^25^ Consistent with previous studies,^36^, ^37^, ^38^ the CXCR3 expression is crucial for adaptive immune cells migration into the airway lumen. In the influenza model of mice, specific T8_rms have been proven rapidly expand upon encountering antigens in the lungs, indicating a TCR‐specific response to infection.^33^, ^39^, ^40^ As respiratory pathogens most are distributed in the airways, these immune cells have limited antigen exposure in the bloodstream,^41^ resulting in minimal expansion and making it challenging to detect TCR abundance through single‐cell sequencing. However, once they reach the lungs and airways, they rapidly proliferate in response to antigen stimulation. Since most terminally‐differentiated T_effs tended to be irrelevance to a certain type of active viral infections and lacked the transitional capability to upregulate CXCR3 during infiltration, they were unlikely to further migrate to the airway (alveolar lumen) during pneumonia and mainly distributed across the lung and peripheral blood.^17^, ^19^, ^31^, ^33^ Similarly, we identified a cluster of activated plasma cells enriched in both the lung interstitium and airway (alveolar) lumen. In line with recent findings,^26^, ^27^, ^34^, ^35^ this group of plasma cells expressed high levels of chemokine receptors (e.g. CXCR3), resident markers (e.g., ITGAE), and mainly comprised IgA‐secreting cells. Besides, it has been documented in mouse models that the expression of CXCR3 appeared to endow these cells with the capacity to migrate into the airways and alveoli during lung infection.^26^

Tracking MDM sharing patterns poses challenges due to the lack of specific markers. Nevertheless, we identified both pro‐inflammatory and anti‐inflammatory macrophages distributed throughout the lungs and airways, consistent with activated T8_rms. As key players in innate immunity, macrophages drive early inflammation, promoting the chemotaxis of other immune cells to the lungs and airways.^2^, ^10^, ^11^, ^21^, ^42^ Our findings reinforce the crucial role of macrophages in amplifying immune responses, offering potential therapeutic targets for pneumonia treatment. Thus, understanding these patterns is vital for advancing both clinical management and translational research. The cross‐compartment sharing of active immune cells between the lung parenchyma and the airways render BALF a reliable indicator of lung inflammation.

Notably, this body of evidence can only serve as mechanistic clues or a working hypothesis, because the evidence we currently have is merely the observed phenomena or phenotypes in clinical specimens, and has not yet been verified through relevant basic experiments. This is precisely the focus of our subsequent research, such as targeting the CCL15/23‐CCR1 axis may inhibit early inflammatory amplification, while modulating the CXCL10‐CXCR3 axis could attenuate specific immune responses.^43^, ^44^ Moreover, employing monoclonal antibodies to selectively target pro‐inflammatory or anti‐inflammatory macrophages, based on cell surface antigens, could allow precise modulation of immune responses.

A key limitation of the present study is the small and aetiologically heterogeneous patient cohort, which prevents formal statistical comparison of pattern prevalence across pathogen categories. Immune responses can vary significantly depending on the pathogen, and our analyses focussed on identifying common patterns across major immune cell subtypes. While this diversity broadens the research's appeal, future studies should focus on specific pathogens to comprehensively characterize the functionalities and patterns of distinct immune cell subtypes. In addition, the modest sample size limits the generalizability of our single‐cell data findings and the use of subject‐level statistics, necessitating further validation through larger‐scale clinical cohorts and basic experiments, including protein level and quantitative spatial validation. Third, the absence of BALF samples from control subjects leads to lack of direct evidence to TCR pattern definition, despite the use of complementary computational approaches. Future studies incorporating control BALF samples, where ethically feasible, will be essential to strengthen and validate these findings. Finally, lack of vascular perfusion in lung biopsy processing means that intravascular immune cells cannot be physically excluded from the tissue fraction. The distinction between true tissue‐resident and tissue‐associated or intravascular cells therefore relies on transcriptional criteria alone, which may not capture all intravascular contamination.

## 5 CONCLUSIONS

This study highlights the critical role of immune cell cross‐compartment sharing patterns in lung protection and inflammation. Our research also suggests BALF serve as a surrogate window into parenchymal inflammation. These insights have profound implications for the assessment and monitoring of severe pneumonia.

## AUTHOR CONTRIBUTIONS

Ye Wang, Xuyu Cai, and Weiya Wang designed this study. Yuean Zhao and Linjing Gong performed data analysis. Ye Wang, Xuyu Cai, and Weiya Wang contributed to the experimental design. Yuean Zhao, Sifan Zhang and Guanglei Yang performed the experiments. Sifan Zhang and Chaoyang Wang provided experimental methods. He Yu and Ye Wang provided clinical samples. Linjing Gong, Yuean Zhao, He Yu, Xuyu Cai and Ye Wang wrote the manuscript, with all authors contributing to providing feedback. Yuean Zhao, Linjing Gong and He Yu were designated co‐first authors because their contributions were equally essential to this project. Yuean Zhao was listed first for the majority computational analysis, which were significant for this study's conclusions. Linjing Gong was listed second for her major contribution in writing the manuscript.

## CONFLICT OF INTEREST STATEMENT

The authors declare no conflicts of interest.

## ETHICS STATEMENT

All procedures relating patient samples were in line with the ethical standards of the IRB and the Helsinki Declaration. Prior to sample collection, written informed consents were obtained from all participants or, where applicable, from their legal guardians in accordance with standard procedures. Ethical approval was obtained from the Institutional Review Board of West China Hospital of Sichuan University (approval number: 2021‐1250, 2021‐1650, 2022‐1395 and 2022‐1401).

## Supporting information



Supporting Information

## Data Availability

The original scRNA‐seq and scTCR/BCR data reported in this paper have been deposited in the Genome Sequence Archive^45^ in National Genomics Data Center,^46^ China National Center for Bioinformation / Beijing Institute of Genomics, Chinese Academy of Sciences that are publicly accessible at https://ngdc.cncb.ac.cn/gsa, under the accession numbers HRA005961. R and Python code used for the analysis and visualization of scRNA‐seq data has been deposited on GitHub: https://github.com/YeWangWCH/Activated‐immune‐cells‐migration‐pattern‐in‐severe‐pneumonia.

## References

[1] Cillóniz C , Torres A , Niederman MS. Management of pneumonia in critically ill patients. BMJ 2021;375:e65871. 10.1136/bmj-2021-065871 34872910

[2] Bos L , Ware LB . Acute respiratory distress syndrome: causes, pathophysiology, and phenotypes. Lancet 2022;400(10358):1145–1156. 36070787 10.1016/S0140-6736(22)01485-4

[3] Dunbar PR , Cartwright EK , Wein AN , et al. Pulmonary monocytes interact with effector T cells in the lung tissue to drive TRM differentiation following viral infection. Mucosal Immunol 2020;13(1):161–171. 31723250 10.1038/s41385-019-0224-7 PMC6917844

[4] Li Y , Schneider AM , Mehta A , et al. SARS‐CoV‐2 viremia is associated with distinct proteomic pathways and predicts COVID‐19 outcomes. J Clin Invest 2021;131(13):e148635. 34196300 10.1172/JCI148635 PMC8245177

[5] Pormohammad A , Ghorbani S , Khatami A , et al. Comparison of influenza type A and B with COVID‐19: a global systematic review and meta‐analysis on clinical, laboratory and radiographic findings. Rev Med Virol 2021;31(3):e2179. 33035373 10.1002/rmv.2179 PMC7646051

[6] Synowiec A , Szczepanski A , Barreto‐Duran E , et al. Severe acute respiratory syndrome coronavirus 2 (SARS‐CoV‐2): a systemic infection. Clin Microbiol Rev 2021;34(2):e00133‐20. 33441314 10.1128/CMR.00133-20 PMC7849242

[7] Wang H , Paulson KR , Pease SA , et al. Estimating excess mortality due to the COVID‐19 pandemic: a systematic analysis of COVID‐19‐related mortality, 2020–21. Lancet 2022;399(10334):1513–1536. 35279232 10.1016/S0140-6736(21)02796-3 PMC8912932

[8] Kreutmair S , Unger S , Nunez NG , et al. Distinct immunological signatures discriminate severe COVID‐19 from non‐SARS‐CoV‐2‐driven critical pneumonia. Immunity 2021;54(7):1578–1593. 34051147 10.1016/j.immuni.2021.05.002 PMC8106882

[9] Ren X , Wen W , Fan X , et al. COVID‐19 immune features revealed by a large‐scale single‐cell transcriptome atlas. Cell 2021;184(7):1895–1913. 33657410 10.1016/j.cell.2021.01.053 PMC7857060

[10] Aegerter H , Lambrecht BN , Jakubzick CV , Biology of lung macrophages in health and disease. Immunity 2022;55(9):1564–1580. 36103853 10.1016/j.immuni.2022.08.010 PMC9533769

[11] Madissoon E , Oliver AJ , Kleshchevnikov V , et al. A spatially resolved atlas of the human lung characterizes a gland‐associated immune niche. Nat Genet 2023;55(1):66–77. 36543915 10.1038/s41588-022-01243-4 PMC9839452

[12] Qin L , Wang W , Liu H , et al. CD4+ and CD8+ T lymphocytes in lung tissue of NSIP: correlation with T lymphocytes in BALF. Resp Med 2013;107(1):120–127. 10.1016/j.rmed.2012.09.021 23085212

[13] Quinton LJ , Walkey AJ , Mizgerd JP . Integrative physiology of pneumonia. Physiol Rev 2018;98(3):1417–1464. 29767563 10.1152/physrev.00032.2017 PMC6088146

[14] Tschernig T , Fliegert F , Westermann J , et al. Increased expression of activation markers and adhesion molecules on lung T‐cells compared with blood in the normal rat. Eur Respir J 1999;13(1):66–70. 10836325 10.1183/09031936.99.13106699

[15] Grant RA , Morales‐Nebreda L , Markov NS , et al. Circuits between infected macrophages and T cells in SARS‐CoV‐2 pneumonia. Nature (London) 2021;590(7847):635–641. 33429418 10.1038/s41586-020-03148-w PMC7987233

[16] Liao M , Liu Y , Yuan J , et al. Single‐cell landscape of bronchoalveolar immune cells in patients with COVID‐19. Nat Med 2020;26(6):842–844. 32398875 10.1038/s41591-020-0901-9

[17] Szabo PA , Dogra P , Gray JI , et al. Longitudinal profiling of respiratory and systemic immune responses reveals myeloid cell‐driven lung inflammation in severe COVID‐19. Immunity 2021;54(4):797–814. 33765436 10.1016/j.immuni.2021.03.005 PMC7951561

[18] Wauters E , Van Mol P , Garg AD , et al. Discriminating mild from critical COVID‐19 by innate and adaptive immune single‐cell profiling of bronchoalveolar lavages. Cell Res 2021;31(3):272–290. 33473155 10.1038/s41422-020-00455-9 PMC8027624

[19] Xu G , Qi F , Li H , et al. The differential immune responses to COVID‐19 in peripheral and lung revealed by single‐cell RNA sequencing. Cell Discov 2020;6(1):73. 33101705 10.1038/s41421-020-00225-2 PMC7574992

[20] Delorey TM , Ziegler CGK , Heimberg G , et al. COVID‐19 tissue atlases reveal SARS‐CoV‐2 pathology and cellular targets. Nature 2021;595(7865):107–113. 33915569 10.1038/s41586-021-03570-8 PMC8919505

[21] Melms JC , Biermann J , Huang H , et al. A molecular single‐cell lung atlas of lethal COVID‐19. Nature 2021;595(7865):114–119. 33915568 10.1038/s41586-021-03569-1 PMC8814825

[22] Rendeiro AF , Ravichandran H , Bram Y , et al. The spatial landscape of lung pathology during COVID‐19 progression. Nature 2021;593(7860):564–569. 33780969 10.1038/s41586-021-03475-6 PMC8204801

[23] Snyder ME , Finlayson MO , Connors TJ , et al. Generation and persistence of human tissue‐resident memory T cells in lung transplantation. Sci Immunol 2019;4(33):eaav5581. 30850393 10.1126/sciimmunol.aav5581 PMC6435356

[24] Carbone FR . Unique properties of tissue‐resident memory T cells in the lungs: implications for COVID‐19 and other respiratory diseases. Nat Rev Immunol 2023;23(5):329–335. 36494455 10.1038/s41577-022-00815-z PMC9735123

[25] Desai P , Tahiliani V , Stanfield J , et al. Inflammatory monocytes contribute to the persistence of CXCR3hi CX3CR1lo circulating and lung‐resident memory CD8+ T cells following respiratory virus infection. Immunol Cell Biol 2018;96(4):370–378. 29363162 10.1111/imcb.12006 PMC5916332

[26] Oh JE , Song E , Moriyama M , et al. Intranasal priming induces local lung‐resident B cell populations that secrete protective mucosal antiviral IgA. Sci Immunol 2021;6(66):eabj5129. 34890255 10.1126/sciimmunol.abj5129 PMC8762609

[27] Wellford SA , Moseman AP , Dao K , et al. Mucosal plasma cells are required to protect the upper airway and brain from infection. Immunity 2022;55(11):2118–2134. 36137543 10.1016/j.immuni.2022.08.017 PMC9649878

[28] Metlay JP , Waterer GW , Long AC , et al. Diagnosis and treatment of adults with community‐acquired pneumonia. An official clinical practice guideline of the American Thoracic Society and Infectious Diseases Society of America. Am J Resp Crit Care 2019;200(7):e45–e67. 10.1164/rccm.201908-1581ST PMC6812437 31573350

[29] Zhao Y , Jiang F , Yu H , et al. Bronchus‐blocked ultrasound‐guided percutaneous transthoracic needle biopsy (BUS‐PTNB) for intubated patients with severe lung diseases. Crit Care (London, England) 2021;25(1):359. 10.1186/s13054-021-03782-4 PMC8515780 34649600

[30] Gerlach C , Moseman EA , Loughhead SM , et al. The chemokine receptor CX3CR1 defines three antigen‐experienced CD8 T cell subsets with distinct roles in immune surveillance and homeostasis. Immunity 2016;45(6):1270–1284. 27939671 10.1016/j.immuni.2016.10.018 PMC5177508

[31] Herndler‐Brandstetter D , Ishigame H , Shinnakasu R , et al. KLRG1+ effector CD8+ T cells lose KLRG1, differentiate into all memory T cell lineages, and convey enhanced protective immunity. Immunity 2018;48(4):716–729. 29625895 10.1016/j.immuni.2018.03.015 PMC6465538

[32] Heung LJ , Wiesner DL , Wang K , et al. Immunity to fungi in the lung. Semin Immunol 2023;66:101728. 36841146 10.1016/j.smim.2023.101728 PMC10148604

[33] Slütter B , Van Braeckel‐Budimir N , Abboud G , et al. Dynamics of influenza‐induced lung‐resident memory T cells underlie waning heterosubtypic immunity. Sci Immunol 2017;2(7):eaag2031. 28783666 10.1126/sciimmunol.aag2031 PMC5590757

[34] Barker KA , Etesami NS , Shenoy AT , et al. Lung‐resident memory B cells protect against bacterial pneumonia. J Clin Invest 2021;131(11):e141810. 34060477 10.1172/JCI141810 PMC8159694

[35] Tan H , Juno JA , Esterbauer R , et al. Lung‐resident memory B cells established after pulmonary influenza infection display distinct transcriptional and phenotypic profiles. Sci Immunol 2022;7(67):eabf5314. 35089815 10.1126/sciimmunol.abf5314

[36] Abboud G , Desai P , Dastmalchi F , et al. Tissue‐specific programming of memory CD8 T cell subsets impacts protection against lethal respiratory virus infection. J Exp Med 2016;213(13):2897–2911. 27879287 10.1084/jem.20160167 PMC5154936

[37] Hikono H , Kohlmeier JE , Takamura S , et al. Activation phenotype, rather than central‐ or effector‐memory phenotype, predicts the recall efficacy of memory CD8+ T cells. J Exp Med 2007;204(7):1625–1636. 17606632 10.1084/jem.20070322 PMC2118640

[38] Ozga AJ , Chow MT , Lopes ME , et al. CXCL10 chemokine regulates heterogeneity of the CD8+ T cell response and viral set point during chronic infection. Immunity 2022;55(1):82–97. 34847356 10.1016/j.immuni.2021.11.002 PMC8755631

[39] Hu JK , Kagari T , Clingan JM , et al. Expression of chemokine receptor CXCR3 on T cells affects the balance between effector and memory CD8 T‐cell generation. Proc Natl Acad Sci U S A . 2011;108(21):E118–E127. 21518913 10.1073/pnas.1101881108 PMC3102421

[40] Mueller SN , Mackay LK . Tissue‐resident memory T cells: local specialists in immune defence. Nat Rev Immunol 2016;16(2):79–89. 26688350 10.1038/nri.2015.3

[41] Kim N , Kim HK , Lee K , et al. Single‐cell RNA sequencing demonstrates the molecular and cellular reprogramming of metastatic lung adenocarcinoma. Nat Commun 2020;11(1):2285. 32385277 10.1038/s41467-020-16164-1 PMC7210975

[42] Morrell ED , Bhatraju PK , Mikacenic CR , et al. Alveolar macrophage transcriptional programs are associated with outcomes in acute respiratory distress syndrome. Am J Resp Crit Care 2019;200(6):732–741. 10.1164/rccm.201807-1381OC PMC6775881 30990758

[43] Ding W , Li R , Song T , et al. AMG487 alleviates influenza A (H1N1) virus‐induced pulmonary inflammation through decreasing IFN‐gamma‐producing lymphocytes and IFN‐gamma concentrations. Br J Pharmacol 2024;181(13):2053–2069. 38500396 10.1111/bph.16343

[44] Guo K , Yombo D , Wang Z , et al. The chemokine receptor CXCR3 promotes CD8(+) T cell‐dependent lung pathology during influenza pathogenesis. Sci Adv 2024;10(1):eadj1120. 38170765 10.1126/sciadv.adj1120 PMC10776024

[45] Chen T , Chen X , Zhang S , et al. The genome sequence archive family: toward explosive data growth and diverse data types. Genomics Proteomics Bioinformatics 2021;19(4):578–583. 34400360 10.1016/j.gpb.2021.08.001 PMC9039563

[46] Cncb‐Ngdc MAP . Database resources of the National Genomics Data Center, China National Center for Bioinformation in 2025. Nucleic Acid Res 2025;53(D1):D30–D44. 39530327 10.1093/nar/gkae978 PMC11701749

