# Supplementary material for: Activated immune cells present a lung interstitial‐to‐airway/alveolar cross‐compartment sharing pattern in severe pneumonia
Source: Clin Transl Med. 2026 May 21;16(5):e70696. doi: 10.1002/ctm2.70696 (PMC13240578; doi:10.1002/ctm2.70696)
Supplement: Supplementary file 1 — Supporting Information [file CTM2-16-e70696-s001.pdf]

Supplementary Materials for

Activated immune cells present a lung interstitial-to-airway/alveolar cross-compartment sharing pattern in severe pneumonia

Yuean Zhao[1,6], Linjing Gong[1,2,6], He Yu[1,2,6], Sifan Zhang[1,2], Guanglei Yang[3], Chaoyang Wang[4], Weiya Wang[5]*, Xuyu Cai[3]*, Ye Wang[1,2]*

Correspondence to: wangye@wchscu.cn

This PDF file includes:

Materials and Methods
Figures. S1 to S6
Tables S1 to S7

Materials and Methods

RESOURCE AVAILABLITY

The original scRNA-seq and scTCR/BCR data reported in this paper have been deposited in the Genome Sequence Archive (Genomics, Proteomics & Bioinformatics 2021) in National Genomics Data Center (Nucleic Acids Res 2025), China National Center for Bioinformation / Beijing Institute of Genomics, Chinese Academy of Sciences that are publicly accessible at https://ngdc.cncb.ac.cn/gsa, under the accession numbers HRA005961. R and Python code used for the analysis and visualization of scRNA-seq data has been deposited on GitHub: https://github.com/YeWangWCH/Activated-immune-cells-migration-pattern-in-severe-pneumonia.

EXPEREIMENTAL MODEL AND SUBJECT DETAILS

Ethic statement

All procedures relating patient samples were in line with the ethical standards of the IRB and the Helsinki Declaration. Prior to sample collection, written informed consents were obtained from every participant in accordance with standard procedures. Ethical approval was obtained from the Institutional Review Board of West China Hospital of Sichuan University (approval number: 2021-1250, 2021-1650, 2022-1395 and 2022-1401).

Biological samples

This study was conducted along with a clinical trial designed to evaluate the safety and efficacy of a novel technique for obtaining lung biopsy specimens in severe pneumonia. The sample size was determined using a single-arm objective performance criteria approach based on the acceptable rate of severe complications, according to the following formula:

$$n = \frac{[Z_{1-\alpha}\sqrt{P_0(1-P_0)} + Z_{1-\beta}\sqrt{P_1(1-P_1)}]^2}{(P_1-P_0)^2}$$

According to previous reports, 73.0%–76.9% of pathological results provide strong clinical significance, and 84.0%–95.3% contribute important information for etiological diagnosis. After balancing these expected benefit rates, we assumed an acceptable severe complication rate of 15% for sample size estimation. Based on this assumption, 22 patients were required to achieve 80% statistical power at a two-sided alpha level of 2.5%.

Among the 22 patients enrolled in the clinical trial, individuals without a diagnosis of severe pneumonia (e.g., patients with lung cancer or lymphoma) were excluded, as were those without adequate specimens for single-cell RNA sequencing. Ultimately, 12 patients with severe pneumonia were included in the present study.

Matched lung tissues, BALF and peripheral blood from 12 patients with severe pneumonia, as well as paired lung tissues and peripheral blood from 5 donors with suspected lung cancer were collected at West China Hospital of Sichuan University from December 2021 to May 2022. Severe pneumonia was defined according to the consensus guidelines of Infectious Diseases Society of America/American Thoracic Society.(1) Patients admitted to the medical intensive care unit (MICU) were under mechanical ventilation, and their conditions were thoroughly evaluated by at least two respiratory physicians. Lung biopsy specimens were obtained following the BUS-PTNB workflow, which was published in our previous study. Controls were recruited among donors with suspected lung cancer and distal normal lung tissues were obtained during surgery. None of them underwent neoadjuvant therapy before surgery.

Clinical data capture

Clinical data needed for this study was retrospectively collected from the hospital information system. Patients' sex, age, pre-existing diseases, hospital length of stay, and outcome were collected. APACHEII and SOFA score were assessed on the day of admission to MICU. Laboratory tests results and pathogen was collected. Any disagreement was resolved by team discussion until consensus reached.

METHOD DETAILS

Sample processing

Lung biopsy, BALF and peripheral blood sample were collected at the same day of BUS-PTNB, and their processing was within 3 hours of sampling. All samples were initially stored at -80°C and thawed in batches for single cell RNA library construction to minimize the risk of batch effects.

Blood sample processing

Blood sample was collected in a 6 mL serum separation tube and two 10 mL EDTA anti-coagulation tubes. Blood samples in serum separation tube were centrifuged (1600 g, 10 min, 4 °C) to collect serum, while those in EDTA anti-coagulation tube were to collect plasma. Both serum and plasma were stored as -80 °C for later use.

Ca/Mg-free PBS of removed plasma's volume was added to remaining blood cells to maintain the density of whole blood. Ficoll-Paque medium was used for density gradient centrifugation (800 g, 20 min, AC1, DE1, 20 °C). Peripheral blood mononuclear cells (PBMC) were collected, re-suspended in cooled PBS and then centrifuged at 500 g for 5 mins. Red blood cells were removed using Red Blood Cell Lysis Buffer if macroscopic. Finally, cells were cryopreserved using Serum/Protein-Free Cell Freezing Medium and were stored at -80 °C.

Lung biopsy sample processing

Lung biopsy sample were preserved in a 1.5 mL centrifuge tube containing 1 mL cooled 10%FBS and 90% RPMI Medium. Vascular perfusion to deplete intravascular immune cells was not performed. Lung samples were firstly transferred into a 1.5 mL centrifuge tube containing 200 uL 2%FBS and 98% RPMI Medium. Samples were cut into tiny pieces with the diameter of about 1 mm. A cocktail of digestion enzymes (Table E7) was applied to the working media with final volume of 1 mL. Samples were incubated at 37 °C for 10 mins, and after a short centrifuge, the supernatant was transferred to a 15 mL centrifuge tube containing 4 mL cooled 2%FBS and 98% RPMI Medium to cease the digestion. The undigested precipitates were again pipetted into a second digestion media of 1 mL total volume (Table E7). The second incubation lasted for 20 mins at 37°C, after which the media was mixed with the former one, filtered through a 40um cell strainer, and was centrifuged for 5 mins at 500 g and 4 °C. Resuspended using 1 mL PBS with 0.04% BSA, the cells were centrifuged again and cryopreserved and stored in -80 °C with Serum/Protein-Free Cell Freezing Medium. Due to relatively minor amount of total cell count, cryopreservation instead of erythrocytes lysis was chosen to get rid of red blood cells.

BALF sample processing

Approximately 10 mL of BALF was acquired for each patient. BALF was first filtered through a 70 mm cell strainer to get rid of airway clumps and then centrifuged for 10 mins at 500 g and 4 °C. The supernatant was cryopreserved and stored at -80 °C. Cells were resuspended in 5 mL cooled RPMI Medium and then was passed through a 40mm cell strainer. The cells were centrifuged again and cryopreserved in -80 °C.

scRNA-seq library preparation and sequencing

Cyropreserved lung, BALF or PBMC cell suspensions were thawed at 37 °C and centrifuged at 500 g for 5 mins at 4 °C. Cells were resuspended in 100 µL PBS with 0.04% BSA. Dead Cell Removal Kit (Miltenyi Biotec, 130-090-101) was utilized for samples with a viability of less than 60% according to the manufacturer's instructions. A 40 µm cell strainer was applied to remove aggregates prior to barcoding. All samples were barcoded through 10x Chromium Single Cell platform, aiming for 10000 cells each reaction if cell count permitted, otherwise all cells were loaded. Single-cell RNA and VDJ libraries were conducted using Library Construction Kit, Chromium Single Cell Human TCR Amplification Kit and Chromium Single Cell Human BCR Amplification Kit following the manufacturer's instructions. A unique index from Dual Index Kit TT Set A was given to each sequencing library. With 15 to 30M of data estimated per cell, single-cell transcriptome library sequencing was performed utilizing the NovaSeq PE150.

Flow cytometry

For flow cytometry, 4 paired BALF and PBMC samples from patients with severe COVID-19 pneumonia were analyzed. Lung sample from a healthy donor was used as control. Cryopreserved samples from a same patient were thawed in 37 °C water bath, centrifuged at 500 g for 5 mins. Cell pellets were resuspended in 100 µL PBS. After adding 0.5 µL LIVE/DEAD Fixable Blue (Invitrogen, L34962A), samples were incubated for 15 mins from light at 4 °C, washed using 1 mL PBS and centrifuged at 500 g for 5 mins. Cells were reconstituted in 50 µL Fc block buffer (1 µL human HAB serum and 49 µL PBS) and incubated for 10 mins from light at 4 °C. Cells were stained using 50 µL of surface staining antibody cocktail (Table E5). Samples were incubated for 30 mins from light at 4 °C, washed using 1 mL PBS and centrifuged at 500 g for 5 mins. Cells were fixed and permeabilized using Transcription Factor Buffer Set according to manufacturer's instruction. Cells were fully stained using 100 µL of intracellular antibody cocktail (Table E5) and incubate for 20-30 minutes from light at RT. After centrifuging, cells were resuspended in 300 µL IC Fixation buffer (Invitrogen, 00-8222-49) and stored at 4°C for up to 18 hours before acquired at BD FACSymphony A5 CellAnalyzer. Compensation samples was conducted using Anti-mouse Ig beads, Anti-rat Ig beads and UltraComp eBeads. The results were analyzed by Flowjo.

Multiplex fluorescence immunohistochemistry

Multi-color IHC was conducted using the Opal 7-Color Manual IHC Kit (Akoya Biosciences NEL811001KT) according to manufacturer's protocol. Antibodies used in each panel and their concentration were presented in Table E6. Stained slides were visualized using the PhenoImager HT (Akoya Biosciences) with a dry 20/0.75 lens and Vectra Quantitative Pathology Imaging Systems (Akoya Biosciences). Analyses were conducted

using Phenochart (v1.1.0). Regions of interest (ROIs) were selected from each section based on overall staining quality and representativeness of the tissue architecture. Given the limited size of biopsy specimens, ROIs were chosen to best reflect the predominant staining patterns observed across the entire section while avoiding areas with artifacts or poor signal.

mNGS of lung and BALF

Another fresh specimen of lung biopsy and BALF were obtained at the same time of sampling. DNA extraction and library construction was performed using QubitTM dsDNA HS Assay Kit (Cat# Q32854, Thermo Fisher Scientific Inc, USA), the Total DNA library Preparation Kit (Cat. MD001T, MatriDx Biotech Crop., Hangzhou), and the Nucletic Acid Extraction Kit (Cat. MD013, MatriDx Biotech Crop., Hangzhou) following the manufacturer's insctruction. Sequenced data were compared with the reference genomes in the Genbank of the National Center for Biotechnology Information (NCBI) to identify the potential infection.

QUANTIFICATION AND STATISCAL ANALYSIS

Cellranger

The Cell Ranger (version 7.0.0, 10x Genomics) count pipeline was used to align FASTQ sequencing reads to the GRCh38 human genome assembly and quantify transcript. Notably, intronic reads were included by default in order to maximize sensitivity for whole transcriptome gene expression. Generated count and other outputs were used for further downstream analysis.

For TCR and BCR sequencing, sequence assembly and paired clonotype calling are carried out by the cellranger vdj pipeline using FASTQ files for V(D)J libraries. UMIs and cellular barcodes were used to build V(D)J transcripts per cell. Generated filtered contigs were used for further analysis.

Single-cell RNA-seq data analysis

Data quality control, normalization and integration

Genes expressed in fewer than 3 cells were discarded. Low quality cells were defined as mitochondrial gene counts ratio > 10% or number of genes < 200. Filtered matrices were normalized using 'NormalizeData' with normalization method as 'LogNormalize'. Then top 2,000 highly variable genes were found using the 'vst' method in 'FindVariableFeatures' function. The reciprocal PCA (RPCA) workflow was utilized for the integration of all individual datasets in order remove batch effects. The features were chosen using the 'SelectIntegrationFeatures' function by default and ranked by how many datasets they were found in. Using the top 50 dimensions. anchors were identified using 'FindIntegrationAnchors' function, with reduction set to 'rpca'. 12 samples out of 46 were specified as reference, 10 of which were pneumonia samples. And lung, BALF and PBMC took account for 5, 3 and 4 samples separately. The previously identified anchors were used to integrate all datasets together with 'IntegratedData' function (k.weight=80). The integrated data was scaled using 'ScaleData'. Considered as unwanted sources of variation, percentage of mitochondrial gene counts, percentage of erythrocytes gene counts and number of genes were regressed out. On the basis of the top 30 principal components, principal component analysis (PCA) and uniform manifold approximation and projection (UMAP) dimension reduction were carried out by 'RunPCA' and 'RunUMAP' function. The top 30 PCA reduction dimensions were used to build nearest-neighbour graphs, and clustering with a resolution of 0.5 was used in function 'FindNeighbors' and 'FindClusters'. Clusters whose cells had low amount of genes and simultaneous expression of multiple canonical cell type marker genes were defined as low-quality clusters and were removed, leaving 275,411 cells remained. 'RunUMAP' was applied again after removal to generate final UMAP reduction.

Cell type annotation

After the initial round of clustering, cell type annotation was manually accomplished by combining the analysis of differentially expressed genes (DEG) for each cluster determined by the 'FindAllMarkers' function (min.pct = 0.25, logfc.threshold = 0.25) with the visualization of canonical marker genes using the 'FeaturePlot' function. Finally, 18 cell types including NK cells, CD8+ T cells, CD4+ T cells, apoptotic T cells, cycling, B cells, plasma cells, megakaryocytes, neutrophils, CD14+ monocytes, macrophages, CD16+ monocytes, pDCs, cDCs, mast cells, fibroblasts, epithelial cells, and endothelial cells were identified. Top 10 marker genes sorted by log2 fold change in each cluster was deposited in Table E2. For comparison of cell type proportion between groups, these 18 cell types were classified into 10 major types (T cells, NK cells, B cells, plasma cells, myeloid cells, mast cells, DCs, megakaryocytes, cycling, parenchymal cells).

Subset analysis of T/NK cells, B cells, myeloid cells and epithelial cells

Second rounds of clustering on T/NK cells, B/plasma cells, myeloid cells, and epithelial cells separately was conducted to further elaborate analysis of these cell types, among which CD8+ T cells and CD4+ T cells

underwent another round of clustering. For T/NK cells, B/plasma cells and myeloid cells, datasets were split by individuals, while those with small amount of cell numbers were combined with other samples of different sample type from a same patient. The second round of clustering followed the same steps as the first. For epithelial cells, CD8+ T cells and CD4+ T cells, re-clustering started from 'RunPCA'. Same as first round clustering, clusters with multiple lineage markers or low gene count were considered as doublets or low quality cells and removed. Detailed procedure and parameter for re-clustering of each cell type was presented in code attached. Based on known markers or a comparison of the DEG with cell atlases, the resultant clusters were annotated to the different cell types. Calculated cell markers for each annotated subcluster were deposited in Table E2.

TCR sequence analysis

TCR clonetype classification and TCR ID nomenclature

After matching TCR chain information with each T cell by cellular barcode with R package 'scRepertoire'(2) (cloneCall = 'aa'), a few modifications of TCR clonetype was made, for TCR b chain (TRB) was considered as representative of the specificity and diversity of TCR clonetype rather than TCR a chain (TRA). Cells with multiple TRA or TRB and cells without TRB were removed. Namely, cells with exactly one TRA and one TRB chain and cells with only one TRB were remained for analysis. Each TCR was ranked across all samples from one patient by their frequency. TCR ID was named as the combination of patient ID and the ranking of the TCR. TCR clonetype expansion was classified into single (TRB frequency across patient = 1), small (1 < TRB frequency across patient <= 5), medium (5 < TRB frequency across patient <= 20), large (20 < TRB frequency across patient <= 100) and hyperexpanded (100 < TRB frequency across patient <= 2000). R package 'Startrac'(3) was used to calculate the transition and migration index of TCR across sample types.

Clustering of TCR abundance and definition of TCR pattern

As previously mentioned, T/NK cells underwent a third round clustering into CD8+ T cells and CD4+ T cells, among which TCR abundance was calculated. For each TCR ID, TCR abundance was defined as the proportion of the entire T cell repertoire of an individual sample occupied by a single clone. The ranking of a single TCR frequency in an individual sample was also counted. For pneumonia group, top 10 TCR ID of TCR frequency ranking in lung sample of patients with severe pneumonia were extracted. TCR abundance of these TCR ID in all three sample types was tallied into matrix, log transformed and finally hierarchically clustered and presented in heatmap with R package 'ComplexHeatmap' (ref) by default, resulting in three discrete TCR patterns in sample and cell type distribution. Lacking BALF sample, TCR pattern in control group was not directly calculated as in patient group, rather inferred by the relevance of gene expression within patterns across different disease state. Cells in patient group whose TCR ID were used in TCR abundance heatmap were included as the reference data, while those in control group with clonetype expansion being medium, large and hyperexpanded as the query data. TCR pattern of cells in the query data was defined by known TCR pattern's gene expression in patient reference data using R package SingleR(4) (label = TCR_pattern). Function 'plotScoreHeatmap' was used to evaluate singleR assignment scores across all cell-label combinations.

BCR sequence analysis

BCR sequence data analysis was carried out following the Immcantation workflow (http://immcantation.org/). V(D)J genes from filtered contigs in cellranger output were assigned by IgBLAST and converted to AIRR format using AssignGenes.py and MakeDb.py in Change-O.(5) Samples within same individual were pooled and each sample's original identity was added as prefix to cellular barcodes, in accordance with scRNA-seq data. Non-productive sequences were removed, as well as cells with multiple heavy chains or without heavy chains. By first identifying all of the gene segments that a person carries from V(D)J-rearrange sequences, V(D)J allele assignments were improved with 'findNovelAlleles' function R package TIgGER.(6) The genotype of an individual was then deduced from these sequences by 'inferGenotype' function, with which the initial V(D)J allele assignments can be corrected by 'reassignAlleles' function. The hamming distance threshold was found automatically by 'distToNearest' function (model = 'ham', normalize = 'len') and 'findThreshold ' function (method = 'gmm', model = 'gamma-norm') in R package SHazaM.(7) This threshold was then used to define clonal groups of a patient using 'hierarchicalClones' function of R package SCOPer,(8) 'cell-id' parameter set to 'barcode' to run in single-cell mode with paired chain sequences. Additionally, 'only_heavy' was set to FALSE and 'split-light' to TRUE, in order to include IGH plus IGK/IGL sequences and splitting clones by the light chain following heavy chain clustering. The isotype and clonal clustering information were transfer to B and plasma cells' metadata for subsequent analysis. BCR clonetype expansion was also divided into five categories: single (BCR clonal size = 1), small (1 < BCR clonal size <= 5), medium (5 < BCR clonal size <= 10), large (10 < BCR clonal size <= 100),

and hyperexpanded (BCR clonal size > 100). To determine the transition and migration index of BCR across sample types, the R package "Startrac"(3) was utilized.

Differential expression tests and GO enrichment

Differential expression tests were carried out contrasts including different groups of CD8+ TRMs and TCR abundances patterns. The 'FindMarkers' function in the R package Seurat(9) was used to identify DEGs using the 'MAST' approach.(5) R package EnhancedVolcano was used to visualize DEGs between different groups, with FC cutoff set to 1/-1. Genes with adjusted p value over 0.05 were considered significant and differentially expressed. Annotation database R package ‘‘org.Hs.eg.db’’ was used to map gene identifiers. ‘enrichGO’ function in R package clusterProfiler(10) was utilized for gene ontology and pathway enrichment analysis of previously discovered DEGs (pAdjustMethod = "BH", pvalueCutoff = 0.01, qvalueCutoff = 0.05). Annotation database R package ‘‘org.Hs.eg.db’’ was used to map gene identifiers and genes with avg_log2FC > 0.5 or < -0.5 were included. Only the consequence of biological process enrichment was presented. The results were sorted by qvalue and colored by gene counts enriched of a specific pathway. Results visualization as bar plots was realized using R package ‘ggplot2’.

Module scores for feature expression

Scores for gene expression programs in macrophages and monocytes were computed with canonical genes using the Seurat function AddModuleScore by default. Genes used were deposited in Table E2. Visualization of added module scores was realized by ‘FeaturePlot’ function in Seurat.

Comparing cell subsets proportion

The cell type proportion for each sample was determined by dividing the total number of cells by the number of a particular cell type in each group. The proportions of cell subset among pneumonia and control groups were tested using the Wilcoxon rank-sum method and visualized in boxplot. For CD8+ T cells, the correlation of cell proportion between cycling cells and other cell types were conducted using the Spearman method. The overall proportion of each cell type across three sample types was calculated and visualized in stacked barplot.

Trajectory analysis

Loom files containing spliced and unspliced counts for trajectory analysis were generated from BAM files in cellranger outputs using ‘run10x’ command in ‘velocyto’(11) and GRCh38 human annotations. Loom files were then combined together. Information of intended cells for trajectory analysis, including metadata, cellular barcode, UMAP coordinates, expression counts matrix, PCA reduction matrix and gene names in R were saved, later integrated into anndata form in Python, and merged with loom files. The scVelo(12) workflow was followed by default in accordance to the ‘Dynamical Modeling’ tutorial. Latent time information was extracted as csv file from anndata’s metadata and was integrated with corresponding seurat object by cellular barcodes. The fitted curve of gene expression and latent time for each cell was calculated by ‘stat_smooth’ and visualized by ggplot2.

Influenza B virus RNA positive cells detection

Clean FASTQ sequencing data were used for the detection of influenza B virus RNA reads at single-cell level. Human index file was constructed with human genome GRCh38.p13 from NCBI as the reference library sequence, with which clean reads were compared using the Bowtie2 (version 2.3.5.1) to remove the human source sequence (end-to-end mode, k = 1). Kraken2 index file was constructed with the reference of NT library sequence from NCBI. Kraken2 (version 2.1.1) was used to compare and classify nonhuman reads obtained previously (confidence = 0.5), and the corresponding classification results of each sequence were obtained. Then, Reads belonging to reads2 in double-ended reads were screened out and label line were recorded. All the obtained label lines were searched for corresponding sequence in reads1, and the first 16 digits was extracted as barcode. Taking one mismatch as the threshold, barcodes were matched with known single-cell barcodes. Reads number of influenza B virus were integrated to patient 6’s metadata by matching barcodes.

Cellchat

Cell-cell interactions was inferred using R package ‘Cellchat’(13) across tMDM_CCL2 in BALF with CD8+ T cells, CD4+ T cells, B/plasma cells, AM_FABP4 with other monocytes/macrophages clusters, and ciliated cells in BALF with CD8+ T cells, B/plasma cells and monocytes/macrophages. Cells used for each analysis were subset and merged together, with their idents set to cell type. The database of CellChatDB. human was set to ‘Secreted Signaling’ for roles of cytokines and chemokines were highlighted. Major steps of analysis were in

accordance with the tutorial in the CellChat github repository. The 'netVisual_bubble' function was used to show the most important ligand-receptor pairings that cause the changes in cell-cell interaction across comparisons. The cell-cell communication at a signaling pathway level was inferred by 'computeCommunProbPathway' function with selected particularly sportified ligand-receptor pairings, and visualization of interaction strength was realized by function 'netVisual_aggregate'. CellChat communication probabilities are estimated from the average ligand and receptor expression within defined sender and receiver cell populations. The associated p-values are derived from permutation-based testing implemented in CellChat, in which cell-type labels are randomly shuffled to generate a null distribution of communication probabilities. Donor heterogeneity is not explicitly modeled and that these analyses should be interpreted as hypothesis-generating. The MHC-I communication probability score was calculated across cells with influenza B virus RNA detected and P6's CD8+ T cells whose TCR clone expansion were over 10. The ident of these merged cells was set to 'fluB' and 'TCR ID'. Cell-Cell Contact database was used and MHC-I pathway was selected. Each TCR-ID's communication probability with influenza B virus RNA positive cells was extracted from 'netP' slot. Statistical comparisons of within-patient clonal heterogeneity were performed at the TCR clone level.

Statistics

Statistics were run in accordance with the legends of the figures. To calculate relationships between cell subsets, Pearson correlation was utilized. According to data's distribution, the Kruskal-Wallis test, Wilcoxon test and Student T test were used to compare means between groups. For adjusted p value calculation in finding DEGs, Benjamini-Hochberg technique was used to determine statistical significance. Statistical significance was defined as p values or adjusted p values less than 0.05.

Data visualization

Using customized code based on R package ggplot2 and its extensions, plots were produced. Palette was chosen with the help of R package Rcolorbrewer and Viridis. The dplyr, tidyr, and stringr packages were used for data manipulation.

Reference

1. JP Metlay, GW Waterer, AC Long, A Anzueto, J Brozek, K Crothers, LA Cooley, NC Dean, MJ Fine, SA Flanders, MR Griffin, ML Metersky, DM Musher, MI Restrepo, CG Whitney. Diagnosis and Treatment of Adults with Community-acquired Pneumonia. An Official Clinical Practice Guideline of the American Thoracic Society and Infectious Diseases Society of America. AM J RESP CRIT CARE. 2019;200:e45-e67.
2. N Borcherding, NL Bormann, G Kraus. scRepertoire: An R-based toolkit for single-cell immune receptor analysis. F1000Res. 2020;9:47.
3. L Zhang, X Yu, L Zheng, Y Zhang, Y Li, Q Fang, R Gao, B Kang, Q Zhang, JY Huang, H Konno, X Guo, Y Ye, S Gao, S Wang, X Hu, X Ren, Z Shen, W Ouyang, Z Zhang. Lineage tracking reveals dynamic relationships of T cells in colorectal cancer. NATURE. 2018;564:268-272.
4. D Aran, AP Looney, L Liu, E Wu, V Fong, A Hsu, S Chak, RP Naikawadi, PJ Wolters, AR Abate, AJ Butte, M Bhattacharya. Reference-based analysis of lung single-cell sequencing reveals a transitional profibrotic macrophage. NAT IMMUNOL. 2019;20:163-172.
5. G Finak, A McDavid, M Yajima, J Deng, V Gersuk, AK Shalek, CK Slichter, HW Miller, MJ McElrath, M Prlic, PS Linsley, R Gottardo. MAST: a flexible statistical framework for assessing transcriptional changes and characterizing heterogeneity in single-cell RNA sequencing data. GENOME BIOL. 2015;16.
6. D Gadala-Maria, G Yaari, M Uduman, SH Kleinstein. Automated analysis of high-throughput B-cell sequencing data reveals a high frequency of novel immunoglobulin V gene segment alleles. Proceedings of the National Academy of Sciences. 2015;112.
7. NT Gupta, JA Vander Heiden, M Uduman, D Gadala-Maria, G Yaari, SH Kleinstein. Change-O: a toolkit for analyzing large-scale B cell immunoglobulin repertoire sequencing data. BIOINFORMATICS. 2015;31:3356-3358.
8. N Nouri, SH Kleinstein. A spectral clustering-based method for identifying clones from high-throughput B cell repertoire sequencing data. BIOINFORMATICS. 2018;34:i341-i349.
9. Y Hao, S Hao, E Andersen-Nissen, WM Mauck, S Zheng, A Butler, MJ Lee, AJ Wilk, C Darby, M Zager, P Hoffman, M Stoeckius, E Papalexi, EP Mimitou, J Jain, A Srivastava, T Stuart, LM Fleming, B Yeung, AJ Rogers, JM McElrath, CA Blish, R Gottardo, P Smibert, R Satija. Integrated analysis of multimodal single-cell data. CELL. 2021;184:3573-3587.

10. G Yu, L Wang, Y Han, Q He. clusterProfiler: an R Package for Comparing Biological Themes Among Gene Clusters. OMICS: A Journal of Integrative Biology. 2012;16:284-287.
11. G La Manno, R Soldatov, A Zeisel, E Braun, H Hochgerner, V Petukhov, K Lidschreiber, ME Kastriti, P Lönnerberg, A Furlan, J Fan, LE Borm, Z Liu, D van Bruggen, J Guo, X He, R Barker, E Sundström, G Castelo-Branco, P Cramer, I Adameyko, S Linnarsson, PV Kharchenko. RNA velocity of single cells. NATURE. 2018;560:494-498.
12. V Bergen, M Lange, S Peidli, FA Wolf, FJ Theis. Generalizing RNA velocity to transient cell states through dynamical modeling. NAT BIOTECHNOL. 2020;38:1408-1414.
13. S Jin, CF Guerrero-Juarez, L Zhang, I Chang, R Ramos, C Kuan, P Myung, MV Plikus, Q Nie. Inference and analysis of cell-cell communication using CellChat. NAT COMMUN. 2021;12.

Supplementary figures

a

Clinical information of validation cohort

Patient ID	Sex	Age	Outcome	APACHEII	SOFA	Imaging Findings	WBC (x10⁹/L)	Neutrophils (%)	Lymphocytes (x10⁹/L)	Lymphocytes (%)	IL-6 (pg/mL)	PCT (ng/mL)	CRP (mg/L)
V1	M	70-80	Death	37	11	★ (Patchy)	12.56	92.4	4.2	0.53	183.6	6.21	244
V2	M	30-40	Survival	23	8	★ (Patchy), ★ (GGO)	24.12	83.3	7.6	1.83	130	0.38	151
V3	M	70-80	Death	25	14		17.66	90.4	4.5	0.79	209	0.21	155
V4	M	50-60	Survival	29	7	★ (Patchy), ★ (Consolidation), ★ (GGO)	4.26	76.3	15.3	0.65	28.5	0.21	68.3

★ Patchy ★ Consolidation ★ GGO

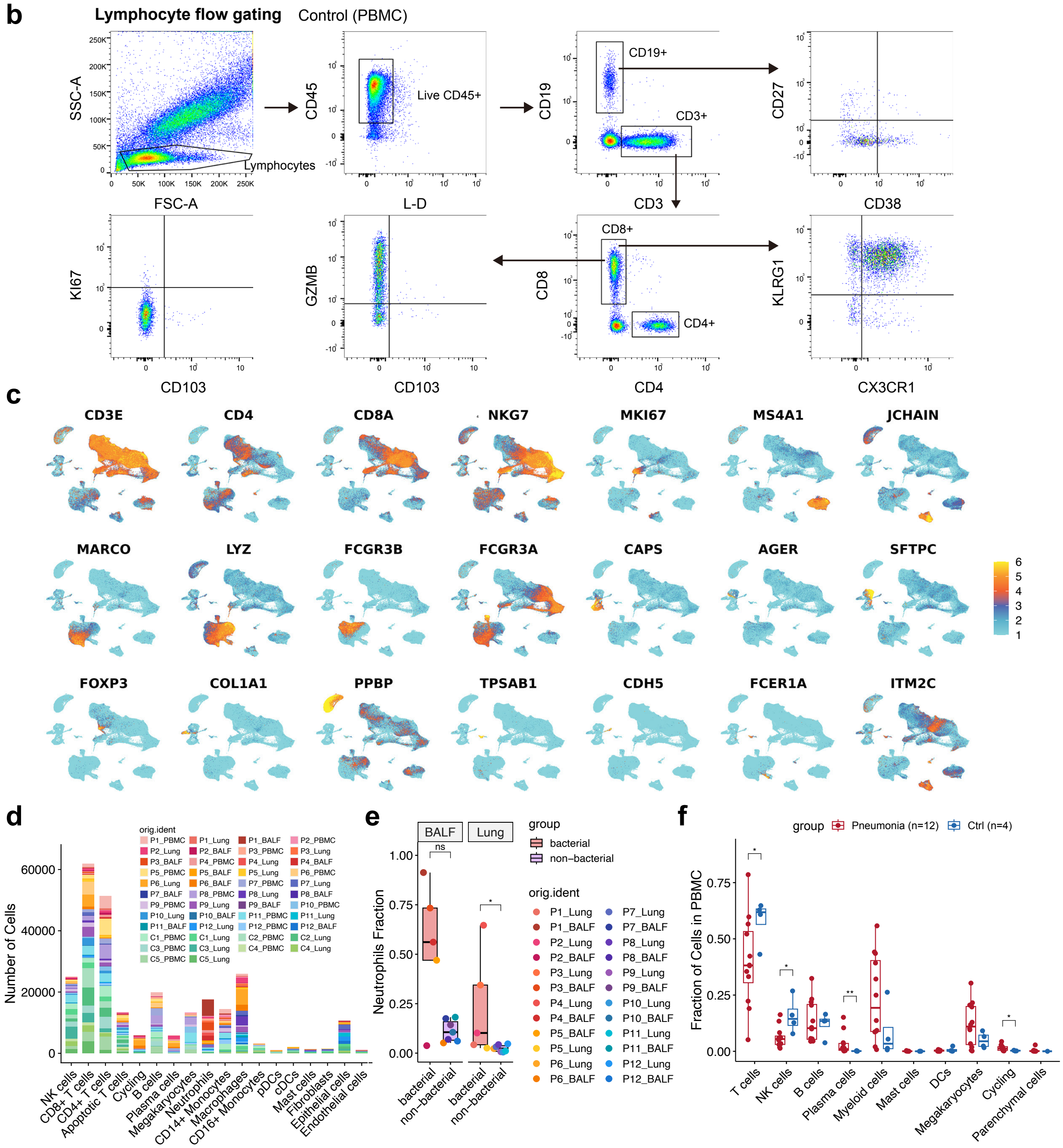

Figure. S1. Global analyses of cell composition in patients and donors, related to Figure 1.

a) Detailed clinical information of Cohort 2, including sex, age, imaging findings and part of laboratory test for each patient. b) Gating strategies of CD103+ GZMB+ CD8+ T cells and CD27+ CD38+ plasma cells from Donor 4. c) Marker gene expression of each cell type in the UMAP. Color-coding of each cell is based on log normalized expression. d) Stacked bar plot showing total cell number of each cell type, color-coded by different sample origin. e) Major cell type fractions in the pneumonia (n = 12) versus control (n = 4) PBMCs, shown in boxplots. Donor 2 was excluded. Middle line, median; box edges, 25th and 75th percentiles; *$p < 0.05$; **$p < 0.01$. Wilcoxon rank-sum test. n refers to the number of subjects. f) Neutrophils fraction of patients with bacterial and non-bacterial infection in BALF and lung.

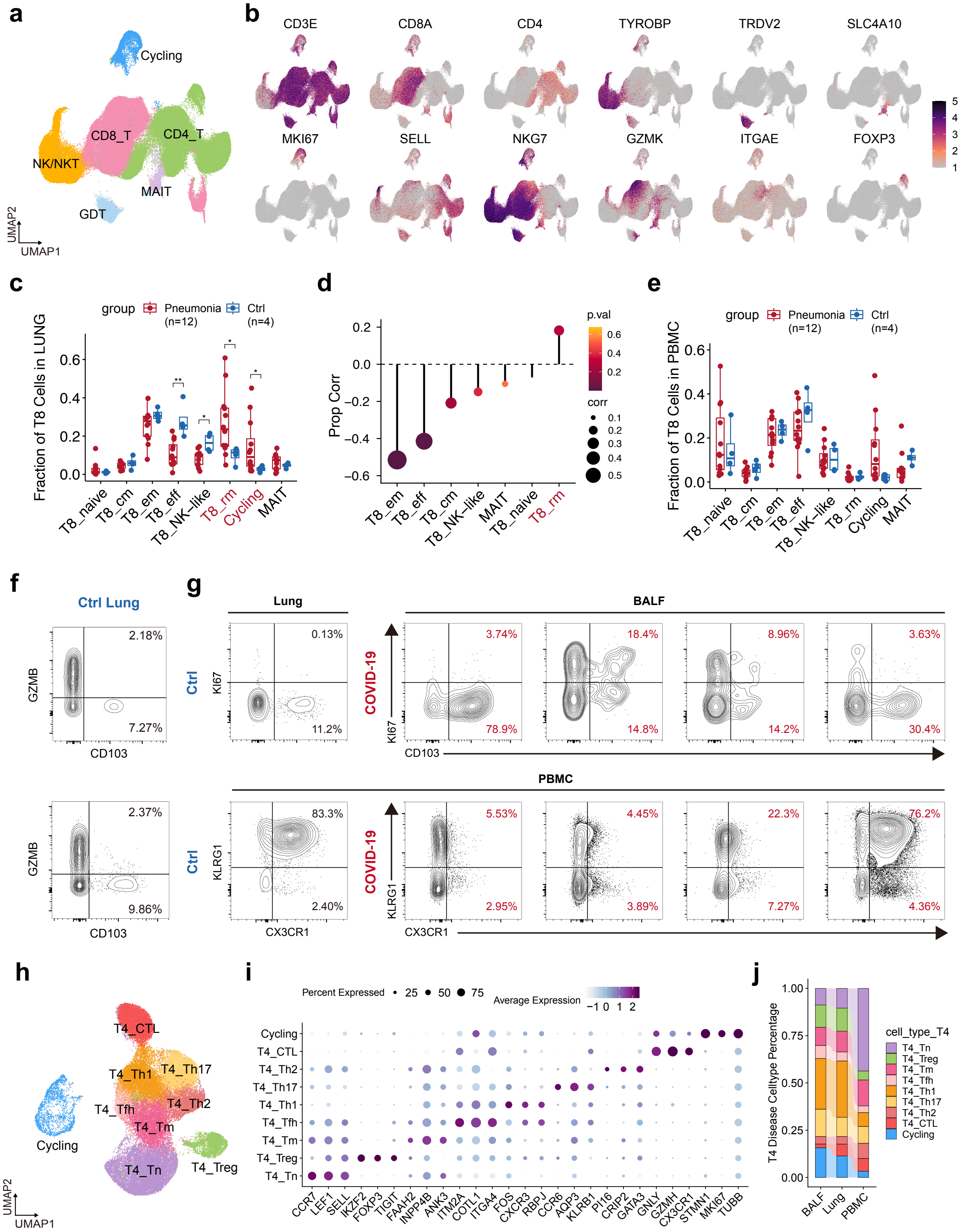

Figure. S2. scRNA-seq profiling of T cells in patients and donors, related to Figure 2

a) UMAP of 136,127 T and NK cells, and cell type annotation of major clusters. b) Feature plots showing color-coded marker gene expression projected onto the UMAP embedding in a). c) Fractions of CD8+ T cell subclusters

in the pneumonia and control lungs, shown in boxplots. *$p < 0.05$; **$p < 0.01$. Wilcoxon rank-sum test. n refers to the number of subjects. d) The correlation between cycling CD8+ T cells and other subclusters. Length of lines and the size of dot represents correlation coefficient, while the color of dots indicates p value. Spearman's Rank correlation coefficient. e) Fractions of CD8+ T cell subclusters in the pneumonia and control PBMC. *$p < 0.05$; **$p < 0.01$. Wilcoxon rank-sum test. n refers to the number of subjects. f) Percentage of T cell subsets based on CD103 and GZMB expression in lung of Donor 3 and 4. g) High proportion of Ki67+ CD103+ CD8+ T cells were detected from BALF of patients with severe COVID-19. Decreased proportion of CX3CR1+ KLRG1+ CD8+ T cells were detected from peripheral blood of patients with severe COVID-19 compared to that of controls. h) UMAP of 55,336 CD4+ T cells in a). Cell-type annotation is on the basis of canonical marker genes expression. i) Dot plot showing scaled, log-normalized expression of marker genes for each cluster in c). j) Relative proportion of CD4+ T subclusters in pneumonia group among 3 sample types, colored as in c).

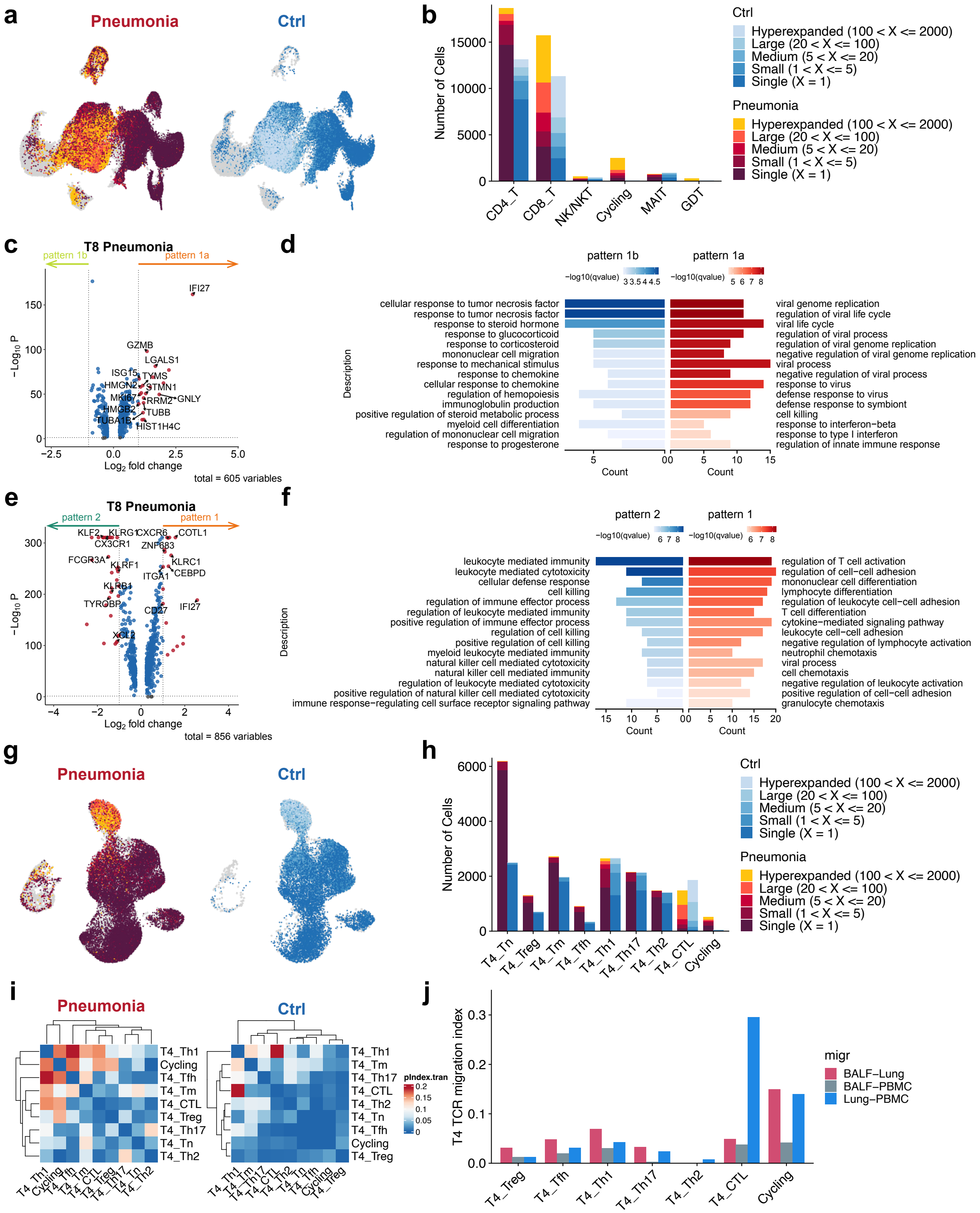

Figure. S3. scTCR-seq profiling of T cells in patients and donors, related to Figure 3

a) UMAP embedding of T and NK cells colored by TCR clonetypes, and split into pneumonia and control groups. b) Sum of TCR expanded cells in each T and NK subcluster among pneumonia and control groups. c) Volcano plots of DEGs between TCR pattern 1a and 1b. d) GO enrichment of DEGs in C) showing up- and down-regulated biological processes between TCR pattern 1a and 1b. e) Volcano plots of DEGs between TCR pattern 1 (1a and 1b) and 2. X axis, fold change of gene expression (>= 1); Y axis, statistical significance ($p < 0.05$). f) GO enrichment of

DEGs in e) showing up- and down-regulated biological processes between TCR pattern 1 and 2. GO Gene counts and q value of each pathway are displayed. g) UMAP embedding of CD4+ T cells colored by TCR clonetypes, and split into pneumonia and control groups. h) Sum of TCR expanded cells in each CD4+ T cluster among pneumonia and control groups, shown with stacked barplot and color-coded by different TCR clonetype. i) Heatmap showing the transition index among CD4+ T cell subclusters in pneumonia and control group separately. j) Bar plots of CD4+ T cell migration index among 3 sample types in each subcluster, color-coded by different migration type.

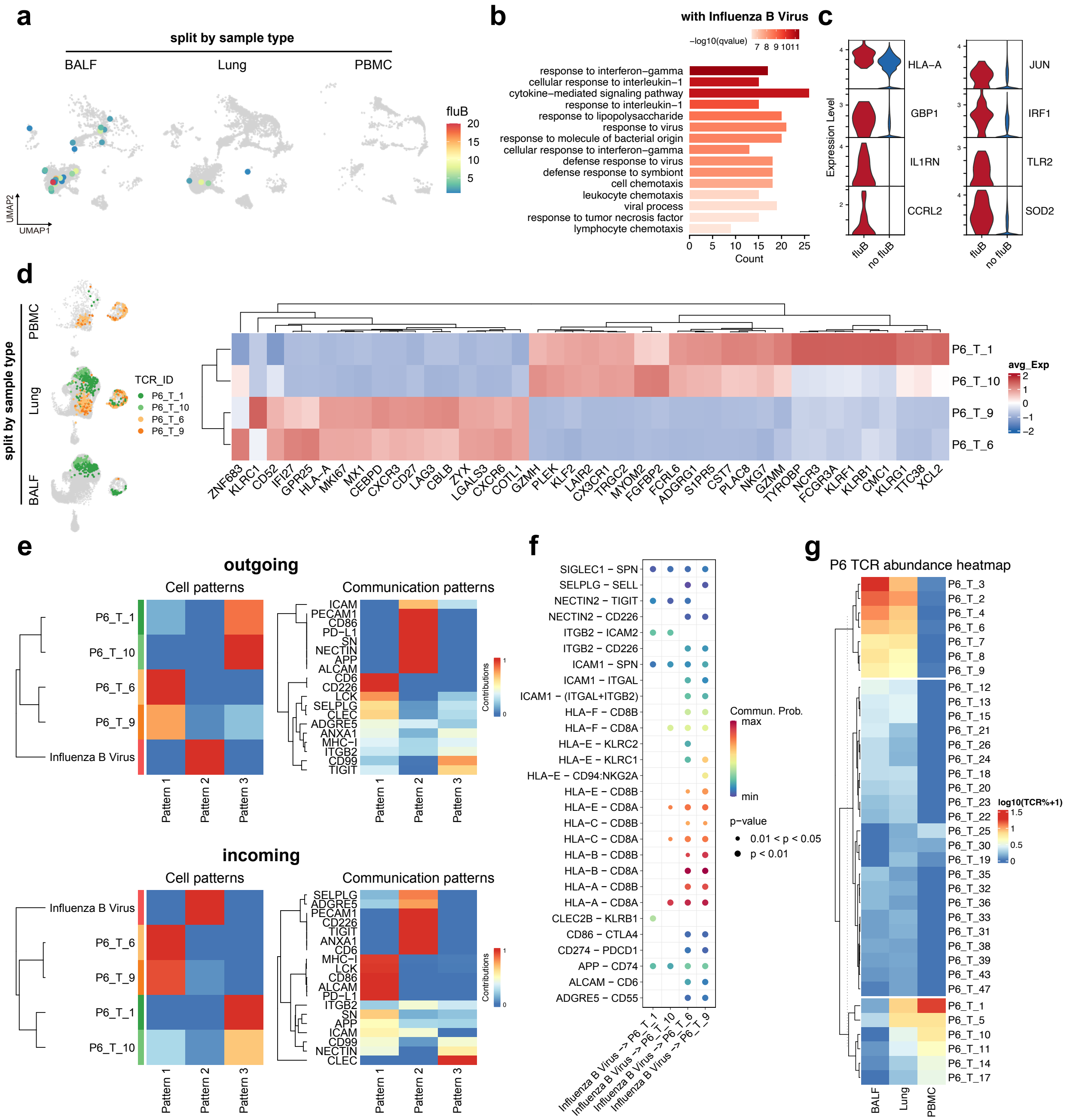

Figure. S4. Functional features of activated CD8+ T cells during migration, related to Figure 4

a) Projection of cells with influenza B virus RNA detected among sample types into UMAP embedding, colored by flu read detected in each cell. b) Up-regulated biological processes of P6 flu reads positive cells of GO enrichment. X axis, GO gene counts; color-coding, q value of each pathway. c) Violin plots of log-normalized gene mRNA expression (HLA-A, GBP1, IL1RN, CCRL2, JUN, IRF1, TLR2 and SOD2) in P6 cells with or without flu read respectively. d) Heatmap showing expression of 4 typical TCRs' DEGs. Both row and columns are clustered. Left, distribution of the selected TCRs among sample types, projected on the UMAP, indicates their TCR pattern. e) Heatmap of major signals for 5 cell subsets and their communication patterns in outgoing or incoming way separately. f) Bubble plot showing statistically significant enrichment of ligand-receptor interactions (rows) from flu RNA positive cells to 4 selected TCRs (columns). Dot color indicates communication probabilities and dot size represents p value. g) TCR abundance heatmap of P6 TCRs whose clonetype frequency are over 10. All TCRs (columns) are hierarchically clustered into 3 compartments, first and second of which defined as pattern 1a and the third as pattern 2.

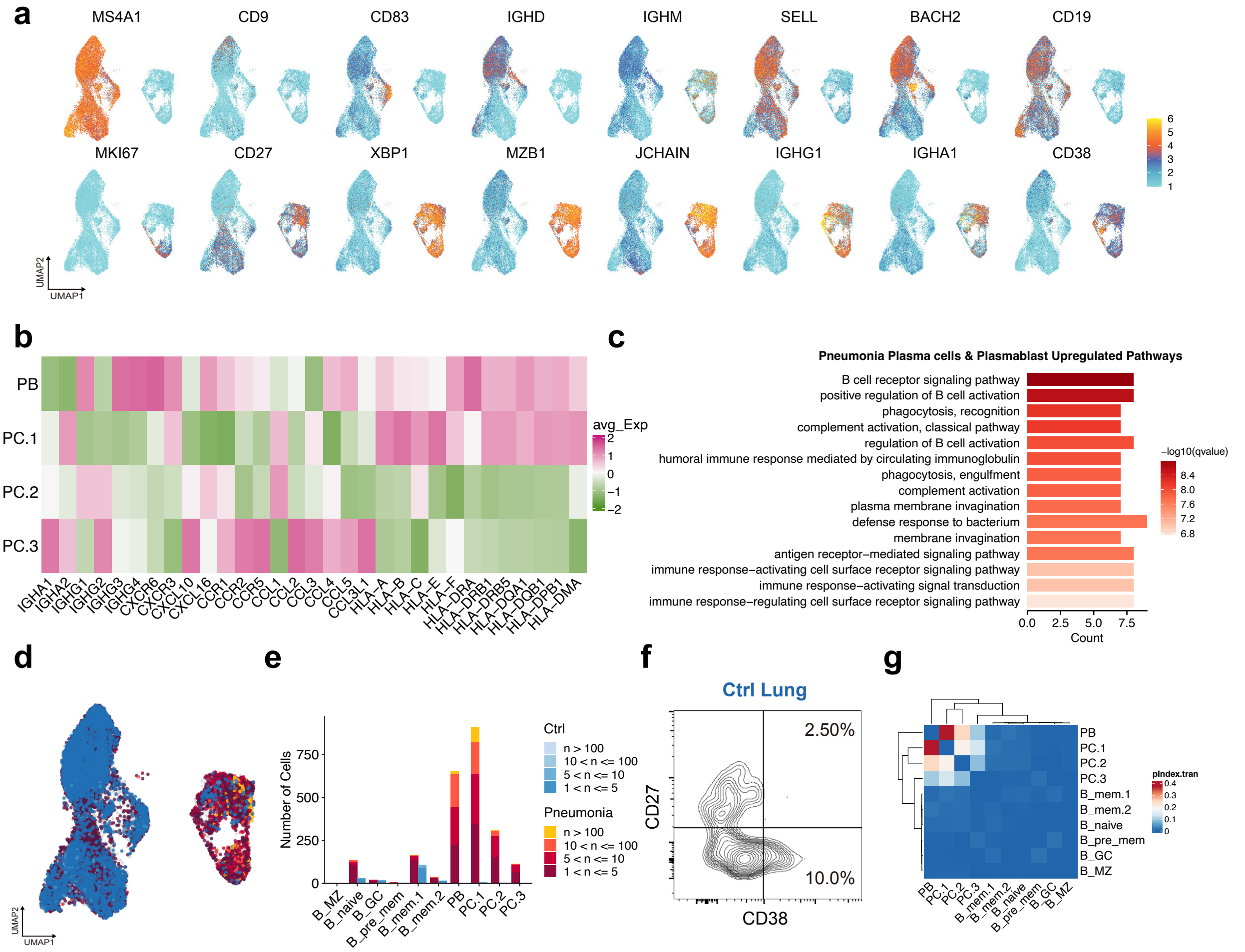

Figure. S5. scRNA-seq and scBCR-seq profiling of B cells in patients and donors, related to Figure 5

a) Feature plots showing color-coded marker gene expression projected onto the UMAP. b) Heatmap of indicated gene expression among plasmablasts and plasma cell subclusters. c) Up-regulated biological processes of plasma cells and plasmablasts in pneumonia versus control by GO enrichment. X axis, GO gene counts; color-coding, q value of each pathway. d) UMAP embedding of B and plasma cells, colored by BCR clonetypes and groups. e) Sum of BCR expanded cells in each B and plasma subcluster among pneumonia and control groups. Cells with single BCR expansion are omitted. f) Percentage of plasma cell subsets based on CD27 and CD38 expression in lung of Donor 4. g) Heatmap showing the transition index between B and plasma cell subclusters.

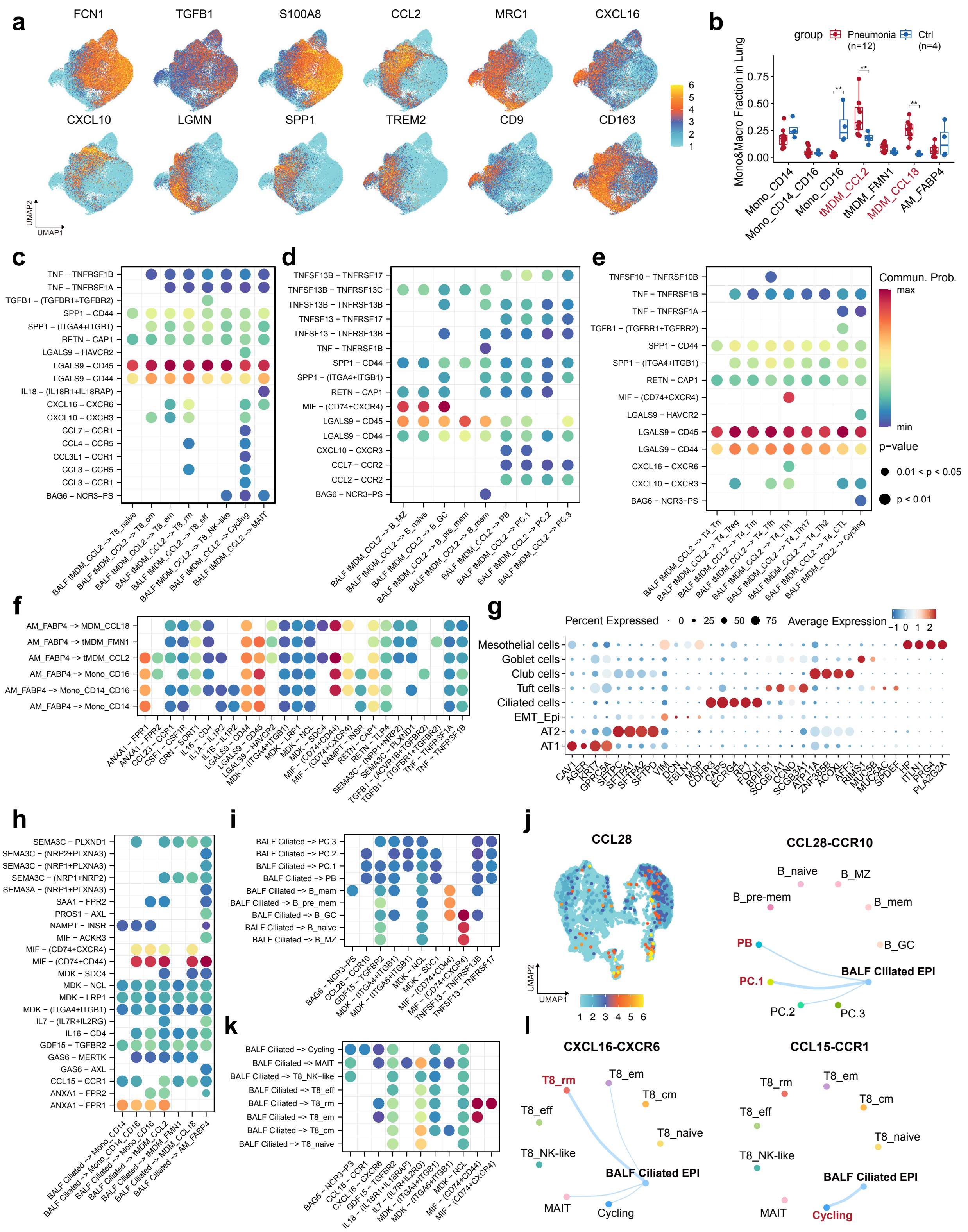

Figure. S6. scRNA-seq profiling of the mono-macrophages in patients and donors, related to Figure 6
a) Feature plots showing color-coded monocytes and macrophages marker gene expression projected onto the UMAP. b) Boxplots of monocytes and macrophages subcluster fractions in the pneumonia (n = 12) versus control (n

= 4) lungs. *$p < 0.05$; **$p < 0.01$. Wilcoxon rank-sum test. n refers to the number of subjects. c-e) Bubble plot showing statistically significant enrichment of ligand-receptor interactions (rows) from transitioning_MDM_CCL2 in BALF to subclusters of CD8+ T cells, B and plasma cells and CD4+ T cells, respectively. f) Bubble plot illustrating the ligand-receptor interactions from AM_FABP4 to other monocytes and macrophages subclusters. g) Dot plot showing scaled, log-normalized expression of marker genes for each cluster. h) Bubble plot illustrating the ligand-receptor interactions from ciliated cells in BALF to monocytes and macrophages subclusters. i) Bubble plot illustrating the ligand-receptor interactions from ciliated cells in BALF to B and plasma subclusters. j) Left, Feature plots of CCL28 expression projected onto the UMAP. Right, circle plot showing inferred CCL28-CCR10 signaling interaction strength of ciliated cells in BALF with subclusters of B and plasma cells. k) Bubble plot illustrating the ligand-receptor interactions from ciliated cells in BALF to B and plasma subclusters. Dot color indicates communication probabilities and dot size represents p value. l) Inferred CCL16-CXCR6 (left) and CCL15-CCR1 (right) signaling interaction strength of ciliated cells in BALF with subclusters of CD8+ T cells, shown in circle plots. The thickness of lines indicates strength of interactions.

Supplementary tables

TableS1. Clinical information of enrolled patients and donors, related to Figure 1.

A	B	C	D	E	F	G	H	I	J	K	L	M	N	O	P	Q	R	S
ID	Group	Sex	Age	Smoking Status	Pre-existing Disease	Number of Days: Admission to Biopsy/ Sampling	Number of days: IMV to biopsy/sampling	Outcome	APACHEII	SOFA	Pathogen detected	IL-6 (pg/mL)	PCT (ng/mL)	CRP (mg/L)	WBC (×10^9/L)	Neu (%)	Lymph (×10^9/L)	Lymph (%)
P1	P	M	O	Y	NA	11	3	D	32	16	Mycobacterium tuberculosis	24.1	1.50	77.4	14.63	89.5	0.51	3.5
P2	P	M	O	N	HT	4	4	S	32	10	Legionella pneumophila	20.4	7.45	66.6	14.16	89.8	0.62	4.4
P3	P	F	M	N	HT	3	3	D	35	8	Klebsiella pneumoniae, Staphylococcus aureus, Acinetobacter baumannii	>5000.0	14.67	441.0	7.27	92.0	0.36	5.0
P4	P	M	O	Y	NA	3	17	S	32	17	Legionella longbeachae	320.0	2.36	222	6.15	89.3	0.32	5.2
P5	P	F	M	N	Hypothyroidism	4	10	S	28	6	Aspergillus, Cytomegalovirus, Escherichia coli	75.7	0.13	40.8	4.56	80.3	0.57	12.5
P6	P	F	Y	N	Hepatitis B	1	1	S	26	13	Influenza B virus	223.0	0.47	13.6	2.77	53.8	1.12	40.4
P7	P	M	O	N	DM, HT, LC, CHD	5	11	D	24	7	Undetected	91.0	0.38	173.0	17.05	72.8	1.40	8.2
P8	P	M	O	N	LC	19	20	D	32	9	Undetected	9.0	0.81	105	21.14	92.4	0.68	3.2
P9	P	M	O	Y	NA	1	1	D	26	7	Undetected	13.7	0.33	117	19.14	91.4	1.01	5.3
P10	P	F	O	N	HT	1	1	S	27	10	cytomegalovirus	7.1	0.41	99.1	20.72	96.2	0.52	2.5
P11	P	M	O	N	LC	3	2	D	24	11	Undetected	179.6	0.80	187.0	11.02	82.8	0.45	4.1
P12	P	F	O	N	NA	0	2	D	29	11	Pneumocystis carinii	23.5	0.19	45.00	17.8	95.1	0.41	2.3
V1	V	M	O	Y	COPD	4	1	D	37	11	SARS-CoV-2	183.6	6.21	244	20.65	91.9	0.78	3.8
V2	V	M	Y	N	Hepatitis B	1	1	S	23	8	SARS-CoV-2	130.0	0.38	151	21.14	96.0	0.55	2.6
V3	V	M	O	Y	Aspergillosis, Thyroid cancer	2	2	D	25	14	SARS-CoV-2, Aspergillus, Candida albicans	209.0	0.21	155	17.66	90.4	0.79	4.5
V4	V	M	M	Y	DM,HT	6	6	S	29	7	SARS-CoV-2	28.5	0.21	68.3	4.26	76.3	0.65	15.3
C1	D	F	M	N	NA	NA	NA	NA	NA	NA	Undetected	ND	ND	ND	3.09	56.1	0.89	28.8
C2	D	F	O	N	DM,HT	NA	NA	NA	NA	NA	Pneumocystis jirovecii	ND	ND	ND	5.61	48.5	2.24	39.9
C3	D	M	Y	Y	NA	NA	NA	NA	NA	NA	Undetected	ND	ND	ND	6.84	50.0	2.45	35.8
C4	D	M	O	Y	CHD	NA	NA	NA	NA	NA	Undetected	1.9	<0.02	3.39	3.76	63.2	1.00	26.6
C5	D	F	M	N	HT	NA	NA	NA	NA	NA	Undetected	ND	ND	ND	6.69	50.3	2.66	39.8

Legends:
Colume B: Group. P(Pneumonia), V(Validation), D(Donor)
Column C: Sex. M(Male), F(Female)
Column D: Age. Y(Young age, 18-39), M(Middle age, 40-59), O(Old age, 60+)
Column E: Smoking Status. Y(Yes), N(No)
Column F: Pre-existing Disease. HT(Hypertension), DM(Diabetes mellitus), LC(Lung cancer), CHD(Coronary heart disease), COPD(Chronic obstructive pulmonary disease), NA (Not Applicable)
Column I: Outcome. S(Survival), D(Death)
Column Q: Neutrophils (%)
Column R: Lymphocytes (×10^9/L)
Column S: Lymphocytes (%)

Table S1. Clinical information of enrolled patients and donors.

Table S2A. Cluster-specific marker gene lists for all major cell types, related to Figure 1.

p_val	avg_log2FC	pct.1	pct.2	p_val_adj	cluster	gene
0	2.30363997	0.671	0.123	0	CD8+ T cells	CD8A
0	2.0915093	0.931	0.38	0	CD8+ T cells	CCL5
0	2.03328648	0.754	0.187	0	CD8+ T cells	GZMH
0	1.95733787	0.606	0.088	0	CD8+ T cells	CD8B
0	1.87977462	0.419	0.082	0	CD8+ T cells	GZMK
0	1.7394243	0.904	0.333	0	CD8+ T cells	NKG7
0	1.71580671	0.873	0.256	0	CD8+ T cells	GZMA
0	1.63998071	0.918	0.329	0	CD8+ T cells	CD3D
0	1.58156385	0.968	0.45	0	CD8+ T cells	IL32
0	1.57491697	0.955	0.379	0	CD8+ T cells	CD3E
0	2.15250602	0.916	0.248	0	CD4+ T cells	IL7R
0	1.91329542	0.916	0.312	0	CD4+ T cells	LTB
0	1.79973932	0.742	0.157	0	CD4+ T cells	TCF7
0	1.70017557	0.704	0.089	0	CD4+ T cells	LEF1
0	1.41987963	0.92	0.534	0	CD4+ T cells	LDHB
0	1.37950167	0.532	0.029	0	CD4+ T cells	MAL
0	1.37834334	0.543	0.079	0	CD4+ T cells	CCR7
0	1.27491822	0.855	0.465	0	CD4+ T cells	GAS5
0	1.24266323	0.732	0.354	0	CD4+ T cells	NOSIP
0	1.21199164	0.996	0.939	0	CD4+ T cells	TPT1
0	4.70883801	0.52	0.056	0	Macrophages	APOE
0	4.53557444	0.55	0.057	0	Macrophages	APOC1
0	3.96367251	0.48	0.033	0	Macrophages	CCL2
0	3.87553549	0.576	0.04	0	Macrophages	C1QB
0	3.80859982	0.599	0.042	0	Macrophages	C1QA
0	3.69921698	0.586	0.035	0	Macrophages	C1QC
0	3.65455462	0.976	0.329	0	Macrophages	CTSB
0	3.28814337	0.998	0.963	0	Macrophages	FTL
0	3.21696566	0.764	0.068	0	Macrophages	CTSL
0	3.18134027	0.985	0.304	0	Macrophages	IFI30
0	3.17080891	0.362	0.058	0	NK cells	PTGDS
0	2.90851035	0.867	0.172	0	NK cells	SPON2
0	2.83337518	0.899	0.239	0	NK cells	GNLY
0	2.7157605	0.98	0.318	0	NK cells	PRF1
0	2.66450751	0.865	0.143	0	NK cells	FGFBP2
0	2.47926806	0.991	0.409	0	NK cells	NKG7
0	2.43616794	0.951	0.244	0	NK cells	GZMB
0	2.28952781	0.97	0.312	0	NK cells	CTSW
0	2.24140456	0.826	0.064	0	NK cells	KLRF1
0	2.17182059	0.802	0.16	0	NK cells	CLIC3
0	5.35897582	0.566	0.058	0	Neutrophils	CXCL8
0	5.33395551	0.448	0.007	0	Neutrophils	PI3
0	4.3678374	0.854	0.283	0	Neutrophils	NAMPT
0	4.33928443	0.838	0.449	0	Neutrophils	SOD2
0	4.15599564	0.533	0.063	0	Neutrophils	C15orf48
0	3.81520717	0.662	0.131	0	Neutrophils	DOCK4
0	3.75794086	0.67	0.079	0	Neutrophils	AQP9
0	3.59984984	0.671	0.124	0	Neutrophils	CSF3R
0	3.50588355	0.519	0.037	0	Neutrophils	G0S2
0	3.44122965	0.953	0.69	0	Neutrophils	NEAT1
0	4.30014592	0.997	0.271	0	CD14+ Monoc	S100A9
0	4.18345706	0.998	0.241	0	CD14+ Monoc	S100A8
0	3.91152546	0.958	0.211	0	CD14+ Monoc	LYZ
0	3.72418407	0.928	0.081	0	CD14+ Monoc	S100A12
0	3.52234263	0.903	0.11	0	CD14+ Monoc	VCAN
0	2.96880381	0.894	0.083	0	CD14+ Monoc	FCN1
0	2.74888549	0.924	0.14	0	CD14+ Monoc	MNDA
0	2.11944421	0.889	0.29	0	CD14+ Monoc	CEBPD
0	2.08406563	0.777	0.085	0	CD14+ Monoc	CSTA
0	2.03613533	0.929	0.479	0	CD14+ Monoc	CTSS
0	2.17404349	0.618	0.296	0	Apoptotic T cel	INPP4B
0	2.11401681	0.876	0.503	0	Apoptotic T cel	PRKCH
0	2.10393232	0.883	0.503	0	Apoptotic T cel	SKAP1
0	2.07432541	0.574	0.337	0	Apoptotic T cel	BCL2
0	2.06774156	0.651	0.32	0	Apoptotic T cel	THEMIS
0	2.06116223	0.586	0.275	0	Apoptotic T cel	CAMK4
0	2.0580903	0.425	0.199	0	Apoptotic T cel	ANK3
0	2.04927601	0.923	0.692	0	Apoptotic T cel	ARHGAP15
0	2.02453026	0.591	0.314	0	Apoptotic T cel	PDE3B
0	1.99428595	0.709	0.341	0	Apoptotic T cel	KLF12
0	7.28730841	0.99	0.115	0	Megakaryocyte	PPBP
0	6.71547066	0.993	0.155	0	Megakaryocyte	NRGN
0	5.7649179	0.956	0.048	0	Megakaryocyte	PF4
0	5.7566817	0.957	0.043	0	Megakaryocyte	TUBB1
0	5.44720112	0.958	0.086	0	Megakaryocyte	PRKAR2B
0	5.33685857	0.944	0.06	0	Megakaryocyte	CAVIN2
0	5.24225537	0.88	0.071	0	Megakaryocyte	MYL9
0	5.20631471	0.926	0.04	0	Megakaryocyte	MPIG6B
0	4.9903081	0.939	0.075	0	Megakaryocyte	SPARC
0	4.98808817	0.879	0.025	0	Megakaryocyte	GP9
0	3.97741123	0.979	0.029	0	B cells	MS4A1
0	3.88836744	0.96	0.034	0	B cells	CD79A
0	3.55680026	0.937	0.036	0	B cells	BANK1
0	3.19811131	0.878	0.05	0	B cells	AFF3
0	2.95083169	0.999	0.811	0	B cells	CD74
0	2.80248541	0.493	0.005	0	B cells	TCL1A
0	2.77201692	0.877	0.091	0	B cells	RALGPS2
0	2.72747217	0.993	0.407	0	B cells	HLA-DRA
0	2.70419905	0.789	0.009	0	B cells	FCRL1
0	2.54804566	0.871	0.097	0	B cells	CD79B
0	7.49557882	0.425	0.09	0	Epithelial cells	SFTPC
0	5.96225716	0.313	0.039	0	Epithelial cells	SCGB1A1
0	5.75354986	0.379	0.036	0	Epithelial cells	SFTPA1
0	5.58003908	0.46	0.036	0	Epithelial cells	SFTPB
0	5.34174668	0.393	0.029	0	Epithelial cells	SCGB3A1
0	5.27209299	0.381	0.027	0	Epithelial cells	SFTPA2
0	4.67194095	0.841	0.046	0	Epithelial cells	SLPI
0	4.23634113	0.811	0.009	0	Epithelial cells	WFDC2
0	3.65978774	0.508	0.024	0	Epithelial cells	CAPS
0	3.46569405	0.361	0.015	0	Epithelial cells	NAPSA
0	3.36724959	0.929	0.2	0	Cycling	STMN1
0	2.64609701	0.961	0.635	0	Cycling	TUBA1B
0	2.60458919	0.917	0.382	0	Cycling	HMGB2
0	2.57047171	0.811	0.437	0	Cycling	HIST1H4C
0	2.5702911	0.776	0.022	0	Cycling	TYMS
0	2.55542813	0.941	0.553	0	Cycling	TUBB
0	2.49694586	0.724	0.022	0	Cycling	MKI67
0	2.47783749	0.666	0.021	0	Cycling	RRM2
0	2.32639525	0.946	0.616	0	Cycling	HMGN2
0	2.08333647	0.797	0.298	0	Cycling	GZMB
0	6.86545936	0.294	0.026	0	Plasma cells	IGLV6-57
0	6.77471531	0.681	0.09	0	Plasma cells	IGHG1
0	6.69628407	0.319	0.08	0	Plasma cells	IGLV2-14
0	6.59144901	0.339	0.08	0	Plasma cells	IGKV3-20
0	6.45616781	0.269	0.055	0	Plasma cells	IGLV1-44
0	6.45180423	0.382	0.053	0	Plasma cells	IGKV4-1
0	6.23591908	0.631	0.127	0	Plasma cells	IGKC
0	6.22388164	0.276	0.055	0	Plasma cells	IGHV3-23
0	6.04661214	0.961	0.108	0	Plasma cells	JCHAIN
0	5.6380185	0.678	0.097	0	Plasma cells	IGHA1
0	3.66806259	0.996	0.212	0	CD16+ Monoc	LST1
0	3.30466475	0.87	0.055	0	CD16+ Monoc	CDKN1C
0	3.10094825	0.996	0.237	0	CD16+ Monoc	AIF1
0	2.92051531	0.963	0.111	0	CD16+ Monoc	MS4A7
0	2.85053534	0.986	0.199	0	CD16+ Monoc	SERPINA1
0	2.75347407	0.964	0.112	0	CD16+ Monoc	LILRB2
0	2.7298146	0.977	0.267	0	CD16+ Monoc	FCGR3A
0	2.56590702	0.998	0.683	0	CD16+ Monoc	PSAP
0	2.54645247	0.952	0.124	0	CD16+ Monoc	SMIM25
0	2.40755395	0.996	0.583	0	CD16+ Monoc	COTL1
0	3.78415554	0.959	0.32	0	cDCs	CST3
0	3.48338115	0.996	0.445	0	cDCs	HLA-DRA
0	3.47120225	0.993	0.559	0	cDCs	HLA-DPA1
0	3.3996623	0.991	0.565	0	cDCs	HLA-DPB1
0	3.35217303	0.985	0.283	0	cDCs	HLA-DQA1
0	3.2360881	0.994	0.557	0	cDCs	HLA-DRB1
0	3.09487534	1	0.823	0	cDCs	CD74
0	3.07928523	0.976	0.289	0	cDCs	HLA-DQB1
0	2.89267367	0.964	0.379	0	cDCs	HLA-DRB5
0	2.43802441	0.74	0.117	0	cDCs	CPVL
0	6.34658	0.945	0.006	0	Fibroblasts	DCN
0	6.21548119	0.885	0.014	0	Fibroblasts	MGP
0	5.56495123	0.881	0.012	0	Fibroblasts	COL1A1
0	5.55887332	0.908	0.011	0	Fibroblasts	COL3A1
0	5.45388199	0.973	0.01	0	Fibroblasts	COL1A2
0	5.38569444	0.883	0.005	0	Fibroblasts	FBLN1
0	5.35222315	0.709	0.138	0	Fibroblasts	CFD
0	4.66881787	0.868	0.003	0	Fibroblasts	LUM
0	4.57501489	0.674	0.003	0	Fibroblasts	ADH1B
0	4.47862035	0.696	0.014	0	Fibroblasts	IGFBP6
0	6.52637239	0.97	0.003	0	Mast cells	TPSAB1
0	5.98202428	0.957	0.002	0	Mast cells	TPSB2
0	5.53838749	0.941	0.002	0	Mast cells	CPA3
0	4.20298052	0.856	0.001	0	Mast cells	TPSD1
0	4.09501488	0.887	0.005	0	Mast cells	KIT
0	4.0038518	0.884	0.016	0	Mast cells	SLC24A3
0	3.98141011	0.854	0.007	0	Mast cells	HPGDS
0	3.77256025	0.82	0.001	0	Mast cells	MS4A2
0	3.49847883	0.798	0.007	0	Mast cells	GATA2
0	3.27721607	0.796	0.056	0	Mast cells	VWA5A
0	3.55668656	0.958	0.132	0	pDCs	TCF4
0	3.41674734	0.81	0.033	0	pDCs	PLD4
0	3.25474345	0.855	0.274	0	pDCs	IRF7
0	3.1744021	0.818	0.142	0	pDCs	IRF8
0	2.86415173	0.724	0.008	0	pDCs	LILRA4
0	2.64491553	0.877	0.143	0	pDCs	ITM2C
0	2.6172234	0.822	0.147	0	pDCs	CCDC50
0	2.5089985	0.777	0.044	0	pDCs	SERPINF1
0	2.50778606	0.786	0.04	0	pDCs	RHEX
1.47E-242	3.37203481	0.37	0.085	4.60E-238	pDCs	PTGDS
0	4.92407028	0.765	0.01	0	Endothelial cel	SPARCL1
0	4.24818857	0.887	0.067	0	Endothelial cel	EPAS1
0	4.15006104	0.726	0.009	0	Endothelial cel	VWF
0	3.97699301	0.867	0.015	0	Endothelial cel	LDB2
0	3.85163787	0.882	0.018	0	Endothelial cel	CALCRL
0	3.82799073	0.434	0.003	0	Endothelial cel	FCN3
0	3.81287445	0.86	0.005	0	Endothelial cel	RAMP2
0	3.76289987	0.682	0.005	0	Endothelial cel	CLEC3B
0	3.75942766	0.918	0.028	0	Endothelial cel	EGFL7
0	3.72808728	0.737	0.145	0	Endothelial cel	IGFBP7

Table S2B. Cluster-specific marker gene lists for CD8+ T cells, related to Figure 2.

p_val	avg_log2FC	pct.1	pct.2	p_val_adj	cluster	gene
0	2.47033324	0.826	0.059	0	T8_naive	CCR7
0	2.43867644	0.865	0.132	0	T8_naive	LEF1
0	2.03763506	0.84	0.183	0	T8_naive	SELL
0	1.68211443	0.864	0.425	0	T8_naive	NOSIP
0	1.65376786	0.809	0.327	0	T8_naive	TCF7
0	1.56564544	0.91	0.391	0	T8_naive	LTB
0	1.54627581	0.686	0.155	0	T8_naive	NELL2
0	1.51376579	0.658	0.139	0	T8_naive	BACH2
0	1.48297005	0.576	0.03	0	T8_naive	MAL
0	1.45600485	0.994	0.983	0	T8_naive	RPS5
0	1.65892727	0.896	0.403	0	T8_cm	IL7R
0	1.16958708	0.611	0.177	0	T8_cm	NELL2
0	1.16636601	0.758	0.415	0	T8_cm	LTB
0	0.85527015	0.303	0.044	0	T8_cm	FXYD2
3.409052956	0.99391236	0.692	0.348	1.067988110	T8_cm	TCF7
4.06E-301	0.90041434	0.521	0.172	1.27E-296	T8_cm	LEF1
1.36E-232	0.8139162	0.693	0.379	4.26E-228	T8_cm	PDE3B
1.42E-190	0.77005204	0.624	0.351	4.44E-186	T8_cm	CD55
1.19E-128	0.81991522	0.43	0.223	3.74E-124	T8_cm	SELL
2.45E-82	0.77131323	0.864	0.797	7.67E-78	T8_cm	JUNB
0	1.85168392	0.866	0.338	0	T8_em	GZMK
0	0.86264131	0.767	0.513	0	T8_em	CMC1
0	0.77119313	0.726	0.502	0	T8_em	DUSP2
0	0.65416825	0.98	0.913	0	T8_em	CD74
0	0.61127228	0.428	0.2	0	T8_em	DTHD1
0	0.60310687	0.606	0.386	0	T8_em	SH2D1A
0	0.54809402	0.556	0.328	0	T8_em	EOMES
0	0.50917747	0.46	0.288	0	T8_em	CD27
4.25E-175	0.50798901	0.508	0.371	1.33E-170	T8_em	CCL4L2
0	2.10500504	0.829	0.2	0	T8_emra/eff	FGFBP2
0	1.33087881	0.728	0.221	0	T8_emra/eff	CX3CR1
0	1.2285099	0.964	0.632	0	T8_emra/eff	GZMH
0	1.16431378	0.671	0.212	0	T8_emra/eff	ADGRG1
0	1.1558751	0.535	0.11	0	T8_emra/eff	PRSS23
0	1.02258632	0.842	0.414	0	T8_emra/eff	GNLY
0	1.02127797	0.386	0.082	0	T8_emra/eff	AC243829.2
0	0.99876848	0.897	0.634	0	T8_emra/eff	EFHD2
0	0.9690119	0.99	0.86	0	T8_emra/eff	NKG7
0	0.95820144	0.581	0.253	0	T8_emra/eff	SPON2
0	2.41159476	0.318	0.026	0	T8_NK-like	TRDV1
0	1.79998328	0.59	0.175	0	T8_NK-like	KLRC2
0	1.6301236	0.487	0.138	0	T8_NK-like	TYROBP
0	1.58414459	0.639	0.093	0	T8_NK-like	IKZF2
0	1.24465578	0.382	0.162	0	T8_NK-like	TRGV4
0	1.16588558	0.498	0.116	0	T8_NK-like	KLRF1
0	1.10760681	0.608	0.272	0	T8_NK-like	FCGR3A
0	1.04310166	0.518	0.266	0	T8_NK-like	XCL2
0	0.97428468	0.641	0.315	0	T8_NK-like	KLRB1
0	0.9417987	0.607	0.294	0	T8_NK-like	TIGIT
0	2.13981067	0.677	0.156	0	T8_rm	ZNF683
0	1.97249015	0.532	0.085	0	T8_rm	KLRC1
0	1.72212869	0.255	0.051	0	T8_rm	TRBV27
0	1.59860911	0.293	0.045	0	T8_rm	TRAV3
0	1.49644631	0.724	0.222	0	T8_rm	LGALS3
0	1.41135143	0.66	0.264	0	T8_rm	ITGAE
0	1.35066915	0.644	0.25	0	T8_rm	RBPJ
0	1.29665046	0.872	0.569	0	T8_rm	ALOX5AP
0	1.29029474	0.701	0.395	0	T8_rm	HSPA1A
0	1.28984553	0.486	0.116	0	T8_rm	CAPG
0	3.26333711	0.938	0.26	0	Cycling	STMN1
0	2.8337008	0.967	0.692	0	Cycling	TUBA1B
0	2.6346968	0.771	0.014	0	Cycling	TYMS
0	2.57943966	0.648	0.008	0	Cycling	RRM2
0	2.50789974	0.694	0.024	0	Cycling	MKI67
0	2.50145587	0.918	0.459	0	Cycling	HMGB2
0	2.48682187	0.94	0.64	0	Cycling	TUBB
0	2.38321511	0.807	0.526	0	Cycling	HIST1H4C
0	2.23275066	0.955	0.698	0	Cycling	HMGN2
0	2.13432305	0.526	0.003	0	Cycling	UBE2C
0	2.41601547	0.952	0.393	0	MAIT	IL7R
0	2.17341308	0.926	0.401	0	MAIT	LTB
0	2.02910635	0.789	0.33	0	MAIT	KLRB1
0	1.84939042	0.589	0.011	0	MAIT	SLC4A10
0	1.46506055	0.413	0.067	0	MAIT	TRAV1-2
0	1.42691961	0.39	0.02	0	MAIT	AL136456.1
0	1.4003377	0.684	0.177	0	MAIT	CEBPD
0	1.29575559	0.66	0.16	0	MAIT	AQP3
0	1.13047578	0.708	0.269	0	MAIT	PHACTR2
0	1.0658901	0.498	0.203	0	MAIT	NCR3

Table S2C. Cluster-specific marker gene lists for CD4+ T cells, related to Figure S2.

p_val	avg_log2FC	pct.1	pct.2	p_val_adj	cluster	gene
0	1.45812776	0.452	0.365	0	T4_Tn	RPS10
0	1.45752602	0.785	0.29	0	T4_Tn	CCR7
0	1.22586095	0.857	0.477	0	T4_Tn	LEF1
0	1.15685141	0.573	0.188	0	T4_Tn	TSHZ2
0	1.07667238	0.642	0.256	0	T4_Tn	TXK
0	0.97203306	0.857	0.531	0	T4_Tn	TCF7
0	0.96849933	0.794	0.401	0	T4_Tn	SELL
0	0.9607012	0.754	0.474	0	T4_Tn	PRKCQ-AS1
0	0.95196007	0.648	0.308	0	T4_Tn	BACH2
0	0.94307559	0.881	0.857	0	T4_Tn	RPS20
0	2.5124604	0.689	0.033	0	T4_Treg	IKZF2
0	2.2422772	0.456	0.109	0	T4_Treg	AL136456.1
0	2.13790295	0.612	0.012	0	T4_Treg	FOXP3
0	1.98622424	0.511	0.043	0	T4_Treg	RTKN2
0	1.79257384	0.43	0.112	0	T4_Treg	LINC02694
0	1.7844687	0.621	0.164	0	T4_Treg	CTLA4
0	1.7145146	0.504	0.101	0	T4_Treg	IL2RA
0	1.49167193	0.545	0.101	0	T4_Treg	TIGIT
0	1.43732161	0.563	0.138	0	T4_Treg	STAM
0	1.33820617	0.348	0.01	0	T4_Treg	AC093865.1
0	0.69458479	0.853	0.578	0	T4_Tm	TCF7
0	0.62202148	0.45	0.206	0	T4_Tm	FAAH2
0	0.61353176	0.846	0.664	0	T4_Tm	INPP4B
0	0.58341038	0.644	0.407	0	T4_Tm	ANK3
0	0.51723425	0.585	0.359	0	T4_Tm	LIMS1
0	1.05016706	0.968	0.656	0	T4_Tfh	COTL1
0	0.96755515	0.793	0.374	0	T4_Tfh	LIMS1
3.55E-295	0.77286208	0.658	0.259	1.11E-290	T4_Tfh	SLC9A9
9.56E-287	0.82866518	0.817	0.454	3.00E-282	T4_Tfh	CORO1B
1.58E-276	0.73746645	0.938	0.735	4.96E-272	T4_Tfh	CNN2
2.00E-223	0.74337958	0.853	0.554	6.26E-219	T4_Tfh	ITM2A
7.39E-219	0.8551698	0.957	0.8	2.32E-214	T4_Tfh	CD74
2.76E-198	0.82848622	0.804	0.514	8.66E-194	T4_Tfh	STAT1
1.59E-193	0.75255746	0.859	0.615	4.97E-189	T4_Tfh	ITGA4
9.48E-141	0.75879699	0.594	0.318	2.97E-136	T4_Tfh	RBPJ
0	1.33064192	0.582	0.337	0	T4_Th1	HSPA1A
0	1.25800398	0.507	0.254	0	T4_Th1	HSPA1B
0	1.22616545	0.71	0.499	0	T4_Th1	JUN
0	1.20887632	0.795	0.494	0	T4_Th1	ALOX5AP
0	1.17620833	0.317	0.111	0	T4_Th1	GZMK
0	1.1381947	0.643	0.451	0	T4_Th1	FOS
0	1.11283829	0.567	0.278	0	T4_Th1	RBPJ
0	1.08009368	0.577	0.197	0	T4_Th1	CXCR3
0	1.03107788	0.405	0.134	0	T4_Th1	RGS1
1.98E-177	1.04145137	0.321	0.202	6.21E-173	T4_Th1	CCL4
0	1.05426035	0.558	0.104	0	T4_Th17	CCR6
0	1.01620211	0.983	0.841	0	T4_Th17	IL7R
0	0.93997876	0.963	0.803	0	T4_Th17	LTB
0	0.90707798	0.789	0.431	0	T4_Th17	AQP3
0	0.89293037	0.619	0.302	0	T4_Th17	KLRB1
0	0.79046998	0.462	0.126	0	T4_Th17	PLCB1
0	0.75899695	0.315	0.032	0	T4_Th17	PTPN13
0	0.75022633	0.549	0.224	0	T4_Th17	PDE4D
0	0.69686218	0.407	0.097	0	T4_Th17	AL136456.1
0	0.62340669	0.833	0.608	0	T4_Th17	RORA
0	0.80673451	0.373	0.05	0	T4_Th2	PI16
0	0.76059022	0.51	0.213	0	T4_Th2	CRIP2
0	0.70603639	0.318	0.061	0	T4_Th2	SNED1
0	0.643559	0.628	0.32	0	T4_Th2	C1orf162
0	0.62150325	0.375	0.102	0	T4_Th2	WDR86-AS1
0	0.61397441	0.321	0.083	0	T4_Th2	FXYD7
0	0.6082563	0.982	0.846	0	T4_Th2	IL7R
0	0.58683344	0.987	0.927	0	T4_Th2	FXYD5
0	0.58424853	0.956	0.704	0	T4_Th2	ANXA1
6.04E-300	0.66333201	0.625	0.382	1.89E-295	T4_Th2	GATA3
0	3.38641982	0.979	0.224	0	T4_CTL	NKG7
0	3.28664759	0.891	0.131	0	T4_CTL	GNLY
0	3.27845181	0.971	0.117	0	T4_CTL	GZMH
0	2.70805343	0.829	0.044	0	T4_CTL	FGFBP2
0	2.29941725	0.993	0.354	0	T4_CTL	CCL5
0	2.25344175	0.931	0.252	0	T4_CTL	PRF1
0	2.08661496	0.969	0.328	0	T4_CTL	CST7
0	2.0009302	0.732	0.045	0	T4_CTL	CX3CR1
0	1.83671984	0.779	0.103	0	T4_CTL	GZMB
0	1.80205328	0.781	0.13	0	T4_CTL	ZEB2
0	3.36205136	0.894	0.2	0	Cycling	STMN1
0	3.07091653	0.643	0.159	0	Cycling	GZMB
0	2.83930632	0.946	0.673	0	Cycling	TUBA1B
0	2.51220007	0.666	0.01	0	Cycling	MKI67
0	2.49411521	0.697	0.01	0	Cycling	TYMS
0	2.43890924	0.912	0.621	0	Cycling	TUBB
0	2.42668019	0.61	0.006	0	Cycling	RRM2
0	2.37693858	0.872	0.468	0	Cycling	HMGB2
0	2.37642036	0.566	0.201	0	Cycling	GNLY
0	2.28431424	0.757	0.551	0	Cycling	HIST1H4C

Table S2D. Cluster-specific marker gene lists for B and Plasma cells, related to Figure 5.

p_val	avg_log2FC	pct.1	pct.2	p_val_adj	cluster	gene
0	1.9371869	0.995	0.342	0	B_MZ	TCL1A
0	1.39082337	0.616	0.084	0	B_MZ	AL139020.1
1.22E-299	1.27829181	0.673	0.155	3.83E-295	B_MZ	PCDH9
3.12E-248	1.07455198	0.919	0.524	9.76E-244	B_MZ	NIBAN3
4.36E-243	1.23430491	0.821	0.385	1.37E-238	B_MZ	YBX3
1.87E-218	0.99015753	0.945	0.611	5.86E-214	B_MZ	FCRL1
3.35E-218	0.98263751	0.943	0.559	1.05E-213	B_MZ	BACH2
4.43E-206	1.0080135	0.847	0.44	1.39E-201	B_MZ	CAMK2D
8.69E-187	1.07450058	0.596	0.203	2.72E-182	B_MZ	PLD4
2.57E-111	1.02078935	0.671	0.408	8.05E-107	B_MZ	H1FX
0	2.0833494	0.854	0.134	0	B_naive	TCL1A
0	1.50129584	0.763	0.272	0	B_naive	FCER2
0	1.41981247	0.649	0.187	0	B_naive	IGHD
0	1.3308403	0.538	0.136	0	B_naive	IL4R
0	1.20579044	0.629	0.296	0	B_naive	YBX3
0	1.14738267	0.854	0.514	0	B_naive	FCRL1
0	1.14169124	0.836	0.45	0	B_naive	BACH2
0	1.08689947	0.951	0.829	0	B_naive	BTG1
0	1.07975768	0.615	0.241	0	B_naive	COL19A1
0	1.07798559	0.933	0.826	0	B_naive	TXNIP
0	3.36437362	0.875	0.145	0	B_GC	NR4A2
0	3.28832301	0.889	0.075	0	B_GC	NR4A1
0	3.11080815	0.924	0.305	0	B_GC	CD83
0	3.09909571	0.989	0.659	0	B_GC	JUNB
0	2.44940112	0.705	0.095	0	B_GC	DUSP2
0	2.43053852	0.795	0.246	0	B_GC	GADD45B
0	2.37397413	0.722	0.176	0	B_GC	FOSB
0	2.35077196	0.754	0.213	0	B_GC	SLC2A3
0	2.2385975	0.829	0.259	0	B_GC	DNAJB1
8.18E-253	2.23977912	0.757	0.272	2.56E-248	B_GC	FOS
0	2.74350617	0.969	0.677	0	B_pre_mem	AFF3
0	2.73286017	0.911	0.572	0	B_pre_mem	BACH2
0	2.5720176	0.739	0.268	0	B_pre_mem	AUTS2
0	2.53240822	0.824	0.402	0	B_pre_mem	MGAT5
0	2.42152374	0.938	0.611	0	B_pre_mem	FOXP1
0	2.40896944	0.742	0.288	0	B_pre_mem	PRKCE
0	2.39467255	0.826	0.459	0	B_pre_mem	EBF1
0	2.29625529	0.96	0.795	0	B_pre_mem	ARHGAP15
0	2.2948582	0.697	0.373	0	B_pre_mem	MAML2
5.49E-240	2.25129285	0.61	0.286	1.72E-235	B_pre_mem	CCSER1
0	1.54045485	0.483	0.098	0	B_mem.1	LINC01781
0	1.22619669	0.831	0.481	0	B_mem.1	CRIP1
0	1.19390652	0.622	0.18	0	B_mem.1	AIM2
0	1.08818588	0.591	0.343	0	B_mem.1	S100A4
0	1.08804329	0.591	0.361	0	B_mem.1	S100A10
0	0.99995974	0.97	0.64	0	B_mem.1	LTB
0	0.85704195	0.739	0.481	0	B_mem.1	S100A6
0	0.84587723	0.825	0.523	0	B_mem.1	COTL1
0	0.83452317	0.366	0.098	0	B_mem.1	COPDA1
0	0.83221845	0.555	0.275	0	B_mem.1	MARCKS
0	2.47470799	0.747	0.093	0	B_mem.2	SOX5
0	1.84579638	0.789	0.139	0	B_mem.2	FGR
0	1.66576483	0.846	0.342	0	B_mem.2	ZEB2
0	1.58507356	0.72	0.18	0	B_mem.2	FCRL3
0	1.58477883	0.869	0.496	0	B_mem.2	CIB1
0	1.56002329	0.928	0.552	0	B_mem.2	CRIP1
0	1.26487858	0.659	0.108	0	B_mem.2	HCK
0.00E+00	1.26024151	0.389	0.051	1.18E-304	B_mem.2	SYT1
3.82E-295	1.37844999	0.701	0.369	1.20E-290	B_mem.2	FCRL5
1.52E-226	1.27802606	0.491	0.137	4.75E-222	B_mem.2	PPP1R14A
0	3.81261744	0.789	0.27	0	PB	IGHG1
0	3.1000302	0.492	0.094	0	PB	IGHG4
0	2.988052	0.662	0.19	0	PB	HSPA1A
0	2.47901177	0.614	0.162	0	PB	HSPA1B
0	2.28966656	0.755	0.013	0	PB	RRM2
1.60E-306	2.79448096	0.643	0.198	5.02E-302	PB	IGLC1
7.66E-260	3.5145442	0.516	0.209	2.40E-255	PB	IGHG3
1.06E-192	2.92619947	0.466	0.306	3.33E-188	PB	IGLC2
1.59E-155	2.73847145	0.305	0.07	4.97E-151	PB	IGLV6-57
4.69E-104	2.57420484	0.264	0.075	1.47E-99	PB	IGLV3-21
0	3.42628712	0.681	0.518	0	PC.1	IGKC
0	3.41852372	0.998	0.506	0	PC.1	JCHAIN
0	2.76799848	0.993	0.33	0	PC.1	TXNDC5
0	2.69559974	0.777	0.392	0	PC.1	IGHA1
0	2.59291582	0.998	0.392	0	PC.1	MZB1
0	2.55343523	1	0.639	0	PC.1	HSP90B1
0	2.38056903	1	0.699	0	PC.1	PPIB
3.31E-270	3.31002058	0.525	0.179	1.04E-265	PC.1	IGHA2
3.03E-156	3.67788377	0.466	0.177	9.48E-152	PC.1	IGKV4-1
4.98E-48	2.94167348	0.274	0.168	1.56E-43	PC.1	IGHV3-23
1.83E-264	2.68275628	0.751	0.293	5.72E-260	PC.2	IGHG1
1.90E-179	2.60357087	0.603	0.307	5.96E-175	PC.2	IGLC2
6.85E-178	4.47998554	0.437	0.074	2.15E-173	PC.2	IGLV6-57
1.19E-156	4.48348199	0.474	0.097	3.71E-152	PC.2	IGLV3-1
4.20E-69	2.68107263	0.286	0.08	1.32E-64	PC.2	IGLV10-54
2.23E-55	3.08899168	0.383	0.151	6.98E-51	PC.2	IGLV2-14
6.95E-38	3.0251209	0.266	0.101	2.18E-33	PC.2	IGLV1-40
1.08E-30	2.74568046	0.261	0.11	3.38E-26	PC.2	IGHV4-39
9.93E-22	3.16928361	0.346	0.196	3.11E-17	PC.2	IGKV3-20
1.15E-19	3.21777674	0.278	0.15	3.60E-15	PC.2	IGLV1-44
0	4.10423695	0.968	0.223	0	PC.3	FNDC3B
0	3.40721112	0.962	0.404	0	PC.3	FBXW7
0	3.3232802	0.94	0.508	0	PC.3	NEAT1
0	3.20325326	0.892	0.281	0	PC.3	MAN1A1
0	3.06876148	0.898	0.238	0	PC.3	ATXN1
0	3.05017593	0.937	0.413	0	PC.3	GLCCI1
0	3.00730134	0.905	0.386	0	PC.3	IFNG-AS1
0	2.92221169	0.959	0.498	0	PC.3	MED13L
1.44E-155	3.40256963	0.502	0.096	4.50E-151	PC.3	DCC
3.56E-43	2.89697623	0.584	0.424	1.11E-38	PC.3	IGHA1

Table S2E. Cluster-specific marker gene lists for monocytes and macrophages, related to Figure 6.

p_val	avg_log2FC	pct.1	pct.2	p_val_adj	cluster	gene
0	2.34178625	0.843	0.315	0	Mono_CD14	S100A12
0	2.30645941	0.969	0.759	0	Mono_CD14	S100A8
0	2.09739084	0.971	0.865	0	Mono_CD14	S100A9
0	1.56318031	0.903	0.579	0	Mono_CD14	VCAN
0	1.36024902	0.976	0.917	0	Mono_CD14	LYZ
0	1.27146353	0.896	0.534	0	Mono_CD14	FCN1
0	1.20032181	0.647	0.459	0	Mono_CD14	PLBD1
0	1.18398897	0.889	0.766	0	Mono_CD14	MNDA
0	1.13622945	0.584	0.239	0	Mono_CD14	SELL
0	1.13400986	0.483	0.151	0	Mono_CD14	PLCB1
0	0.70913795	0.955	0.857	0	Mono_CD14	RPL13A
0	0.70704293	0.984	0.934	0	Mono_CD14	RPS2
0	0.70684028	0.943	0.812	0	Mono_CD14	RPL10A
0	0.68210134	0.97	0.896	0	Mono_CD14	RPL37A
0	0.67928392	0.984	0.913	0	Mono_CD14	RPS6
0	0.66965357	0.956	0.856	0	Mono_CD14	RPL23A
0	0.66880111	0.959	0.852	0	Mono_CD14	RPL5
0	0.66196129	0.964	0.87	0	Mono_CD14	RPL21
3.96E-302	0.69212625	0.944	0.826	1.24E-297	Mono_CD14	RPS29
5.28E-118	0.6613	0.52	0.359	1.65E-113	Mono_CD14	RPS10
0	3.09420822	0.801	0.072	0	Mono_CD16	CDKN1C
0	2.24437884	0.961	0.493	0	Mono_CD16	FCGR3A
0	2.22359173	0.316	0.006	0	Mono_CD16	LYPD2
0	2.17067712	0.996	0.816	0	Mono_CD16	LST1
0	1.90736193	0.89	0.32	0	Mono_CD16	RHOC
0	1.81517435	0.888	0.36	0	Mono_CD16	RHOB
0	1.69673116	0.931	0.37	0	Mono_CD16	MTSS1
0	1.68667652	0.968	0.576	0	Mono_CD16	LILRB2
0	1.63396422	0.999	0.88	0	Mono_CD16	COTL1
0	1.60211861	0.741	0.31	0	Mono_CD16	NR4A1
0	2.53425273	0.605	0.193	0	transitioning	CCL2
0	2.42811545	0.349	0.079	0	transitioning	CXCL10
0	1.86883316	0.514	0.242	0	transitioning	IFI27
0	1.79938094	0.364	0.185	0	transitioning	CCL4
0	1.78979248	0.745	0.48	0	transitioning	ISG15
0	1.7688975	0.562	0.153	0	transitioning	C15orf48
0	1.6646133	0.617	0.261	0	transitioning	IFITM1
0	1.57471118	0.505	0.225	0	transitioning	IL1RN
0	1.51502398	0.314	0.133	0	transitioning	SPP1
0	1.38661353	0.633	0.331	0	transitioning	GBP1
0	1.96725208	0.578	0.406	0	transitioning	FMNL2
0	1.96210948	0.694	0.487	0	transitioning	FMN1
0	1.9518605	0.455	0.237	0	transitioning	KCNMA1
0	1.85859133	0.888	0.77	0	transitioning	LRMDA
0	1.69247291	0.696	0.528	0	transitioning	DOCK4
0	1.68292883	0.821	0.7	0	transitioning	CHST11
0	1.60568879	0.523	0.327	0	transitioning	FHIT
0	1.60098854	0.589	0.433	0	transitioning	FNIP2
0	1.59885357	0.583	0.494	0	transitioning	ALCAM
0	1.58892159	0.521	0.339	0	transitioning	MITF
0	2.54846826	0.774	0.256	0	MDM_CCL18	APOE
0	2.46883461	0.51	0.141	0	MDM_CCL18	CCL18
0	2.1042239	0.879	0.285	0	MDM_CCL18	C1QC
0	2.07154507	0.721	0.177	0	MDM_CCL18	PLTP
0	2.06263075	0.69	0.272	0	MDM_CCL18	RNASE1
0	2.06249624	0.8	0.277	0	MDM_CCL18	APOC1
0	1.98798096	0.865	0.288	0	MDM_CCL18	C1QB
0	1.96474023	0.803	0.27	0	MDM_CCL18	GPNMB
0	1.91688582	0.347	0.054	0	MDM_CCL18	SELENOP
0	1.87280923	0.875	0.321	0	MDM_CCL18	C1QA
0	4.17938605	0.837	0.027	0	AM_FABP4	FABP4
0	2.64267096	0.828	0.09	0	AM_FABP4	LSAMP
0	2.40024732	0.999	0.323	0	AM_FABP4	APOC1
0	2.30723547	0.966	0.48	0	AM_FABP4	FBP1
0	2.287798	0.994	0.374	0	AM_FABP4	C1QA
0	2.28711965	0.988	0.384	0	AM_FABP4	MARCO
0	2.2617511	0.991	0.343	0	AM_FABP4	C1QB
0	2.10258671	0.833	0.165	0	AM_FABP4	CCL18
0	2.07852166	0.8	0.19	0	AM_FABP4	FN1
0	2.01854887	0.876	0.384	0	AM_FABP4	MCEMP1

Table S2F. Cluster-specific marker gene lists for epithelial cells, related to Figure 6.

p_val	avg_log2FC	pct.1	pct.2	p_val_adj	cluster	gene
0	3.44241185	0.809	0.17	0	AT1	CAV1
0	2.98817882	0.91	0.615	0	AT1	EMP2
0	2.66591408	0.938	0.432	0	AT1	KRT7
0	2.38968679	0.945	0.409	0	AT1	GPRC5A
0	2.21809722	0.978	0.761	0	AT1	S100A10
1.20E-284	3.75166272	0.477	0.124	3.77E-280	AT1	AGER
2.78E-277	2.18365021	0.843	0.337	8.70E-273	AT1	CEACAM6
5.16E-233	2.92810992	0.438	0.051	1.62E-228	AT1	RTKN2
8.57E-133	2.54134282	0.32	0.084	2.69E-128	AT1	NCKAP5
2.89E-100	2.38770534	0.502	0.16	9.05E-96	AT1	S100A2
0	4.62045914	0.98	0.285	0	AT2	SFTPC
0	3.45895402	0.988	0.225	0	AT2	SFTPA1
0	3.33443161	0.914	0.094	0	AT2	PGC
0	3.10459609	0.987	0.228	0	AT2	SFTPA2
0	2.75317319	0.996	0.2	0	AT2	NAPSA
0	2.64946231	0.988	0.213	0	AT2	SFTA2
0	2.62698795	0.976	0.156	0	AT2	SFTPD
0	2.29678174	1	0.323	0	AT2	SFTPB
0	2.29481869	0.995	0.663	0	AT2	NPC2
0	2.2451574	0.358	0.032	0	AT2	ATP13A4-AS1
0	2.23203059	0.572	0.509	0	EMT_Epi	VIM
0	1.30176505	0.361	0.502	0	EMT_Epi	IFI30
0	1.25590906	0.546	0.598	0	EMT_Epi	IFITM2
0	1.24638741	0.436	0.515	0	EMT_Epi	S100A4
0	1.22592372	0.732	0.817	0	EMT_Epi	IFITM3
5.29E-294	1.08999832	0.872	0.917	1.66E-289	EMT_Epi	TMSB10
4.42E-291	1.89220836	0.4	0.295	1.39E-286	EMT_Epi	LGALS1
1.80E-224	1.46262859	0.331	0.42	5.65E-220	EMT_Epi	TIMP1
6.74E-217	1.4211282	0.282	0.15	2.11E-212	EMT_Epi	SRGN
4.01E-203	1.45360881	0.289	0.134	1.25E-198	EMT_Epi	TYROBP
0	3.66594713	0.957	0.237	0	Ciliated cells	CAPS
0	3.56929274	0.931	0.082	0	Ciliated cells	C20orf85
0	3.36029501	0.909	0.086	0	Ciliated cells	C9orf24
0	3.17203472	0.898	0.128	0	Ciliated cells	DNAH12
0	3.12473228	0.878	0.095	0	Ciliated cells	CFAP299
0	3.02917495	0.938	0.101	0	Ciliated cells	RSPH1
0	3.02124985	0.883	0.058	0	Ciliated cells	C1orf194
0	3.0122528	0.864	0.155	0	Ciliated cells	RP1
0	2.98022558	0.883	0.146	0	Ciliated cells	HYDIN
0	2.97513852	0.891	0.115	0	Ciliated cells	LRRIQ1
0	2.00987416	0.762	0.145	0	Tuft cells	AQP5
4.37E-217	2.01608301	0.973	0.796	1.37E-212	Tuft cells	WFDC2
1.79E-212	3.04853327	0.527	0.108	5.61E-208	Tuft cells	SERPINB3
1.89E-208	3.26657919	0.771	0.36	5.93E-204	Tuft cells	SCGB3A1
6.98E-205	3.54278638	0.701	0.279	2.19E-200	Tuft cells	SCGB1A1
4.53E-174	2.11521672	0.728	0.355	1.42E-169	Tuft cells	LCN2
5.58E-159	3.06240342	0.522	0.183	1.75E-154	Tuft cells	BPIFB1
1.43E-134	2.74705939	0.384	0.07	4.47E-130	Tuft cells	MSMB
3.51E-125	3.31013844	0.351	0.179	1.10E-120	Tuft cells	SCGB3A2
2.12E-72	2.49163889	0.255	0.055	6.63E-68	Tuft cells	BPIFA1
0	2.84030045	0.954	0.398	0	Club cells	ATP11A
0	2.77846441	0.981	0.667	0	Club cells	MECOM
2.93E-300	2.75987885	0.946	0.725	9.19E-296	Club cells	PDE4D
9.38E-291	2.56421862	0.864	0.55	2.94E-286	Club cells	AUTS2
2.39E-255	3.06967954	0.859	0.267	7.48E-251	Club cells	ZNF385B
8.38E-236	2.56881853	0.823	0.239	2.62E-231	Club cells	ACOXL
9.33E-225	2.64789745	0.739	0.208	2.92E-220	Club cells	AFF3
4.06E-215	2.70991516	0.829	0.309	1.27E-210	Club cells	DLG2
2.10E-200	2.71906612	0.804	0.553	6.59E-196	Club cells	MACROD2
1.47E-150	2.73513598	0.832	0.321	4.60E-146	Club cells	LRRK2
2.00E-259	2.45019971	0.811	0.322	6.27E-255	Goblet cells	BMPR1B
1.29E-206	3.0494337	0.605	0.091	4.04E-202	Goblet cells	RIMS1
2.18E-199	1.94401578	0.745	0.443	6.83E-195	Goblet cells	GMDS
1.11E-188	1.94086607	0.929	0.669	3.47E-184	Goblet cells	MECOM
2.89E-131	2.32091114	0.636	0.305	9.05E-127	Goblet cells	SLC4A4
1.02E-117	2.10181982	0.636	0.321	3.21E-113	Goblet cells	ANKRD36C
4.39E-117	2.00404821	0.485	0.245	1.38E-112	Goblet cells	TSHZ2
1.42E-107	1.99018304	0.836	0.474	4.44E-103	Goblet cells	SDK1
6.65E-98	1.95344375	0.595	0.293	2.08E-93	Goblet cells	LINC00342
9.36E-28	2.28528723	0.351	0.187	2.93E-23	Goblet cells	SCGB3A2
0	4.73580973	0.974	0.393	0	Mesothelial	TIMP1
8.46E-261	3.39230448	0.894	0.33	2.65E-256	Mesothelial	CFB
1.77E-253	4.57214699	0.783	0.007	5.55E-249	Mesothelial	PRG4
8.32E-231	5.19010584	0.741	0.008	2.61E-226	Mesothelial	ITLN1
1.48E-224	4.03646552	0.767	0.01	4.65E-220	Mesothelial	PLA2G2A
4.75E-211	3.17806393	0.899	0.259	1.49E-206	Mesothelial	CCDC80
1.53E-207	3.33760839	0.921	0.434	4.80E-203	Mesothelial	C3
5.68E-203	5.26244976	0.783	0.051	1.78E-198	Mesothelial	HP
3.00E-192	3.14166353	0.815	0.077	9.39E-188	Mesothelial	UPK3B
4.16E-168	3.17692834	0.878	0.225	1.30E-163	Mesothelial	RARRES1

Table S2G. Genes for monocytes and macrophages module score, related to Figure 6.

pro-inflammatory	anti-inflammatory	monocytes
IL1B	CD163	LYZ
IL6	MRC1	S100A8
CCL2	MSR1	CD14
CCL3	CCL13	VCAN
CCL4	CCL18	FCN1
CCL7	IL10	S100A9
CCL23	FOLR2	
TNF	STAB1	
CXCL2	CD163L1	
CXCL9	PLD4	
CXCL10	F13A1	
SOCS3	MERTK	
HSPH1	AXL	
HSPB1	CCL22	
HSPA1B	GPNMB	
HSPA1A	CHI3L1	

454

Table S2. Top 10 cluster-specific marker gene lists for each cell subset and genes for macrophages and monocytes module score, related to Figure 1, 2, 5, 6 and S2.

(A) Table S2A. Cluster-specific marker gene lists for all major cell types, related to Figure 1.
(B) Table S2B. Cluster-specific marker gene lists for CD8+ T cells, related to Figure 2.
(C) Table S2C. Cluster-specific marker gene lists for CD4+ T cells, related to Figure S2.
(D) Table S2D. Cluster-specific marker gene lists for B and Plasma cells, related to Figure 5.
(E) Table S2E. Cluster-specific marker gene lists for monocytes and macrophages, related to Figure 6.
(F) Table S2F. Cluster-specific marker gene lists for epithelial cells, related to Figure 6.
(G) Table S2G. Genes for monocytes and macrophages module score, related to Figure6.

Table S3A. Top 30 Differentially expressed genes between pneumonia and control T8_rm, related to Figure 2.

	p_val	avg_log2FC	pct.1	pct.2	p_val_adj
KLF2	1.20E-227	-1.996176998	0.153	0.617	3.77E-223
GZMA	2.68E-218	1.42491545	0.926	0.642	8.38E-214
RBFOX2	1.89E-213	-1.120979104	0.734	0.923	5.93E-209
TCF7	2.15E-178	-1.243096646	0.065	0.438	6.74E-174
KLF6	9.51E-172	-1.246736998	0.682	0.889	2.98E-167
MYBL1	1.43E-161	-1.02241098	0.043	0.355	4.47E-157
CXCR6	2.63E-157	1.509170398	0.59	0.167	8.25E-153
CLIC3	6.49E-149	1.521331227	0.636	0.211	2.03E-144
HLA-DRA	2.75E-146	1.376837606	0.731	0.33	8.61E-142
LMNA	1.44E-143	-1.178881157	0.06	0.363	4.52E-139
COTL1	5.50E-143	1.015959335	0.89	0.67	1.72E-138
GZMB	5.33E-139	2.076904029	0.749	0.377	1.67E-134
CD3D	1.63E-134	0.774454185	0.948	0.85	5.12E-130
CCL5	2.19E-129	1.032081892	0.985	0.953	6.86E-125
GAPDH	4.16E-127	0.964640091	0.983	0.95	1.30E-122
CD2	4.85E-127	0.776373448	0.92	0.837	1.52E-122
ALOX5AP	1.80E-126	1.037205723	0.874	0.687	5.64E-122
EEF1A1	2.66E-123	-0.660966709	0.995	0.998	8.35E-119
IKZF2	1.46E-121	-0.986844571	0.028	0.269	4.58E-117
CD38	6.38E-121	1.198467136	0.351	0.032	2.00E-116
IL7R	1.61E-120	-1.418756106	0.248	0.614	5.05E-116
FP236383.3	3.34E-119	-0.905838091	0.975	0.999	1.05E-114
JUNB	3.88E-118	-1.252204025	0.825	0.88	1.22E-113
TXNIP	8.81E-113	-1.046634067	0.742	0.89	2.76E-108
NR4A2	2.88E-110	-1.221740353	0.391	0.669	9.02E-106
ZFP36L2	1.36E-109	-0.998422655	0.851	0.957	4.27E-105
MT-CYB	2.41E-109	-0.731492358	0.929	0.995	7.55E-105
IFI27	3.17E-109	2.890754284	0.291	0.016	9.92E-105
ITGAE	1.22E-108	1.155777114	0.66	0.335	3.82E-104
AHNAK	6.29E-108	-0.941739148	0.645	0.855	1.97E-103

Table S3B. Top 30 Differentially expressed genes between TCR pattern 1a and pattern 1b, related to Figure S3.

	p_val	avg_log2FC	pct.1	pct.2	p_val_adj
HLA-C	9.71E-182	-0.846856785	0.988	0.998	3.04E-177
IFI27	4.52E-167	3.174816906	0.423	0.062	1.42E-162
GZMB	2.95E-103	1.342398786	0.841	0.665	9.25E-99
LGALS1	1.61E-86	1.678888097	0.643	0.465	5.05E-82
MT-CO3	1.10E-83	-0.625866739	0.942	0.977	3.44E-79
IFI44L	2.97E-83	0.709950765	0.34	0.071	9.31E-79
TRBV10-2	3.15E-82	2.222039787	0.18	0.001	9.88E-78
MT-CYB	1.20E-79	-0.602774053	0.952	0.986	3.76E-75
MX1	4.54E-77	0.990431726	0.476	0.206	1.42E-72
TRAV21	3.31E-74	1.581835478	0.131	0.071	1.04E-69
SH3BGRL3	1.88E-72	0.535697016	0.998	0.995	5.89E-68
ISG15	2.18E-72	1.027308499	0.616	0.404	6.84E-68
PCLAF	3.23E-71	0.91513782	0.269	0.048	1.01E-66
GAPDH	2.38E-70	0.822668888	0.995	0.982	7.45E-66
TRBV5-6	1.45E-67	2.004467764	0.19	0.015	4.55E-63
CCL5	1.06E-66	0.524863707	0.999	0.997	3.33E-62
DDX5	1.92E-65	-0.47105525	0.938	0.96	6.03E-61
KLRG1	2.74E-65	-0.818946628	0.154	0.349	8.60E-61
TYMS	7.59E-65	1.172632649	0.25	0.051	2.38E-60
S100A11	2.95E-64	0.789558344	0.85	0.79	9.25E-60
RPL21	8.11E-64	-0.417032505	0.944	0.977	2.54E-59
HMGN2	2.71E-63	1.083633833	0.793	0.702	8.50E-59
TRAV3	3.94E-63	1.467512142	0.196	0.028	1.23E-58
RPS4Y1	3.22E-62	-0.85226857	0.206	0.465	1.01E-57
MT-ND1	6.28E-61	-0.553998965	0.892	0.953	1.97E-56
CXCR4	1.34E-60	-0.622964679	0.537	0.788	4.21E-56
TRAV1-1	2.37E-59	-0.886627668	0.006	0.109	7.43E-55
RPS2	8.72E-59	-0.399281489	0.972	0.985	2.73E-54
LY6E	1.77E-58	0.644957408	0.864	0.786	5.55E-54
OAS1	5.38E-58	0.551287804	0.34	0.108	1.69E-53

Table S3C. Top 30 Differentially expressed genes between TCR pattern 1 and pattern 2, related to Figure S3.

	p_val	avg_log2FC	pct.1	pct.2	p_val_adj
CAMK4	0	0.963664041	0.647	0.189	0
CMC1	0	-1.710017794	0.379	0.638	0
COTL1	0	1.569939429	0.906	0.367	0
CST7	0	-1.447130009	0.9	0.954	0
CX3CR1	0	-1.620112164	0.051	0.467	0
CXCR3	0	1.187219485	0.677	0.191	0
FGFBP2	0	-1.94840094	0.089	0.48	0
GZMM	0	-1.095444546	0.654	0.814	0
KLF2	0	-1.752593038	0.195	0.621	0
KLRG1	0	-1.557941409	0.193	0.628	0
LGALS3	0	1.300884119	0.72	0.2	0
NKG7	0	-1.317173571	0.938	0.959	0
PLAC8	0	-1.731359782	0.054	0.478	0
PLEK	0	-1.516355179	0.235	0.617	0
S1PR5	0	-1.39480741	0.038	0.432	0
TRGV4	0	-2.258093391	0.053	0.314	0
CXCR6	7.2326175626974[illegible]	1.241680781	0.6	0.16	2.26583443004187e-310
CCL5	3.4508266277985[illegible]	0.866720299	0.999	0.992	1.08E-306
CD52	7.56E-305	0.785356888	0.972	0.725	2.37E-300
HLA-DRA	4.88E-292	1.075200117	0.804	0.395	1.53E-287
ZNF683	6.92E-288	1.087760509	0.602	0.178	2.17E-283
KLRC1	4.87E-281	1.374586683	0.459	0.078	1.52E-276
FCGR3A	2.67E-278	-1.493475519	0.133	0.441	8.37E-274
RPL3	3.31E-272	-0.647711158	0.988	0.988	1.04E-267
TRBV28	6.77E-272	-2.251429706	0.018	0.252	2.12E-267
CEBPD	9.99E-260	1.252953995	0.448	0.084	3.13E-255
JAML	2.92E-259	0.898352149	0.499	0.117	9.15E-255
ITGA1	1.35E-258	1.01055641	0.448	0.083	4.22E-254
FGR	1.15E-257	-1.049279239	0.07	0.373	3.60E-253
TXNIP	4.13E-254	-1.087832341	0.729	0.813	1.29E-249

Table S3D. Top 30 Differentially expressed genes between P6 cells with and without Influenza B virus reads, related to Figure S4.

	p_val	avg_log2FC	pct.1	pct.2	p_val_adj
RPS18	8.31E-13	-1.871222716	0.897	0.968	2.60E-08
HCAR3	1.58E-12	2.233611792	0.241	0.006	4.95E-08
IL1RN	2.36E-12	2.629814694	0.621	0.135	7.40E-08
METRNL	3.83E-11	1.222304396	0.793	0.209	1.20E-06
RPS19	6.88E-11	-1.53952716	0.897	0.981	2.16E-06
PLAUR	2.16E-10	1.959342987	0.655	0.266	6.77E-06
RBM47	8.34E-10	1.556092262	0.828	0.338	2.61E-05
RPS28	9.69E-10	-1.769848517	0.966	0.971	3.03E-05
RPL32	9.71E-10	-1.829603339	0.931	0.968	3.04E-05
RPL18A	1.84E-09	-1.544482003	0.897	0.956	5.77E-05
RPS27	2.02E-09	-2.165158837	0.828	0.957	6.31E-05
RPS23	2.28E-09	-1.710438252	0.862	0.962	7.15E-05
SOD2	2.42E-09	2.121963156	0.862	0.433	7.59E-05
RPL35	2.94E-09	-1.113641669	0.931	0.938	9.21E-05
ICAM1	2.96E-09	1.373283739	0.69	0.203	9.26E-05
B4GALT5	3.02E-09	1.652463885	0.69	0.206	9.46E-05
MAP4	3.85E-09	0.465309827	0.862	0.328	0.000120707
GBP1	4.39E-09	1.546810759	0.862	0.383	0.000137463
RPL36	6.73E-09	-1.701164378	0.897	0.958	0.000210906
RPL14	8.78E-09	-1.48134457	0.862	0.935	0.000275121
CCRL2	1.11E-08	1.496930416	0.414	0.085	0.000346665
NFKB1	1.44E-08	0.77926301	0.862	0.324	0.000452523
RPL7A	1.82E-08	-1.499159052	0.862	0.948	0.000571049
CSF1	2.27E-08	2.061532746	0.448	0.081	0.000711026
IRF1	2.68E-08	1.68213576	0.862	0.504	0.000838566
HES4	2.70E-08	2.31841496	0.276	0.041	0.000846718
NEAT1	3.19E-08	1.689767072	0.931	0.582	0.000999496
ARHGAP26	3.34E-08	1.053572791	0.897	0.377	0.001046729
RPL39	3.71E-08	-1.897218333	0.862	0.958	0.001161358
RPL26	4.06E-08	-1.392794304	0.931	0.954	0.001273189

Table S3E. Top 30 Differentially expressed genes between pneumonia and control PB and PC, related to Figure S5.

	p_val	avg_log2FC	pct.1	pct.2	p_val_adj
GSTM1	5.20E-48	-0.26575462	0.006	0.331	1.63E-43
SPINK2	5.27E-46	-0.546873312	0.014	0.355	1.65E-41
SPCS3	2.40E-42	0.819113106	0.91	0.975	7.51E-38
AMPD1	1.73E-40	-0.628646356	0.031	0.397	5.43E-36
IFI30	2.62E-40	1.523116804	0.874	0.496	8.22E-36
ZDHHC14	9.64E-39	-0.56018518	0.025	0.388	3.02E-34
SMOC1	1.54E-37	-0.305831083	0.012	0.314	4.83E-33
TIMP1	1.57E-37	-0.943205254	0.119	0.579	4.93E-33
NRG3	3.16E-37	-0.750008128	0.001	0.215	9.89E-33
BCL2	6.12E-37	-1.024785992	0.311	0.868	1.92E-32
SERPINB6	2.36E-36	-0.295041314	0.021	0.355	7.40E-32
CTSW	5.17E-36	-0.994465398	0.037	0.306	1.62E-31
CADPS2	3.10E-34	-1.009624484	0.044	0.421	9.70E-30
LMNA	5.97E-34	-0.859191158	0.158	0.603	1.87E-29
IL16	9.88E-33	-0.630660347	0.228	0.719	3.10E-28
DUSP1	2.97E-32	-1.824296278	0.337	0.554	9.29E-28
TNFRSF4	1.41E-31	-0.327249919	0.034	0.372	4.43E-27
SLC22A17	1.45E-31	-0.275199158	0.004	0.215	4.55E-27
SKAP1	5.59E-31	-0.759467855	0.112	0.554	1.75E-26
AL589693.1	6.57E-31	-1.65325126	0.372	0.818	2.06E-26
FOXP2	1.26E-30	-0.280622557	0.002	0.198	3.94E-26
KLF6	8.04E-30	-1.361218883	0.418	0.752	2.52E-25
MLLT3	1.82E-29	-0.678113935	0.122	0.545	5.71E-25
PSME2	2.48E-29	1.220098541	0.881	0.686	7.77E-25
KLF2	3.33E-29	-1.169600606	0.521	0.893	1.04E-24
ST3GAL6	4.08E-29	-0.368978516	0.011	0.256	1.28E-24
ZFP36L2	7.65E-29	-0.954394904	0.464	0.884	2.40E-24
BTG1	2.46E-28	-0.951608542	0.61	0.926	7.71E-24
SPINT2	4.26E-28	-0.448186261	0.159	0.612	1.34E-23
FBP1	6.88E-28	-0.335543515	0.018	0.281	2.16E-23

Table S3. Differentially expressed genes between pneumonia and control T8_rm, pattern 1a and pattern 1b, pattern 1 and pattern 2, P6 cells with and without Influenza B virus reads, and pneumonia and control PB and PC, related to Figure 2, S3, S4 and S5.

(A) Table S3A. Differentially expressed genes between pneumonia and control T8_rm, related to Figure 2.
(B) Table S3B. Differentially expressed genes between TCR pattern 1a and pattern 1b, related to Figure S3.
(C) Table S3C. Differentially expressed genes between TCR pattern 1 and pattern 2, related to Figure S3.
(D) Table S3D. Differentially expressed genes between P6 cells with and without Influenza B virus reads, related to Figure S4.
(E) Table S3E. Differentially expressed genes between pneumonia and control PB and PC, related to Figure S5.

Table S4A. Top 30 GO enrichment analysis in biological process between pneumonia and control T8_rm, related to Figure S2

ID	Description	GeneRatio	BgRatio	pvalue	p.adjust	qvalue	geneID	Count	direction
GO:0002495	antigen processing and presentation of peptide antigen via MHC class II	12/143	34/18800	1.11E-17	2.69E-14	2.05E-14	HLA-DRA/CD74/HI	12	up
GO:0002504	antigen processing and presentation of peptide or polysaccharide antigen via MHC class II	12/143	36/18800	2.51E-17	3.03E-14	2.31E-14	HLA-DRA/CD74/HI	12	up
GO:0019886	antigen processing and presentation of exogenous peptide antigen via MHC class II	11/143	30/18800	1.61E-16	1.30E-13	9.89E-14	HLA-DRA/CD74/HI	11	up
GO:0002478	antigen processing and presentation of exogenous peptide antigen	11/143	38/18800	3.37E-15	2.04E-12	1.55E-12	HLA-DRA/CD74/HI	11	up
GO:0048002	antigen processing and presentation of peptide antigen	12/143	63/18800	4.50E-14	1.85E-11	1.41E-11	HLA-DRA/CD74/HI	12	up
GO:0019884	antigen processing and presentation of exogenous antigen	11/143	47/18800	4.60E-14	1.85E-11	1.41E-11	HLA-DRA/CD74/HI	11	up
GO:0002399	MHC class II protein complex assembly	8/143	16/18800	1.12E-13	3.39E-11	2.59E-11	HLA-DRA/HLA-DR	8	up
GO:0002503	peptide antigen assembly with MHC class II protein complex	8/143	16/18800	1.12E-13	3.39E-11	2.59E-11	HLA-DRA/HLA-DR	8	up
GO:0002449	lymphocyte mediated immunity	21/143	365/18800	5.61E-13	1.51E-10	1.15E-10	HLA-DRA/GZMB/(	21	up
GO:0002443	leukocyte mediated immunity	23/143	457/18800	6.52E-13	1.57E-10	1.20E-10	HLA-DRA/GZMB/(	23	up
GO:0016032	viral process	22/143	418/18800	8.93E-13	1.85E-10	1.41E-10	CXCR6/CCL5/IFI27/	22	up
GO:0002696	positive regulation of leukocyte activation	22/143	421/18800	1.03E-12	1.85E-10	1.41E-10	HLA-DRA/CCL5/CI	22	up
GO:0002396	MHC protein complex assembly	8/143	20/18800	1.07E-12	1.85E-10	1.41E-10	HLA-DRA/HLA-DR	8	up
GO:0002501	peptide antigen assembly with MHC protein complex	8/143	20/18800	1.07E-12	1.85E-10	1.41E-10	HLA-DRA/HLA-DR	8	up
GO:0050863	regulation of T cell activation	20/143	342/18800	1.55E-12	2.44E-10	1.86E-10	HLA-DRA/CCL5/CI	20	up
GO:0019882	antigen processing and presentation	13/143	107/18800	1.61E-12	2.44E-10	1.86E-10	HLA-DRA/CD74/HI	13	up
GO:0050867	positive regulation of cell activation	22/143	436/18800	2.07E-12	2.94E-10	2.24E-10	HLA-DRA/CCL5/CI	22	up
GO:0009615	response to virus	21/143	392/18800	2.21E-12	2.96E-10	2.26E-10	CCL5/IFI27/BST2/IS	21	up
GO:0051607	defense response to virus	18/143	290/18800	8.17E-12	1.04E-09	7.92E-10	IFI27/BST2/ISG20/II	18	up
GO:0140546	defense response to symbiont	18/143	291/18800	8.66E-12	1.05E-09	7.97E-10	IFI27/BST2/ISG20/II	18	up
GO:0050870	positive regulation of T cell activation	16/143	223/18800	1.45E-11	1.67E-09	1.27E-09	HLA-DRA/CCL5/CI	16	up
GO:0034341	response to interferon-gamma	13/143	140/18800	5.16E-11	5.67E-09	4.32E-09	CCL5/GAPDH/CD7	13	up
GO:0002460	adaptive immune response based on somatic recombination of immune receptors built from immunoglobulin superfamily domains	19/143	370/18800	5.56E-11	5.76E-09	4.39E-09	HLA-DRA/CD74/HI	19	up
GO:0051251	positive regulation of lymphocyte activation	19/143	371/18800	5.82E-11	5.76E-09	4.39E-09	HLA-DRA/CCL5/CI	19	up
GO:1903039	positive regulation of leukocyte cell-cell adhesion	16/143	245/18800	5.96E-11	5.76E-09	4.39E-09	HLA-DRA/CCL5/CI	16	up
GO:0022409	positive regulation of cell-cell adhesion	17/143	291/18800	8.36E-11	7.77E-09	5.92E-09	HLA-DRA/CCL5/CI	17	up
GO:0007159	leukocyte cell-cell adhesion	19/143	381/18800	9.16E-11	8.12E-09	6.19E-09	HLA-DRA/CCL5/CI	19	up
GO:0048525	negative regulation of viral process	11/143	91/18800	9.41E-11	8.12E-09	6.19E-09	CCL5/BST2/ISG20/I	11	up
GO:1903037	regulation of leukocyte cell-cell adhesion	18/143	344/18800	1.36E-10	1.14E-08	8.66E-09	HLA-DRA/CCL5/CI	18	up
GO:0002440	production of molecular mediator of immune response	17/143	312/18800	2.47E-10	1.99E-08	1.51E-08	HLA-DRA/CD74/HI	17	up

Table S4B. Top 30 GO enrichment analysis in biological process between TCR pattern 1a and pattern 1b, related to Figure S3

ID	Description	GeneRatio	BgRatio	pvalue	p.adjust	qvalue	geneID	Count	direction
GO:0019079	viral genome replication	11/74	134/18800	4.54E-12	5.94E-09	4.84E-09	IFI27/MX1/ISG15/C	11	up
GO:1903900	regulation of viral life cycle	11/74	139/18800	6.79E-12	5.94E-09	4.84E-09	LGALS1/MX1/ISG1	11	up
GO:0019058	viral life cycle	14/74	317/18800	2.07E-11	1.21E-08	9.84E-09	IFI27/LGALS1/MX1	14	up
GO:0050792	regulation of viral process	11/74	159/18800	2.95E-11	1.29E-08	1.05E-08	LGALS1/MX1/ISG1	11	up
GO:0045069	regulation of viral genome replication	9/74	87/18800	5.51E-11	1.61E-08	1.31E-08	MX1/ISG15/CCL5/C	9	up
GO:0045071	negative regulation of viral genome replication	8/74	57/18800	5.53E-11	1.61E-08	1.31E-08	MX1/ISG15/CCL5/C	8	up
GO:0016032	viral process	15/74	418/18800	6.89E-11	1.72E-08	1.40E-08	IFI27/LGALS1/MX1	15	up
GO:0048525	negative regulation of viral process	9/74	91/18800	8.31E-11	1.82E-08	1.48E-08	MX1/ISG15/CCL5/L	9	up
GO:0009615	response to virus	14/74	392/18800	3.42E-10	6.64E-08	5.41E-08	IFI27/IFI44L/MX1/I	14	up
GO:0051607	defense response to virus	12/74	290/18800	1.35E-09	2.24E-07	1.82E-07	IFI27/IFI44L/MX1/I	12	up
GO:0140546	defense response to symbiont	12/74	291/18800	1.41E-09	2.24E-07	1.82E-07	IFI27/IFI44L/MX1/I	12	up
GO:0001906	cell killing	9/74	185/18800	4.54E-08	6.61E-06	5.39E-06	GZMB/GAPDH/HM	9	up
GO:0035456	response to interferon-beta	5/74	32/18800	1.53E-07	2.05E-05	1.67E-05	OAS1/BST2/PLSCR	5	up
GO:0034340	response to type I interferon	6/74	64/18800	1.89E-07	2.37E-05	1.93E-05	IFI27/MX1/ISG15/C	6	up
GO:0045088	regulation of innate immune response	9/74	231/18800	3.02E-07	3.52E-05	2.87E-05	ISG15/CCL5/OAS1/	9	up
GO:0034341	response to interferon-gamma	7/74	140/18800	1.30E-06	0.00014175	0.00011551	GAPDH/CCL5/BST2	7	up
GO:0044403	biological process involved in symbiotic interaction	9/74	286/18800	1.79E-06	0.00018425	0.00015015	IFI27/LGALS1/GAP	9	up
GO:0032479	regulation of type I interferon production	6/74	98/18800	2.39E-06	0.00022019	0.00017944	ISG15/GAPDH/OAS	6	up
GO:0032606	type I interferon production	6/74	98/18800	2.39E-06	0.00022019	0.00017944	ISG15/GAPDH/OAS	6	up
GO:0060337	type I interferon signaling pathway	5/74	56/18800	2.69E-06	0.00023534	0.00019178	IFI27/ISG15/OAS1/	5	up
GO:1903901	negative regulation of viral life cycle	4/74	26/18800	3.10E-06	0.00025508	0.00020786	LY6E/BST2/IFITM2/	4	up
GO:0071357	cellular response to type I interferon	5/74	58/18800	3.21E-06	0.00025508	0.00020786	IFI27/ISG15/OAS1/	5	up
GO:0042267	natural killer cell mediated cytotoxicity	5/74	72/18800	9.39E-06	0.0006887	0.00056122	GZMB/HAVCR2/TU	5	up
GO:0002831	regulation of response to biotic stimulus	9/74	351/18800	9.45E-06	0.0006887	0.00056122	ISG15/CCL5/OAS1/	9	up
GO:0002228	natural killer cell mediated immunity	5/74	75/18800	1.15E-05	0.0008027	0.00065412	GZMB/HAVCR2/TU	5	up
GO:0001909	leukocyte mediated cytotoxicity	6/74	131/18800	1.28E-05	0.00086249	0.00070285	GZMB/HAVCR2/TU	6	up
GO:0045089	positive regulation of innate immune response	6/74	140/18800	1.87E-05	0.00121251	0.00098808	CCL5/HAVCR2/PLS	6	up
GO:0046718	viral entry into host cell	6/74	146/18800	2.37E-05	0.00146533	0.0011941	LGALS1/LY6E/PLS(	6	up
GO:0046596	regulation of viral entry into host cell	4/74	43/18800	2.43E-05	0.00146533	0.0011941	LGALS1/LY6E/IFITN	4	up
GO:0044409	entry into host	6/74	153/18800	3.09E-05	0.0017965	0.00146397	LGALS1/LY6E/PLS(	6	up

Table S4C. Top 30 GO enrichment analysis in biological process between TCR pattern 1 and pattern 2, related to Figure S3

ID	Description	GeneRatio	BgRatio	pvalue	p.adjust	qvalue	geneID	Count	direction
GO:0050863	regulation of T cell activation	19/130	342/18800	2.54E-12	6.08E-09	4.58E-09	CAMK4/LGALS3/C(	19	up
GO:0022407	regulation of cell-cell adhesion	20/130	456/18800	4.71E-11	5.63E-08	4.24E-08	LGALS3/CCL5/HLA	20	up
GO:1903131	mononuclear cell differentiation	19/130	433/18800	1.49E-10	8.98E-08	6.77E-08	CAMK4/HLA-DRA/	19	up
GO:0030098	lymphocyte differentiation	18/130	382/18800	1.50E-10	8.98E-08	6.77E-08	CAMK4/HLA-DRA/	18	up
GO:1903037	regulation of leukocyte cell-cell adhesion	17/130	344/18800	2.44E-10	1.17E-07	8.78E-08	LGALS3/CCL5/HLA	17	up
GO:0030217	T cell differentiation	15/130	263/18800	4.16E-10	1.66E-07	1.25E-07	CAMK4/HLA-DRA/	15	up
GO:0019221	cytokine-mediated signaling pathway	19/130	486/18800	1.03E-09	3.30E-07	2.49E-07	CXCR3/CXCR6/CCL	19	up
GO:0007159	leukocyte cell-cell adhesion	17/130	381/18800	1.16E-09	3.30E-07	2.49E-07	LGALS3/CCL5/HLA	17	up
GO:0051250	negative regulation of lymphocyte activation	12/130	161/18800	1.24E-09	3.30E-07	2.49E-07	LGALS3/CD74/SAN	12	up
GO:0030593	neutrophil chemotaxis	10/130	106/18800	3.20E-09	7.65E-07	5.76E-07	LGALS3/CCL5/JAM	10	up
GO:0016032	viral process	17/130	418/18800	4.70E-09	9.82E-07	7.40E-07	CXCR6/CCL5/JUN/I	17	up
GO:0060326	cell chemotaxis	15/130	315/18800	4.93E-09	9.82E-07	7.40E-07	CXCR3/LGALS3/CX	15	up
GO:0002695	negative regulation of leukocyte activation	12/130	193/18800	9.75E-09	1.79E-06	1.35E-06	LGALS3/CD74/SAN	12	up
GO:0022409	positive regulation of cell-cell adhesion	14/130	291/18800	1.45E-08	2.48E-06	1.87E-06	CCL5/HLA-DRA/CI	14	up
GO:0071621	granulocyte chemotaxis	10/130	128/18800	2.01E-08	3.00E-06	2.26E-06	LGALS3/CCL5/JAM	10	up
GO:1990266	neutrophil migration	10/130	128/18800	2.01E-08	3.00E-06	2.26E-06	LGALS3/CCL5/JAM	10	up
GO:0050866	negative regulation of cell activation	12/130	216/18800	3.42E-08	4.81E-06	3.62E-06	LGALS3/CD74/SAN	12	up
GO:0002683	negative regulation of immune system process	16/130	425/18800	4.05E-08	5.01E-06	3.77E-06	LGALS3/KLRC1/PD	16	up
GO:0019058	viral life cycle	14/130	317/18800	4.25E-08	5.01E-06	3.77E-06	CXCR6/CCL5/IFI27/	14	up
GO:1903706	regulation of hemopoiesis	15/130	371/18800	4.37E-08	5.01E-06	3.77E-06	CAMK4/HLA-DRA/	15	up
GO:0097191	extrinsic apoptotic signaling pathway	12/130	221/18800	4.40E-08	5.01E-06	3.77E-06	LGALS3/ITM2C/IFI2	12	up
GO:0045619	regulation of lymphocyte differentiation	11/130	181/18800	5.23E-08	5.69E-06	4.28E-06	CAMK4/HLA-DRA/	11	up
GO:0045088	regulation of innate immune response	12/130	231/18800	7.16E-08	7.44E-06	5.60E-06	CCL5/KLRC1/HMGI	12	up
GO:0030595	leukocyte chemotaxis	12/130	236/18800	9.04E-08	9.01E-06	6.79E-06	LGALS3/CCL5/JAM	12	up
GO:0045580	regulation of T cell differentiation	10/130	151/18800	9.68E-08	9.26E-06	6.98E-06	CAMK4/HLA-DRA/	10	up
GO:0097530	granulocyte migration	10/130	154/18800	1.17E-07	1.07E-05	8.07E-06	LGALS3/CCL5/JAM	10	up
GO:1903039	positive regulation of leukocyte cell-cell adhesion	12/130	245/18800	1.36E-07	1.20E-05	9.07E-06	CCL5/HLA-DRA/CI	12	up
GO:0001819	positive regulation of cytokine production	16/130	475/18800	1.85E-07	1.58E-05	1.19E-05	CD2/RGCC/CD74/H	16	up
GO:0050868	negative regulation of T cell activation	9/130	125/18800	2.13E-07	1.75E-05	1.32E-05	LGALS3/CD74/PAG	9	up
GO:0032069	regulation of nuclease activity	5/130	21/18800	2.72E-07	2.17E-05	1.64E-05	HMGB1/OASL/HSP	5	up

Table S4D. Top 30 GO enrichment analysis in biological process between P6 cells with and without Influenza B virus reads, related to Figure S4

ID	Description	GeneRatio	BgRatio	pvalue	p.adjust	qvalue	geneID	Count	direction
GO:0034341	response to interferon-gamma	17/154	140/18800	1.80E-15	5.21E-12	3.86E-12	GBP1/IRF1/TLR2/G	17	up
GO:0071347	cellular response to interleukin-1	15/154	111/18800	1.74E-14	2.45E-11	1.82E-11	IL1RN/GBP1/CEBPI	15	up
GO:0019221	cytokine-mediated signaling pathway	26/154	486/18800	2.55E-14	2.45E-11	1.82E-11	IL1RN/CCRL2/CSF1	26	up
GO:0070555	response to interleukin-1	15/154	141/18800	6.34E-13	4.58E-10	3.39E-10	IL1RN/GBP1/CEBPI	15	up
GO:0032496	response to lipopolysaccharide	20/154	333/18800	3.89E-12	2.25E-09	1.66E-09	SOD2/LYN/JUND/T	20	up
GO:0009615	response to virus	21/154	392/18800	9.52E-12	4.58E-09	3.40E-09	GBP1/IRF1/OAS3/N	21	up
GO:0002237	response to virus	20/154	354/18800	1.18E-11	4.88E-09	3.62E-09	SOD2/LYN/JUND/T	20	up
GO:0071346	cellular response to interferon-gamma	13/154	118/18800	1.49E-11	5.37E-09	3.98E-09	GBP1/IRF1/TLR2/G	13	up
GO:0051607	defense response to virus	18/154	290/18800	2.90E-11	8.85E-09	6.56E-09	GBP1/IRF1/OAS3/N	18	up
GO:0140546	defense response to symbiont	18/154	291/18800	3.07E-11	8.85E-09	6.56E-09	GBP1/IRF1/OAS3/N	18	up
GO:0060326	cell chemotaxis	18/154	315/18800	1.13E-10	2.96E-08	2.19E-08	CCRL2/CSF1/LYN/I	18	up
GO:0030595	leukocyte chemotaxis	15/154	236/18800	1.02E-09	2.45E-07	1.81E-07	CSF1/LYN/TREM1/	15	up
GO:0016032	viral process	19/154	418/18800	1.57E-09	3.50E-07	2.59E-07	ICAM1/OAS3/IGF2I	19	up
GO:0034612	response to tumor necrosis factor	15/154	249/18800	2.13E-09	4.38E-07	3.25E-07	GBP1/GBP2/TANK	15	up
GO:0048247	lymphocyte chemotaxis	9/154	64/18800	2.46E-09	4.74E-07	3.51E-07	CXCL16/TNFSF14/(	9	up
GO:0050900	leukocyte migration	18/154	384/18800	2.70E-09	4.87E-07	3.61E-07	ICAM1/CSF1/LYN/	18	up
GO:0071356	cellular response to tumor necrosis factor	14/154	229/18800	6.21E-09	1.05E-06	7.82E-07	GBP1/GBP2/TANK	14	up
GO:0071621	granulocyte chemotaxis	11/154	128/18800	8.32E-09	1.33E-06	9.89E-07	CSF1/TREM1/CXCL	11	up
GO:0030593	neutrophil chemotaxis	10/154	106/18800	1.65E-08	2.50E-06	1.86E-06	TREM1/CXCL10/CX	10	up
GO:0032103	positive regulation of response to external stimulus	18/154	442/18800	2.39E-08	3.45E-06	2.56E-06	CSF1/NEAT1/LYN/	18	up
GO:0071222	cellular response to lipopolysaccharide	13/154	217/18800	2.82E-08	3.87E-06	2.87E-06	LYN/TLR2/CEBPB/I	13	up
GO:0070098	chemokine-mediated signaling pathway	9/154	89/18800	4.77E-08	6.12E-06	4.53E-06	CCRL2/CXCL10/CX(	9	up
GO:0072676	lymphocyte migration	10/154	119/18800	5.03E-08	6.12E-06	4.53E-06	ICAM1/CXCL16/TN	10	up
GO:0071219	cellular response to molecule of bacterial origin	13/154	229/18800	5.31E-08	6.12E-06	4.53E-06	LYN/TLR2/CEBPB/I	13	up
GO:0097529	myeloid leukocyte migration	13/154	229/18800	5.31E-08	6.12E-06	4.53E-06	CSF1/LYN/TREM1/	13	up
GO:1901623	regulation of lymphocyte chemotaxis	6/154	26/18800	5.51E-08	6.12E-06	4.53E-06	TNFSF14/CXCL10/(	6	up
GO:0097530	granulocyte migration	11/154	154/18800	5.72E-08	6.12E-06	4.54E-06	CSF1/TREM1/CXCL	11	up
GO:0002224	toll-like receptor signaling pathway	10/154	123/18800	6.89E-08	7.09E-06	5.25E-06	IRF1/LYN/RSAD2/T	10	up
GO:0001819	positive regulation of cytokine production	18/154	475/18800	7.12E-08	7.09E-06	5.25E-06	IRF1/OAS3/HLA-A	18	up
GO:0002683	negative regulation of immune system process	17/154	425/18800	7.79E-08	7.50E-06	5.56E-06	GBP1/IRF1/LYN/O	17	up

Table S4E. Top 30 GO enrichment analysis in biological process between pneumonia and control PB and PC, related to Figure S5

ID	Description	GeneRatio	BgRatio	pvalue	p.adjust	qvalue	geneID	Count	direction
GO:0050853	B cell receptor signaling pathway	8/25	131/18800	4.38E-12	2.20E-09	1.66E-09	CD38/IGHV6-1/IGH	8	up
GO:0050871	positive regulation of B cell activation	8/25	152/18800	1.46E-11	3.66E-09	2.76E-09	CD38/IGHV6-1/IGH	8	up
GO:0006910	phagocytosis, recognition	7/25	102/18800	4.98E-11	8.34E-09	6.28E-09	IGHV6-1/IGHG1/IG	7	up
GO:0006958	complement activation, classical pathway	7/25	108/18800	7.48E-11	9.39E-09	7.07E-09	IGHV6-1/IGHG1/IG	7	up
GO:0050864	regulation of B cell activation	8/25	200/18800	1.32E-10	1.33E-08	9.98E-09	CD38/IGHV6-1/IGH	8	up
GO:0002455	humoral immune response mediated by circulating immunoglobulin	7/25	121/18800	1.68E-10	1.40E-08	1.06E-08	IGHV6-1/IGHG1/IG	7	up
GO:0006911	phagocytosis, engulfment	7/25	128/18800	2.50E-10	1.79E-08	1.35E-08	IGHV6-1/IGHG1/IG	7	up
GO:0006956	complement activation	7/25	131/18800	2.94E-10	1.84E-08	1.39E-08	IGHV6-1/IGHG1/IG	7	up
GO:0099024	plasma membrane invagination	7/25	137/18800	4.03E-10	2.25E-08	1.69E-08	IGHV6-1/IGHG1/IG	7	up
GO:0042742	defense response to bacterium	9/25	364/18800	5.39E-10	2.61E-08	1.97E-08	ISG15/RNF213/IGH	9	up
GO:0010324	membrane invagination	7/25	144/18800	5.72E-10	2.61E-08	1.97E-08	IGHV6-1/IGHG1/IG	7	up
GO:0050851	antigen receptor-mediated signaling pathway	8/25	244/18800	6.42E-10	2.68E-08	2.02E-08	CD38/IGHV6-1/IGH	8	up
GO:0002429	immune response-activating cell surface receptor signaling pathway	8/25	300/18800	3.27E-09	1.17E-07	8.83E-08	CD38/IGHV6-1/IGH	8	up
GO:0002757	immune response-activating signal transduction	8/25	300/18800	3.27E-09	1.17E-07	8.83E-08	CD38/IGHV6-1/IGH	8	up
GO:0002768	immune response-regulating cell surface receptor signaling pathway	8/25	328/18800	6.59E-09	2.20E-07	1.66E-07	CD38/IGHV6-1/IGH	8	up
GO:0042113	B cell activation	8/25	336/18800	7.95E-09	2.49E-07	1.88E-07	CD38/IGHV6-1/IGH	8	up
GO:0016064	immunoglobulin mediated immune response	7/25	216/18800	9.67E-09	2.86E-07	2.15E-07	IGHV6-1/IGHG1/IG	7	up
GO:0019724	B cell mediated immunity	7/25	219/18800	1.06E-08	2.97E-07	2.23E-07	IGHV6-1/IGHG1/IG	7	up
GO:0008037	cell recognition	7/25	228/18800	1.40E-08	3.71E-07	2.79E-07	IGHV6-1/IGHG1/IG	7	up
GO:0051251	positive regulation of lymphocyte activation	8/25	371/18800	1.72E-08	4.32E-07	3.25E-07	CD38/IGHV6-1/IGH	8	up
GO:0034340	response to type I interferon	5/25	64/18800	1.97E-08	4.70E-07	3.54E-07	IFI27/ISG15/IFITM1	5	up
GO:0002253	activation of immune response	8/25	386/18800	2.34E-08	5.34E-07	4.02E-07	CD38/IGHV6-1/IGH	8	up
GO:0002696	positive regulation of leukocyte activation	8/25	421/18800	4.58E-08	1.00E-06	7.53E-07	CD38/IGHV6-1/IGH	8	up
GO:0050867	positive regulation of cell activation	8/25	436/18800	6.01E-08	1.26E-06	9.46E-07	CD38/IGHV6-1/IGH	8	up
GO:0006909	phagocytosis	7/25	310/18800	1.15E-07	2.32E-06	1.74E-06	IGHV6-1/IGHG1/IG	7	up
GO:0002764	immune response-regulating signaling pathway	8/25	482/18800	1.30E-07	2.50E-06	1.88E-06	CD38/IGHV6-1/IGH	8	up
GO:0006959	humoral immune response	7/25	317/18800	1.34E-07	2.50E-06	1.88E-06	IGHV6-1/IGHG1/IG	7	up
GO:0002449	lymphocyte mediated immunity	7/25	365/18800	3.49E-07	6.26E-06	4.71E-06	IGHV6-1/IGHG1/IG	7	up
GO:0002460	adaptive immune response based on somatic recombination of immune receptors built from immunoglobulin superfamily domains	7/25	370/18800	3.83E-07	6.63E-06	4.99E-06	IGHV6-1/IGHG1/IG	7	up
GO:0060337	type I interferon signaling pathway	4/25	56/18800	8.52E-07	1.43E-05	1.07E-05	IFI27/ISG15/IFITM1	4	up

Table S4. GO enrichment analysis in biological process of DEG in TableS4, related to Figure S2, S3, S4 and S5.

(A) Table S4A. Analysis between pneumonia and control T8_rm, related to Figure S2
(B) Table S4B. Analysis between TCR pattern 1a and pattern 1b, related to Figure S3
(C) Table S4C. Analysis between TCR pattern 1 and pattern 2, related to Figure S3
(D) Table S4D. Analysis between P6 cells with and without Influenza B virus reads, related to Figure S4
(E) Table S4E. Analysis between pneumonia and control PB and PC, related to Figure S5

TableS5. 21-color flow cytometry panel for validation group.

ID	Rationale	Antigen (*:intracellular)	Fluorphore	Clone	Catalog.No	Vender	Laser	Filter	Brightness	Volume per test (/uL)
1	Neutrophil	CD32	BUV395	FLI8.28	740292	BD	355	379/28	2/5	1
2	Live/Dead	L/D	LIVE/DEAD Fixable Biue Dead Cell Stain		L34961	BD	355	450/50 410LP	-	0.5
3	Th	CD4	BUV496	SK3	612936	BD	355	515/30 450LP	2/5	1
4	Memory B	CD27	BUV563	L128	748705	BD	355	580/20 550LP	3/5	1
5	B	CD19	BUV661	HIB19	741604	BD	355	670/25 630LP	3/5	1
6	Memory	CD127	BUV737	HIL-7R-M21	612794	BD	355	735/30 690LP	4/5	2
7	Mono-derived Macro	CX3CR1	BV421	2A9-1	565800	BD	405	450/50 410LP	4/5	3
8	T	CD3	BV510	HIT3a	564713	BD	405	525/50 505LP	2/5	1
9	Monocyte	CD14	BV605	M5E2	301834	BioLegend	405	605/40 595LP	4/5	1
10	DC	CD11c	BV650	B-ly6	563404	BD	405	677/20 635LP	4/5	1
11	Plasma	CD38	BV711	HIT2	563965	BD	405	710/50 685LP	4/5	3
12	Resident / Activation	CD69	BV750	FN50	310954	BD	405	750/30 735LP	2/5	1
13	CTL	CD8	BV786	RPA-T8	563823	BD	405	780/60 750LP	3/5	1
14	Proliferation	Ki67*	FITC	SolA15	11-5698-82	eBioscience	488	530/30 505LP	2/5	1
15	Macro/Neutro	CD11b	PerCP/Cy5.5	M1/70	101228	BioLegend	488	710/50 685LP	2/5	2
16	Effector T	KLRG1	PE	SA231A2	367712	BioLegend	532	586/15 570LP	5/5	2
17	Resident T	CD103	PE/Dazzle™ 594	Ber-ACT8	350224	BioLegend	532	610/20 600LP	5/5	1
18	Effector T	GZMB*	PE/Cy7	QA16A02	372214	BioLegend	532	780/60	4/5	1
19	Effector T	GZMK*	Alexa Fluor® 647	GM26E7	370503	BioLegend	640	671/30	4/5	3
20	Leukocyte	CD45	Alexa Fluor® 700	2D1	368513	BioLegend	640	730/45 690LP	2/5	1
21	Inflammatory Mono	CD16	APC/Cy7	3G8	302018	BioLegend	640	795/70	2/5	1

Table S5. 21-color flow cytometry panel for validation group.

Table S6. mIHC antibodies

Marker	Dilution ratio	Channel	Related figure
CD8	1:200	480	Figure 2H
CD103	1:100	520	Figure 2H
GZMB	1:100	570	Figure 2H
CD8	1:200	480	Figure 4G
CD103	1:100	520	Figure 4G
GZMB	1:100	570	Figure 4G
EMP2	1:100	690	Figure 4G
Influenza B Virions	1:100	780	Figure 4G
Ki67	1:200	620	Figure 5G
IgA	1:200	690	Figure 5G
CD138	1:100	780	Figure 5G
Ki67	1:200	620	Figure 5H
IgA	1:200	690	Figure 5H
FOXJ1	1:200	520	Figure 5H
CD68	1:500	480	Figure 6F
FABP4	1:100	570	Figure 6F
CXCL10	1:500	690	Figure 6F

Table S6. mIHC antibodies.

Table S7. Biopsy sample digestion enzymes, related to Methods.

cocktail step1

enzyme	source	identifier	final concentration (mg/mL)
Collagenase Type I	gibco	17100-017	0.2
Collagenase Type IV	gibco	17104-019	0.2
Dispase	gibco	17105-041	0.5
DNase I	Roche	10104159001	0.1

cocktail step2

enzyme	source	identifier	final concentration (mg/mL)
Collagenase Type I	gibco	17100-017	0.5
Collagenase Type IV	gibco	17104-019	0.5
Dispase	gibco	17105-041	1
DNase I	Roche	10104159001	0.1

Table S7. Enzymes for biopsy sample digestion.
